# Randomized Phase I/II Clinical Trial of a Melanoma Helper Peptide Vaccine with or without Systemic Agonistic Anti-CD27 Antibody (Varlilumab)

**DOI:** 10.1158/2767-9764.CRC-25-0744

**Published:** 2026-04-30

**Authors:** Emily K. Ninmer, Gina R. Petroni, Elizabeth Gaughan, Andrew S. Poklepovic, Varinder Kaur, Kathleen Haden, Kimberly A. Chianese-Bullock, Kelly T. Smith, Paul Wright, Jennifer L. Bryant, David Brighton, Jack A. Engel, Timothy N.J. Bullock, Craig L. Slingluff

**Affiliations:** 1 Division of Surgical Oncology, Department of Surgery, and the Human Immune Therapy Center, University of Virginia, Charlottesville, Virginia.; 2 Department of Public Health Sciences, University of Virginia, Charlottesville, Virginia.; 3 Division of Hematology and Oncology, Department of Medicine, University of Virginia, Charlottesville, Virginia.; 4 Division of Hematology and Oncology, Department of Medicine, Virginia Commonwealth University, Richmond, Virginia.; 5 University of Virginia Comprehensive Cancer Center, Charlottesville, Virginia.; 6 Department of Pathology, University of Virginia, Charlottesville, Virginia.

## Abstract

**Purpose:**

This randomized phase I/II clinical trial (NCT03617328) was designed to test whether administration of systemic agonistic anti-CD27 antibody (varlilumab) concurrent with a melanoma vaccine is safe and enhances vaccine immunogenicity.

**Patients and Methods:**

Adults with definitively treated, high-risk melanoma were randomized to receive a shared antigen vaccine targeting CD4^+^ T cells (6 melanoma helper peptides; 6MHP) either with (arm A) or without (arm B) systemic varlilumab over 11 weeks. A final vaccine was given at week 25 to assess memory response.

**Results:**

Thirty-three participants were treated at two centers. After enrolling 17 participants, the dose-limiting toxicity (DLT) rate with vaccine alone required protocol revision to limit local toxicity. Overall, DLT rates for arms A and B were 6% (1/17) and 31% (5/16), respectively. Twenty (61%) participants had CD4^+^ T-cell responses *ex vivo*. Persistent responses were detected in two (2/14, 14%) on arm A and one (1/16, 6%) on arm B. Memory response was detected in two (2/13, 15%) on arm A and four (4/16, 25%) on arm B. On arm A, there was a significant reduction in circulating CD4^+^ T cells. Four-year disease-free survival rates for arms A and B were 20% [95% confidence interval (CI), 6%–67%] and 69% (95% CI, 49%–96%), respectively.

**Conclusions:**

No synergistic toxicity was observed with combination treatment. Similar durability in immunogenicity was found with or without varlilumab. Depletion of circulating CD4^+^ T cells with varlilumab may have abrogated benefits of inducing tumor-cognate CD4^+^ T cells with vaccination. Induction of memory responses supports further work to optimize shared antigen vaccines in combination with other immunotherapies.

**Significance:**

Combination treatment with 6MHP vaccination and varlilumab was safe but did not improve CD4^+^ T-cell response rates. Circulating CD4^+^ T cells were reduced with varlilumab, and clinical outcome was improved without varlilumab. This study demonstrates induction of memory responses after shared antigen vaccination; however, CD27 agonism did not enhance durability of response. Depletion of CD4^+^ T cells after varlilumab may have interfered with the beneficial effects of vaccination.

## Introduction

Immune checkpoint inhibitors (ICI) block checkpoint molecules on T cells to promote tumor control by reestablishing activation signals. Less studied are costimulatory molecules, including CD27, which promote T-cell activation and survival to support antitumor responses. In preclinical models, CD27 agonism lowered PD-1 expression by CD8^+^ T cells (1) and synergized with anti–PD-1 antibodies to enhance T-cell activation (2). Also, CD27 agonism increased circulating CD4^+^ and CD8^+^ T cells with enhanced IFNγ production (3) and has promoted survival of CD4^+^ effector T cells in response to self-antigen (4). In early clinical trials, an agonistic anti-CD27 antibody (varlilumab, Celldex Therapeutics) was safe, reduced circulating regulatory T cells (Treg) with a trend to greater proportions of circulating CD4^+^ T cells with effector memory phenotypes, and had clinical activity either as monotherapy (5) or when combined with anti–PD-1 therapy (6). CD27 agonism combined with cancer vaccination has shown promise in preclinical models (7, 8); however, there has been limited study in humans. In a pilot study of glioma patients, systemic varlilumab combined with a vaccine targeting tumor-associated antigens for CD8^+^ and CD4^+^ T cells was safe and induced effector memory T-cell responses (9).

CD8^+^ T cells were the target of early cancer vaccines, but CD4^+^ T cells are now recognized to have important roles in antitumor immunity (10). We have reported that a vaccine composed of six tumor-associated class II MHC–restricted antigens (6 melanoma helper peptides; 6MHP) is immunogenic and has clinical activity as monotherapy (11, 12), in combination with anti–PD-1 therapy (13), or with a vaccine also targeting CD8^+^ T cells (14–16). In some of these prior trials, we found that patients with peripheral immune responses to 6MHP had improved clinical outcomes compared with those without an immune response (12, 14). However, many patients have transient circulating T-cell responses. Thus, there is a need to understand whether cancer vaccines induce T-cell memory and whether targeting costimulatory molecules on T cells enhances the durability of immune response. Given the role of CD27 agonism in the generation of T-cell memory (17), we investigated the impact of CD27 costimulation on the persistence of the CD4^+^ T-cell response to 6MHP and memory recall response to 6MHP.

We report the results of an open-label, randomized phase I/II clinical trial (NCT03617328) designed to test the safety and immunogenicity of 6MHP with or without concurrent administration of systemic varlilumab. We hypothesized that CD27 agonism with helper peptide vaccination would be safe, that this treatment combination would enhance the durability of peripheral CD4^+^ T-cell response and decrease Tregs, and that the vaccine would induce memory T-cell responses.

## Patients and Methods

This trial was performed with approval of the lead Institutional Review Board (IRB) at the University of Virginia (HSR-IRB# 20085), with acceptance of the protocol and all amendments at the second participating site (Virginia Commonwealth University). The trial is registered with Clinicaltrials.gov (NCT03617328) and performed with FDA approval (IND# 10825). All participants gave written informed consent prior to participation. The trial was conducted in accordance with the ethical principles of Good Clinical Practice and applicable United States regulations.

### Participants

Patients at least 18 years of age were eligible for enrollment at two academic centers if they had newly diagnosed or recurrent American Joint Committee on Cancer (AJCC, eighth edition) stage IIB to IV melanoma arising from cutaneous, mucosal, ocular, or unknown primary sites and were rendered clinically free of disease within the prior 6 months. Patients with stage IIA cutaneous or ocular melanoma were eligible if considered high-risk on gene expression profiling by DecisionDx or DecisionDx-UM, respectively (Castle Biosciences). Exclusion criteria included ICIs within 12 weeks of registration or chemotherapy, radiation, targeted therapy, or immunomodulatory medications within 4 weeks of registration. Complete eligibility criteria are provided in the protocol (Supplementary File S1).

### Study design

**Figure 1.**
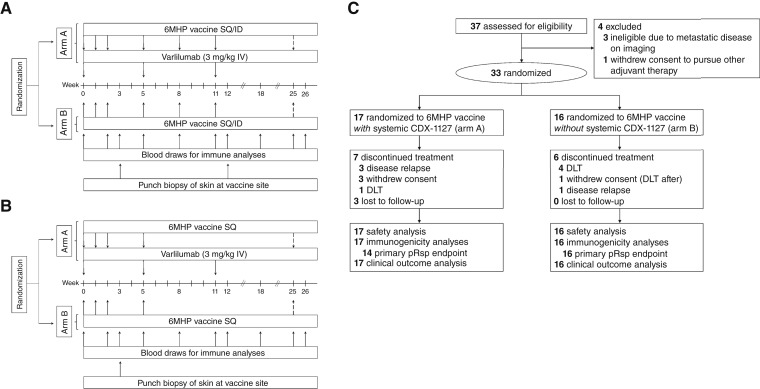
Trial design and protocol amendment for safety. **A** and **B,** Trial schema of study interventions (**A**) before and (**B**) after protocol amendment for exceeding the DLT threshold. Vaccines included poly-ICLC in the adjuvant at all time points except for week 25, represented by the dashed arrow. Blood draws were obtained prior to vaccination and/or varlilumab therapy. **C,** CONSORT flow diagram. ID, intradermal; IV, intravenous; SQ, subcutaneous.

Participants were randomized 1:1 to receive a series of 6MHP vaccines plus varlilumab (arm A) or 6MHP alone (arm B). No stratification factors were used for randomization. Vaccines were prepared as a mixture of the six synthetic peptides (200 μg each; ref. 11) and 0.9 mg of the Toll-like receptor 3 agonist, poly-ICLC (Hiltonol, Oncovir), emulsified in incomplete Freund’s adjuvant (IFA; Montanide ISA-51, Seppic, Inc.). The original study design (Fig. 1A) was amended (Fig. 1B) to reduce local dose-limiting toxicities (DLT). Participants enrolled on the original protocol received a total of seven vaccines, including three weekly priming doses followed by three booster doses given at 3-week intervals through week 11 and a final booster dose at week 25. Participants enrolled on the amended protocol received a total of five vaccines, including three weekly priming doses followed by a booster dose at week 5 and a final booster dose at week 25. The final booster vaccine on either protocol omitted poly-ICLC. All vaccines were administered at the same skin site, as a half-subcutaneous/half-intradermal injection (original protocol) or subcutaneous only injection (amended protocol). Participants on arm A received three intravenous 3 mg/kg doses of varlilumab every 5 to 6 weeks (5, 6) starting at vaccine initiation (week 0) through week 11. Peripheral blood samples were collected at scheduled intervals for immune analyses. Vaccine site punch biopsies were obtained at week 3 and week 12 on the original protocol and only at week 3 on the amended protocol.

### Objectives

Primary endpoints were vaccine safety and to determine whether addition of systemic varlilumab to the vaccine regimen improved the persistence of the peripheral CD4^+^ T-cell response. Secondary endpoints included an assessment of memory CD4^+^ T-cell responses, changes in circulating Tregs by treatment arm, and changes in Tregs in the vaccine site microenvironment (VSME). Exploratory endpoints included assessment of IgG antibody responses to vaccine and evaluation of clinical outcomes. Analyses of the vaccine site biopsies will be reported separately.

### Safety assessment

At each study visit, adverse events (AE) were assessed based on participant toxicity diaries, participant interviews, and review of clinical laboratory tests by a study clinician. AEs were evaluated using the National Cancer Institute Common Terminology Criteria for Adverse Events v5.0. AEs considered definitely, probably, or possibly related to treatment were considered treatment-related AEs (TRAE). DLTs were defined as TRAEs that met the following criteria: ≥ grade 2 ocular toxicity (night blindness, papilledema, or retinopathy or >5 days of decreased vision, flashing lights, or floaters), ≥ grade 2 symptomatic pneumonitis with shortness of breath >5 days, ≥ grade 2 allergic/autoimmune reactions, or any ≥ grade 3 toxicity, including immune-related AEs. The following high-grade (≥ grade 3) AEs were excluded from DLT criteria: grade 3 inflammation due to local antitumor reactions ≤7 days in duration, grade 3 injection site reaction with ulceration ≤2 cm in diameter at the greatest dimension, grade 3 vaccination complications with necrosis, erythema ≤20 cm, induration/swelling ≤20 cm, grade 3 or 4 infusion reactions not representing an allergic/anaphylactic reaction and safe for restart of infusion for continuation of treatment, grade 3 lymphopenia or grade 4 lymphopenia that improved to ≤ grade 3 or within 20% of baseline within 28 days from the last dose of varlilumab, grade 3 neutropenia or grade 4 neutropenia <7 days in duration, and grade 3 nausea, vomiting, or diarrhea that resolved to ≤ grade 1 with or without treatment within 48 hours. The trial was designed with stopping rules for DLT rates beyond those expected with vaccine or varlilumab alone, as described in the protocol (Supplementary File 1).

### Assessment of T-cell response

Peripheral CD4^+^ T-cell responses to the 6MHP pool were evaluated by *ex vivo* IFNγ ELISpot assay of peripheral blood mononuclear cells (PBMC) as described (18). Briefly, viably cryopreserved PBMCs were thawed and then plated at 200,000 viable cells per well and pulsed with peptide (10 μg/mL of each peptide in 6MHP) in quadruplicate. Negative controls were no peptide and irrelevant short and long peptides from the HIV gag protein (short gag and long gag; 10 μg/mL each). Positive controls were a mixture of viral peptides (CEF peptide pool at 2 μg/mL) and phorbol myristate acetate–ionomycin and phytohaemagglutinin. Plates were read using an automated plate reader (Bioreader; Biosys). The number of responding cells per 100,000 CD4^+^ T cells was calculated using the proportion of CD3^+^CD4^+^ cells among live PBMCs by flow cytometry. Assay consistency was evaluated by interassay coefficients of variation (CV) calculated for the response of two normal donors to the CEF peptide pool. For the high responder, the CV was 18% (mean 240 spots/100,000 cells). For the low responder, the CV was 32% (mean 16 spots/100,000 cells).

T-cell response (yes/no) on IFNγ ELISpot assay was evaluated at each time point using the following definitions: N_vax_ = number of T cells responding to vaccine peptides, N_neg_ = number of T cells responding to maximum negative control, and R_vax_ = N_vax_/N_neg_. A positive response (Rsp) required that all of the following criteria were met: (i) N_vax_ exceeded N_neg_ by ≥20/100,000 CD4^+^ T cells (0.02%), (ii) (N_vax_ − 1 SD) > (N_neg_ + 1 SD), and (iii) R_vax_ after vaccination ≥5 times the R_vax_ before vaccination. Continuous measures of immune response denoted as fold increase required that all response criteria (i–iii) were met and were represented by R_vax_. Maximum negative control values of zero were set to 0.2 spots/100,000 CD4^+^ T cells to avoid division by zero for calculating fold increases. Fold increases <1 were set to 1 to indicate no response and to prevent overinflating adjusted fold increases over prevaccine ratios <1, while not affecting the determination of response.

Different response types were defined to provide an analytic framework for assessing immunogenicity across multiple blood draws and to focus on functional recall to characterize T-cell memory. A durable response (dRsp) required a Rsp at two consecutive time points through week 12. A persistent response (pRsp) required a dRsp plus a Rsp at week 18 and/or week 25. A memory response (mRsp) was defined as a Rsp by week 12 plus a Rsp at week 26 after booster vaccination at week 25 that was at least twofold greater than any prebooster week 25 reactivity. For the pRsp and mRsp endpoints, participants without evaluable samples who stopped treatment either due to DLTs or recurrence were considered response failures.

### Assessment of changes in circulating T cells

When viably cryopreserved PBMCs were thawed for ELISpot assays, 200,000 cells were also evaluated by flow cytometry to assess for changes in circulating T-cell populations over time, as previously described (18). Briefly, Live/Dead Aqua viability marker (Thermo Fisher Scientific #L34965) was used at 1:600 for 30 minutes at 4°C for live/dead discrimination. The antibodies used for surface staining were: CD3 V450 (BD Biosciences #560365; RRID: AB_1645570), CD8 PerCP (BioLegend #301030; RRID: AB_893421), CD4 FITC (BD Biosciences #555346; RRID: AB_395751), CD25 PE (Thermo Fisher Scientific #12-0257-42; RRID: AB_2043825), CD127 R718 (BD Biosciences #566967; RRID: AB_2869977), CD14 PE/Dazzle (BioLegend #302270; RRID: AB_2832581), and CD19 Spark NIR 685 (BioLegend #302270; RRID: AB_2832581). All antibodies were titrated for optimal concentrations under the staining conditions, with surface staining performed for 45 minutes at 4°C. The eBioScience FoxP3 Fix/Perm Kit (Thermo Fisher Scientific #00-5523-00) was used to stain for FoxP3 and Ki67 after surface staining. Cells were fixed for 30 minutes at 4°C, permeabilized with the included buffer, and then washed and stained for FoxP3 APC (Thermo Fisher Scientific #17-4776-42; RRID: AB_1603280) and Ki67 BV750 (BioLegend #350536; RRID: AB_2910400), followed by an incubation period of 45 minutes at 4°C. Cells were acquired on an Aurora Borealis 5-Laser flow cytometer (Cytek), and data were analyzed with FCS Express (*De Novo* Software). Tregs were defined as CD3^+^CD4^+^CD25^hi^CD127^lo/−^FoxP3^+^ (Supplementary Fig. S1).

### Assessment of IgG antibody response

IgG antibody responses to the 6MHP pool were evaluated by ELISA, as previously described (18). Briefly, vaccine peptides were diluted in carbonate/bicarbonate buffer and plated for overnight incubation at 4°C. The HIV gag peptide was used as a negative control. Plates were then washed with phosphate-buffered saline with 0.1% Tween 20 (TPBS) and blocked with 5% nonfat dry milk in TPBS for incubation at room temperature for 2 hours. Participant serum was prepared in assay buffer of TPBS with 2% normal goat serum in a fourfold dilution series beginning at 1:100 for incubation overnight at 4°C. Serum from two healthy donors was used as a negative control, and serum from two participants enrolled on a prior 6MHP vaccine trial (NCT02425306; IRB #17680) with known IgG antibody responses to 6MHP served as positive controls. Plates were then washed with TPBS and incubated at room temperature for 1 hour after addition of secondary antibody (goat anti-human IgG AP conjugate, Southern Biotech; RRID: AB_2795643). Plates were then washed with TPBS, and AttoPhos substrate (Promega, Fisher Scientific) was added for incubation at room temperature for 30 minutes in the dark, after which 3N NaOH was used to stop the reaction. Fluorescence was obtained on a Molecular Devices SpectraMax Gemini EM plate reader (excitation 450 nm, emission 580 nm).

IgG titers were estimated as the reciprocal of the serum dilution that yielded a fluorescence intensity 10 times greater than the threshold for reactivity (average fluorescence over the four dilutions) of the negative control (healthy donor) serum, as previously described (19). A positive response required a minimum titer of 100 and at least fourfold greater than the titer for the negative control peptide and any baseline reactivity (18). Titers less than 1 were set to 1 for data visualization without affecting the determination of response.

### Evaluation of clinical outcomes

Participants were followed for clinical outcomes, with the frequency of clinical follow-up and surveillance imaging determined by the treating oncologist according to standard of care. Overall survival (OS) was defined from date of enrollment to date of death from any cause; living participants were censored at last known date alive. Disease-free survival (DFS) was defined from date of enrollment to date of first disease recurrence, including new primaries, or death from any cause; living participants without recurrence were censored at date of last known disease status.

### Statistical analysis

The target sample size was 30 participants evaluable for the primary immunologic endpoint. Point estimates for immune response rates were reported, with the difference in rates between arms calculated with 90% confidence intervals (CI) using the Miettinen–Nurminen score method with the sasLM package (v0.10.5) in R (v4.5.0; RRID: SCR_001905). An observed improvement in pRsp rates with varlilumab in the range of 28% to 31% would support further study. Changes in circulating Tregs were evaluated by repeated measures modeling in SAS (v9.4; RRID: SCR_008567) using a heterogeneous autoregressive 1 covariance matrix on log-transformed data and F-tests based on contrasts to assess specified comparisons, with *P* < 0.05 considered statistically significant. The FORECAST function in Microsoft Excel was used to estimate IgG titers. Clinical outcomes for the intention-to-treat population were evaluated by the Kaplan–Meier method. Survival analysis and generation of Kaplan–Meier curves were performed using the survival (v3.8-3) and ggsurvfit (v1.1.0) packages in R (v4.5.0; RRID: SCR_001905).

## Results

Of 37 patients screened, 33 were randomized between September 2019 and April 2023: 17 to arm A and 16 to arm B. All received at least one vaccine and one varlilumab dose, if applicable (Fig. 1C). Twenty (61%) completed all protocol treatment. All participants were evaluable for safety, and 30 were evaluable for the primary immunologic endpoint (pRsp). Baseline characteristics were balanced by arm (Table 1). Eighteen (55%) were male, and the median (range) age was 65 (31–82) years. Seven (21%) received prior systemic therapy (all ICIs, plus one also with BRAF/MEK inhibition). A study representativeness table is included as Supplementary Table S1.

**Table 1.** Participant baseline characteristics by treatment arm.

​	Arm A (*N* = 17)	Arm B (*N* = 16)	Total (*N* = 33)
Age, median (range) years	68 (39–77)	64 (31–82)	65 (31–82)
Male, *n* (%)	9 (53)	9 (56)	18 (55)
Race^a^, *n* (%)	​	​	​
White	16 (94)	16 (100)	32 (97)
Black	1 (6)	0 (0)	1 (3)
Institution, *n* (%)	​	​	​
University of Virginia	13 (76)	12 (75)	25 (76)
Virginia Commonwealth University	4 (24)	4 (25)	8 (24)
Primary site, *n* (%)	​	​	​
Cutaneous	12 (71)	11 (69)	23 (70)
Mucosal	2 (12)	0 (0)	2 (6)
Ocular	3 (18)	4 (25)	7 (21)
Unknown	0 (0)	1 (6)	1 (3)
AJCC stage, *n* (%)	​	​	​
Cutaneous and unknown primary	​	​	​
IIA	1 (6)	1 (6)	2 (6)
IIB	4 (24)	4 (25)	8 (24)
IIC	1 (6)	0 (0)	1 (3)
IIIA	1 (6)	1 (6)	2 (6)
IIIB	2 (12)	1 (6)	3 (9)
IIIC	2 (12)	1 (6)	3 (9)
IV	1 (6)	4 (25)	5 (15)
Ocular	​	​	​
IIB	1 (6)	1 (6)	2 (6)
IIIA	2 (12)	3 (19)	5 (15)
Mucosal	​	​	​
III	2 (12)	0 (0)	2 (6)
Recurrent disease at enrollment, *n* (%)	3 (18)	5 (31)	8 (24)
ECOG performance status, *n* (%)	​	​	​
0	12 (71)	12 (75)	24 (73)
1	5 (29)	4 (25)	9 (27)
Prior systemic therapy, *n* (%)	3 (18)	4 (25)	7 (21)

Abbreviations: AJCC, American Joint Committee on Cancer, eighth edition; ECOG PS, Eastern Cooperative Oncology Group performance status; NOS, not otherwise specified.

a All participants identified as non-Hispanic ethnicity.

### Safety

Among 17 participants on arm A (6MHP + varlilumab), there was one DLT (6%; grade 3 skin ulceration). The DLT rate for arm A never crossed the safety bound. For arm B (6MHP only), the DLT rate crossed the safety bound twice. After enrolling five participants, two DLTs were reported (grade 2 pneumonitis and grade 3 skin ulceration), and enrollment was held briefly to modify the protocol to exclude participants with prior pneumonitis. After enrolling eight participants, the DLT rate again crossed the safety bound on arm B (grade 3 skin ulceration), and enrollment was held for a major protocol redesign. During this time, one additional participant already accrued to arm B experienced a DLT (grade 3 skin ulceration). On December 3, 2020, the protocol was amended to limit local toxicity by reducing the number of vaccines, limiting skin biopsies to week 3 only and altering vaccine administration to subcutaneous injection only (Fig. 1B). After this amendment, enrollment resumed, and there were no DLTs on arm A and one on arm B (grade 3 vision changes with retinal detachment). All DLTs were attributed to vaccine (Table 2). Overall, the DLT rate was 18% (6/33), with one (6%) and five (31%) on arms A and B, respectively (Supplementary Table S2).

**Table 2.** DLTs. Summary of all DLTs reported in the trial.

Patient ID	Arm	Protocol amendment	Adverse event	Days on treatment	Relatedness	Grade
1311	A	Before	Skin ulceration	126	Definite (vaccine); unrelated (varlilumab)	3
1320	B	Before	Pneumonitis	159	Probable (vaccine)	2
1324	B	Before	Skin ulceration	19^a^	Definite (vaccine)	3
1333	B	Before	Skin ulceration	47	Definite (vaccine)	3
1335	B	Before	Skin ulceration	68	Definite (vaccine)	3
1374	B	After	Blurred vision; Decreased vision; Retinal detachment	43	Definite (vaccine)	3

a Participant withdrew consent for treatment at day 19 due to pain, redness, and swelling at the vaccine site that did not meet DLT criteria; however, later developed ulceration on follow-up at day 60 after enrollment (40 days after last vaccine treatment before withdrawal of consent).

All TRAEs were ≤ grade 3, and most were grade 1 to 2 (Supplementary Table S3). The most common TRAEs were injection site reactions (94%), skin induration (48%), fatigue (45%), lymphopenia (36%), flu-like symptoms (27%), myalgia (24%), and skin ulceration (24%). TRAEs were similar by arm, except lymphopenia (53% arm A; 19% arm B). No deaths occurred on active treatment. TRAEs by protocol amendment are provided in Supplementary Tables S4 and S5. All AEs are provided in Supplementary Table S6.

### T-cell response

**Figure 2.**
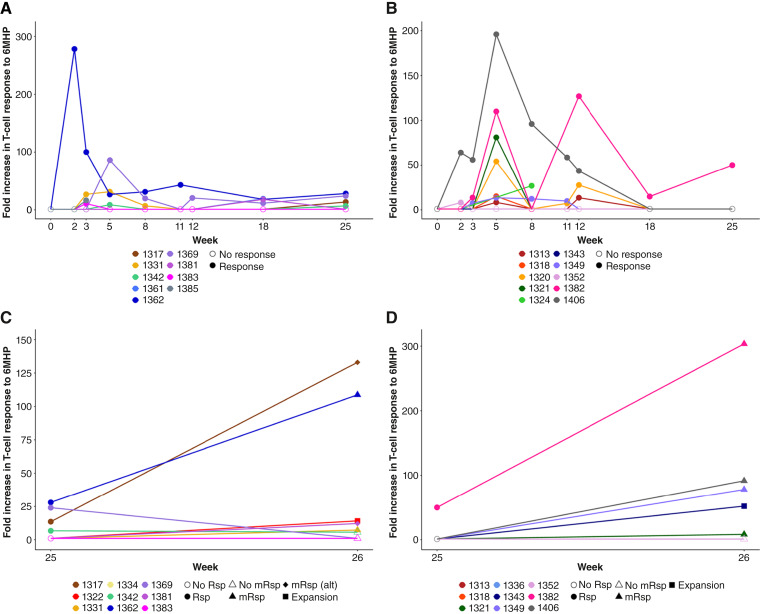
T-cell responses. **A** and **B,** T-cell responses to 6MHP in PBMC by *ex vivo* IFNγ ELISpot assay for participants who had at least one response (Rsp) through week 25 on (**A**) arm A (*n* = 9) and (**B**) arm B (*n* = 10). One participant on arm A (ID 1322) only had a response at week 26 and is not shown. Each solid line represents a participant by a unique identifier number. A fivefold increase over background, corrected for any preexisting reactivity, was required for a response, in addition to at least 20 spots/10^5^ CD4^+^ T cells over maximum negative control and no overlapping standard deviations. Small fold increases over background not meeting response criteria are shown as a fold increase of 1 (no response). **C** and **D,** Memory response evaluation in PBMCs by *ex vivo* IFNγ ELISpot assay for participants with evaluable samples at weeks 25 and 26 on (**C**) arm A (*n* = 9) and (**D**) arm B (*n* = 9). One participant on arm B (ID 1330) was missing a week 25 sample and had no response at week 26 and was thus considered evaluable for memory response but is not shown. Protocol-defined memory response (mRsp) is shown as a solid-filled triangle marker at week 26, whereas the alternative (alt) definition for evidence of memory response is shown as a solid-filled diamond marker at week 26. Participants that had T-cell expansion at week 26 after booster vaccine at week 25 but did not meet either the mRsp or mRsp (alt) criteria are shown by the solid-filled square marker at week 26.

Among all 33 participants, 20 (61%) had at least one Rsp at any time point, and seven (21%) had a dRsp (Table 3; Fig. 2A–D). For the primary immunologic endpoint (pRsp), three participants were unevaluable. Only three (10%) participants met the criteria for pRsp by the stringent protocol definition (A − B: +8%, 90% CI, −12% to +31%; Table 3). However, the protocol definition for pRsp specified that those without samples at week 18 or week 25 due to stopping treatment due to a DLT or disease recurrence were considered response failures. Thus, we performed a *post hoc* analysis of the 25 participants with an evaluable sample at either week 18 or week 25. Among these participants, eight (32%) had at least one Rsp at both an early (by week 12) and late (after week 12) time points, consistent with persistence of response (Table 3).

**Table 3.** T-cell responses by treatment arm and response type. Response rates are reported as the percentage of responses (*n*) out of participants (*N*). The difference in response rates (A − B) is shown with 90% CI.

​	Any response (Rsp)	Durable response (dRsp)	Persistent response (pRsp)	Early + late response	Memory response (mRsp)	Alternative memory response	Expansion at week 26
Population	Protocol-defined	Protocol-defined	Protocol-defined	Evaluable	Protocol-defined	Evaluable	Evaluable
Arm	*n*/*N* (%)	*n*/*N* (%)	*n*/*N* (%)	*n*/*N* (%)	*n*/*N* (%)	*n*/*N* (%)	*n*/*N* (%)
A	10/17 (59)	3/17 (18)	2/14 (14)	4/13 (31)	2/13 (15)	4/9 (44)	5/9 (56)
B	10/16 (63)	4/16 (25)	1/16 (6)	4/12 (33)	4/16 (25)	4/10 (40)	5/10 (50)
Total	20/33 (61)	7/33 (21)	3/30^a^ (10)	8/25 (32)	6/29^b^ (21)	8/19 (42)	10/19 (53)
A − B (90% CI)	−4% (−31% to +24%)	−7% (−32% to +17%)	+8% (−12% to +31%)	−2% (−33% to +28%)	−10% (−34% to +17%)	+4% (−32% to +40%)	+6% (−31% to +41%)

a Three of 33 were not evaluable for pRsp due to no sample at week 18 or week 25. Of the 30 evaluable, five were considered response failures due to DLTs (*n* = 4) or recurrence (*n* = 1) despite no samples drawn at week 18 or week 25. Two participants experienced late DLTs but had evaluable samples for pRsp at week 18 or week 25.

b Of the 30 participants who were evaluable for pRsp, one was excluded from evaluation for mRsp due to having a missing week 25 sample but positive response at week 26 and thus was unevaluable for a twofold increase in response.

Of the 30 participants evaluable for pRsp, 29 were evaluable for mRsp. By the protocol definition, six (21%) had a mRsp (A − B: −10%, 90% CI, −34% to +17%; Table 3; Fig. 2C and D). Given potential limitations of the protocol-defined criteria that may not reliably capture the breadth of responses across participants, we also explored alternative definitions. On *post hoc* analysis of the 19 with evaluable samples at week 26 who were also not missing week 25 samples necessary for determination of response (twofold increase), eight (42%) had at least one Rsp prior to week 26 and at least twofold over the response at week 25, consistent with a memory response (alternative memory response in Table 3; Fig. 2C and D). Ten (53%) had a twofold increase in response at week 26 compared with week 25, regardless of any prior response, consistent with expansion after booster vaccination (Table 3; Fig. 2C and D). Overall, CD4^+^ T-cell response rates by IFNγ ELISpot were similar by arm (Table 3). Immune response rates are summarized by protocol amendment in Supplementary Table S7.

### Changes in circulating Tregs

**Figure 3.**
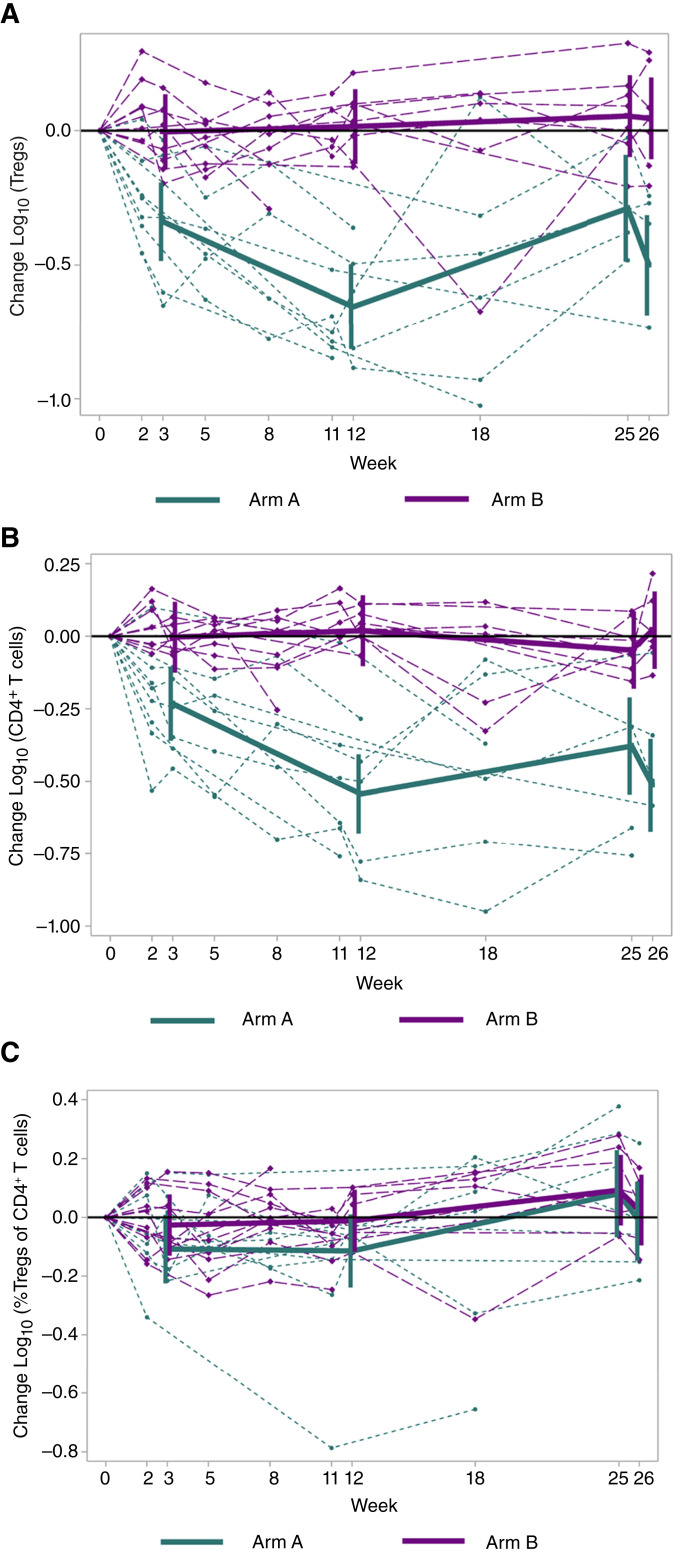
Impact of treatment on circulating CD4^+^ T cells over time. Participants (arm A *n* = 10; arm B *n* = 12) were evaluated for changes in circulating T cells from baseline by repeated measures modeling of log_10_-transformed data: (**A**) absolute number of Tregs, (**B**) absolute number of CD4^+^ T cells, and (**C**) Tregs as a percentage of CD4^+^ T cells. Each participant is represented by a dashed line, and the solid lines represent the model estimated change from baseline with 95% CI for each arm.

Prior work has shown that systemic varlilumab decreases circulating Tregs (5, 6). To assess for this, PBMCs were evaluated by flow cytometry from baseline through week 26. Low-viability samples (<70%) were excluded. Eleven participants without a baseline sample (absent, *n* = 2; low viability, *n* = 9) were excluded entirely; thus, 10 and 12 participants were evaluable on arms A and B, respectively. We found significantly decreased circulating Tregs and total CD4^+^ T cells over time for participants on arm A compared with arm B at all specified time points; no significant differences in circulating Tregs as a percentage of CD4^+^ T cells were found between arms (Fig. 3A–C; Supplementary Tables S8–S10). After the final booster vaccination (week 25 to week 26), participants on arm B had a significant increase in circulating CD4^+^ T cells and significant decrease in circulating Tregs as a percentage of CD4^+^ T cells (Fig. 3B and C; Supplementary Tables S9 and S10).

### IgG antibody response

In prior work, we have found strong IgG antibody responses to 6MHP after vaccination with IFA-based formulations, including adjuvant formulations with local granulocyte–macrophage colony-stimulating factor (12), as well as with local poly-ICLC with or without systemic metronomic cyclophosphamide (18). In the present study, IgG responses to the 6MHP pool were detected in all participants with evaluable serum samples (*n* = 31; Supplementary Fig. S2). For participants on both arms, titers generally increased to week 12 and then persisted at levels >1,000 through the last evaluable time point at week 25.

### Clinical outcomes

**Figure 4.**
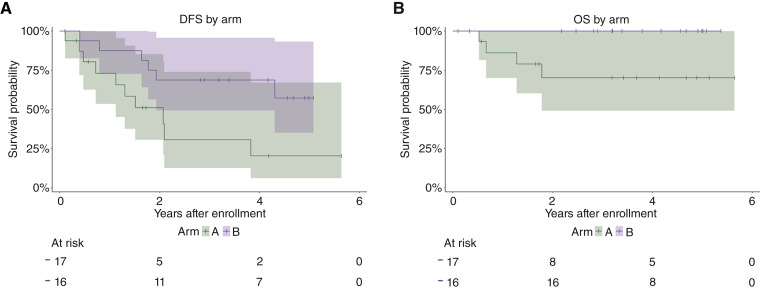
Clinical outcomes by arm. Kaplan–Meier survival curves with 95% CI by arm for (**A**) DFS and (**B**) OS.

Median clinical follow-up was 3.4 years. As of May 2025, 16 (48%) participants experienced melanoma recurrence. The 4-year DFS rates (95% CI) for arms A and B were 20% (6%–67%) and 69% (49%–96%), respectively (Fig. 4A). Four (12%) participants, all on arm A, have died of advanced melanoma. The 4-year OS rates (95% CI) for arms A and B were 70% (49%–100%) and 100%, respectively (Fig. 4B).

**Figure 5.**
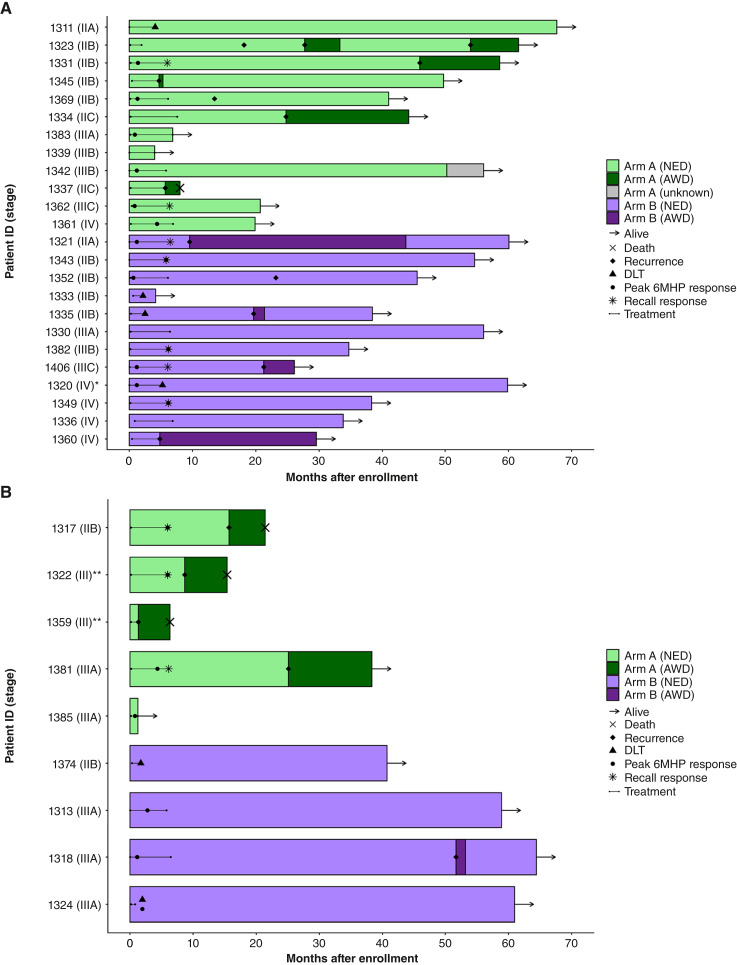
Summary of immunologic and clinical outcomes. Swimmer plots showing immunologic outcomes and the clinical course of patients with (**A**) cutaneous melanoma and disease arising from an unknown primary (*) and (**B**) ocular and mucosal (**) melanomas, grouped by randomized arm and ordered by AJCC stage. Recall response represents those with either a protocol-defined memory response, alternatively defined memory response, or expansion after week 25 booster vaccination. Bars are colored by randomized arm with different shades to reflect disease status; unknown refers to alive with unknown disease status. AWD, alive with disease; NED, no evidence of disease.

A summary of the immunologic outcomes and clinical course for each participant is shown in Fig. 5. Among the 24 participants with cutaneous melanoma or disease arising from an unknown primary site (Fig. 5A), 13 (54%) had at least one CD4^+^ T-cell response. A total of 11 (46%) recurred, with six on arm A with recurrence at a median of 15.8 months (range: 4.7–46 months) and five on arm B with recurrence at a median of 19.6 months (range: 4.8–23.2 months). One (arm A) with stage IIIC disease at enrollment died of advanced melanoma at 8 months. Among the nine participants with either ocular or mucosal melanoma (Fig. 5B), seven (78%) had at least one CD4^+^ T-cell response. A total of five (56%) recurred, with four on arm A with recurrence at a median of 12 months (range: 1.3–25.1 months) and one on arm B with recurrence at 51.7 months. Of these, three died of advanced melanoma, all on arm A, including the two participants with mucosal melanoma and one participant with stage IIB ocular melanoma.

## Discussion

We report the safety, immunologic, and clinical outcomes of a melanoma helper peptide vaccine with or without systemic varlilumab. The combination was well-tolerated and safe but did not augment the immune response. Vaccination induced memory responses at similar rates with or without varlilumab. However, the addition of varlilumab was associated with a significant, sustained reduction in circulating CD4^+^ T cells, without a significant decrease in the relative Treg proportion of the CD4^+^ T-cell compartment, and worse clinical outcomes compared with vaccine alone.

An unexpected safety signal was observed due to local toxicity of the vaccine alone. The types of TRAEs were consistent with those reported in prior trials with 6MHP vaccines in IFA (11, 13, 14, 16), including with poly-ICLC (18), but we observed a greater frequency of severe injection site reactions in this trial. Although prior 6MHP trials have also used half-intradermal/half-subcutaneous administration, most were designed with vaccines either given as split dose between two different extremity sites for an initial priming series (11, 16) or given as full dose at different extremity skin sites for the priming and booster series (13, 18); thus, same-site injection of the full dose for all vaccines plus poly-ICLC in this trial may have contributed to greater local toxicity. The protocol amendment succeeded in mitigating local toxicity. The differences in the safety profile of 6MHP vaccination in this trial compared with prior trials also suggest a benefit of split-dose injections to be considered for future trials. Among the other TRAEs, lymphopenia was more common in those who received varlilumab, as commonly reported (5, 6, 9). No new patterns of toxicity were observed with vaccine plus systemic varlilumab.

Overall, the peripheral CD4^+^ T-cell response rate to 6MHP *ex vivo* was 61%, consistent with prior 6MHP trials (11, 14, 16, 18). Contrary to our hypothesis, addition of systemic varlilumab did not improve durability of the T-cell response, as no notable difference in rates of dRsp, pRsp, and mRsp were found between arms. The overall conclusions remain the same when summarizing the data before and after protocol amendment. CD27 stimulation supports effector CD8^+^ T-cell survival in nonlymphoid tissue via IL2-dependent signaling (20). A preclinical model has shown that CD27 agonism augments vaccine efficacy in the presence of CD4^+^ T-cell help but also promotes CD8^+^ T-cell effector and memory differentiation independent of CD4^+^ T-cell help (7). The 6MHP vaccine only includes known epitopes for CD4^+^ T cells, for which responses may be more dependent on OX40 costimulation than CD27 (21). Thus, improved CD8^+^ T-cell response rates with varlilumab may have been expected with a vaccine that also included epitopes for CD8^+^ T cells; however, this was not observed in a small study of patients with glioma who received varlilumab plus a vaccine targeting both CD8^+^ and CD4^+^ T cells (9). In that study, patients who received vaccine plus varlilumab had significant increases in circulating CD8^+^ and CD4^+^ T cells with an effector memory phenotype at the time of surgical resection compared with before surgery, but expansion after antigen rechallenge in the adjuvant setting was not tested (9). Thus, our study adds another dimension to characterizing immunogenicity by dedicated assessment of memory response after booster vaccination. Although our data do not support enhanced CD4^+^ T-cell memory responses with the addition of varlilumab, 42% and 53% of participants overall had evidence of a vaccine-induced T-cell memory and T-cell expansion after booster vaccination, respectively. Additionally, all participants had durable IgG responses to 6MHP, suggesting T cell–dependent activation of B cells mediated by antigen-specific CD4^+^ T cells. Together, these findings support the promise of cancer vaccines to augment immunotherapeutic approaches for solid tumors.

We found that participants who received varlilumab had significant, sustained decreases in circulating Tregs and CD4^+^ T cells over time from baseline, consistent with prior studies evaluating systemic varlilumab as monotherapy (5) or in combination with anti–PD-1 therapy (6), compared with those that received vaccine alone in this study. However, participants who received varlilumab did not have reduced Tregs as a percentage of CD4^+^ T cells over time or compared with those who received vaccine alone. These findings, in addition to the immune response data, do not suggest a favorable redistribution of the CD4^+^ T-cell compartment with the addition of varlilumab. Participants who received vaccine alone did not have significant changes in the absolute number of circulating Tregs over time, and the percentage of Tregs out of CD4^+^ T cells was stable over time, except for a decrease after booster vaccination, compared with participants who also received varlilumab. These results suggest that helper peptide vaccination does not induce Treg expansion, though further investigation is needed.

This study was not powered for comparison of clinical outcomes by arm; however, there were notable differences in DFS and OS. Participant heterogeneity in primary disease site and stage limit interpretation of these results. Yet, the 4-year DFS rate of 20% (95% CI, 6%–67%) for participants who received varlilumab was lower than expected. For participants who received vaccine alone, the 4-year DFS rate of 69% (95% CI, 49%–96%) compares favorably with our prior trials with 6MHP as adjuvant therapy (14, 18). This difference in DFS between arms suggests that the depletion of CD4^+^ T cells after varlilumab may have reduced or negated the benefit of vaccination, despite an absolute decrease in Tregs. The 4-year OS rate of 100% for vaccine alone is encouraging. Of the six participants on arm B with disease recurrence, five had cutaneous disease who subsequently received ICIs for treatment of their recurrence. Thus, there may be a benefit in inducing tumor-cognate CD4^+^ T cells with helper peptide vaccination prior to ICIs.

The peptides of 6MHP are derived from melanocytic differentiation proteins (MDP), including gp100, tyrosinase, and Melan-A/MART-1, and cancer testis antigens (CTA), specifically the MAGE protein family. These shared antigens are expressed by cutaneous and noncutaneous melanomas. For that reason, participant eligibility was not limited to cutaneous primaries, especially given the limited approved adjuvant therapies available for patients with ocular disease. All patients were without clinical or radiographic evidence of disease at enrollment. Thus, we would not expect that there would be substantial alterations in participants’ systemic immunity by primary disease site affecting their ability to mount a peripheral immune response to vaccine beyond individual-level variation in overall immunologic fitness. Expression of MDPs and CTAs differs by disease stage in cutaneous melanomas, with a shift to less expression of MDPs and greater expression of CTAs in metastatic lesions (22–24). However, prior work has shown that melanoma metastases arising from ocular primaries maintain greater expression of MDPs compared with those arising from cutaneous primaries (25, 26). Thus, it is possible that differences in shared antigen expression in melanoma metastases may have implications for the clinical efficacy of 6MHP vaccination independent of vaccine-induced peripheral immune responses.

Additionally, the trial enrolled patients with definitively treated melanoma at high risk of recurrence across a range of stages, including those with stage IIB to IIC and stage IIA cutaneous and ocular melanoma considered high-risk by genetic expression profiling. At the time that this trial was initiated, anti–PD-1 therapy had not been approved for resected stage IIB to IIC cutaneous melanoma and thus would have not been a standard therapy offered to patients at that time. Nonetheless, this recently approved indication for immunotherapy in these patients supports the rationale for inclusion of patients with early-stage disease at high risk of recurrence in melanoma vaccine trials. This trial excluded patients with metastatic disease who had received ICI therapy within 12 weeks of registration. The objectives of the present study did not support the addition of concurrent ICI therapy into the treatment regimen. The exclusion for recent ICI therapy was due in part to allow for assessment of the safety profile of 6MHP with varlilumab without potential confounding from toxicities of ICI therapies. Furthermore, preclinical models suggest that the optimal timing of the combination of therapeutic cancer vaccination with ICIs is either administering the vaccine prior to ICI therapy or both concurrently (27, 28). It is possible that the benefits of vaccination after initiation of ICI therapy may be diminished due to early immunologic changes induced by ICIs. Thus, recent use of ICI therapy was an exclusion criterion given that it was not an on-study treatment that could be appropriately timed with initiation of vaccine therapy.

Our study has several limitations. This trial was designed to evaluate safety and obtain preliminary estimates of differences in durable immunogenicity by arm; thus, the small sample size limits formal comparison of the immune response rates between arms and interpretation of the clinical outcomes by arm, including the ability to stratify by primary disease site or stage. Although a novel component of this trial was an assessment of functional recall response to characterize T-cell memory, we did not perform flow cytometry or other analyses at a single-cell level to characterize the phenotype of these circulating T cells beyond an assessment for changes in major T-cell subsets over time with a specific interest in the Treg compartment. Although we would expect that patients with T-cell responses at late time points would have functional recall responses to antigen rechallenge, it is possible that sampling heterogeneity or experimental variability may have affected those results. Thus, a consideration for future trial design is to include single-cell assessments to characterize circulating T-cell subsets and concordance with functional assays. Nonetheless, our collective findings from this trial do not support therapeutic potential of varlilumab in the context of melanoma helper peptide vaccination in the adjuvant setting. Additionally, the protocol amendment to reduce local toxicity of the vaccine limited the ability to perform planned analyses of the VSME. However, the vaccine biopsies obtained on this trial remain valuable for characterizing the VSME in future work to compare IFA-based vaccination with other local vaccine adjuvant combinations for 6MHP.

In summary, systemic varlilumab with melanoma helper peptide vaccination was safe but did not enhance durable immunogenicity and was associated with worse clinical outcomes compared with vaccine alone in patients with high-risk melanoma in the adjuvant setting. The induction of memory responses supports further work to optimize shared antigen cancer vaccines in combination with other immunotherapies.

## Supplementary Material

Figure S1 Flow cytometry gating of circulating Tregs

Figure S2 IgG antibody responses to 6MHP

Table S1 Representativeness of study participants

Table S2 Dose-limiting toxicity rates by treatment arm

Table S3 All treatment-related adverse events

Table S4 Treatment-related adverse events prior to protocol amendment

Table S5 Treatment-related adverse events after protocol amendment

Table S6 All adverse events

Table S7 Immune response rates by protocol version

Table S8 Changes in absolute number of circulating Tregs over time

Table S9 Changes in absolute number of circulating CD4+ T cells over time

Table S10 Changes in circulating Tregs as a percentage of CD4+ T cells over time

Supplementary File 1 Supplementary File 1 - Protocol

## Data Availability

Key data are reported in this article and will be maintained on ClinicalTrials.gov. Deidentified participant data are available from the corresponding author on reasonable request.
