# Supplementary material for: Randomized Phase I/II Clinical Trial of a Melanoma Helper Peptide Vaccine with or without Systemic Agonistic Anti-CD27 Antibody (Varlilumab)
Source: Cancer Res Commun. 2026 Apr 30;6(4):994–1005. doi: 10.1158/2767-9764.CRC-25-0744 (PMC13130881; doi:10.1158/2767-9764.CRC-25-0744)
Supplement: Figure S1 — Flow cytometry gating of circulating Tregs [file crc-25-0744_figure_s1_suppsf1.pdf]

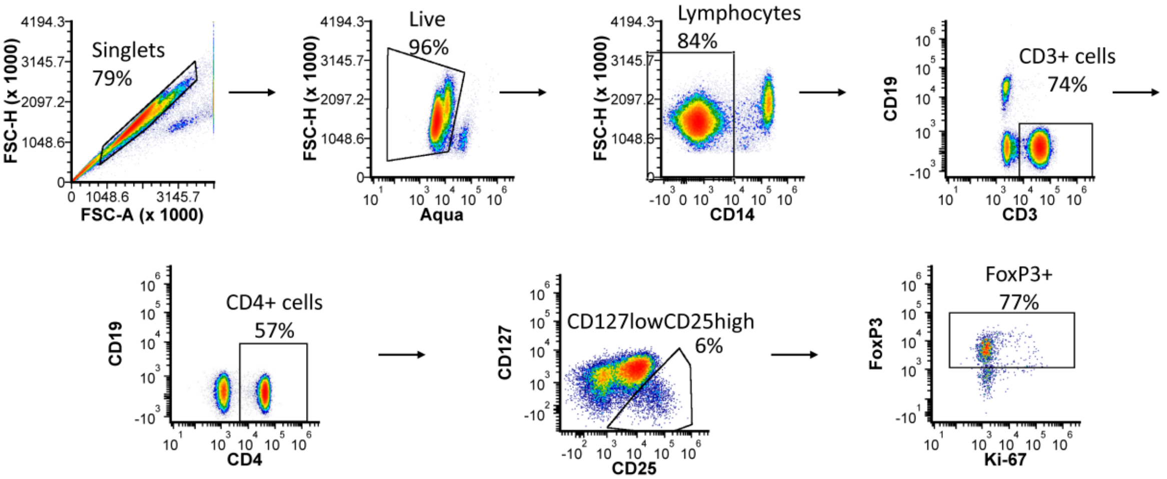

Figure S1. Flow cytometry gating of circulating Tregs. PBMC were evaluated by flow cytometry to identify regulatory T cells ($CD3^{+}CD4^{+}CD25^{hi}CD127^{lo/-}FoxP3^{+}$).
