# Supplementary material for: Randomized Phase I/II Clinical Trial of a Melanoma Helper Peptide Vaccine with or without Systemic Agonistic Anti-CD27 Antibody (Varlilumab)
Source: Cancer Res Commun. 2026 Apr 30;6(4):994–1005. doi: 10.1158/2767-9764.CRC-25-0744 (PMC13130881; doi:10.1158/2767-9764.CRC-25-0744)
Supplement: Figure S2 — IgG antibody responses to 6MHP [file crc-25-0744_figure_s2_suppsf2.pdf]

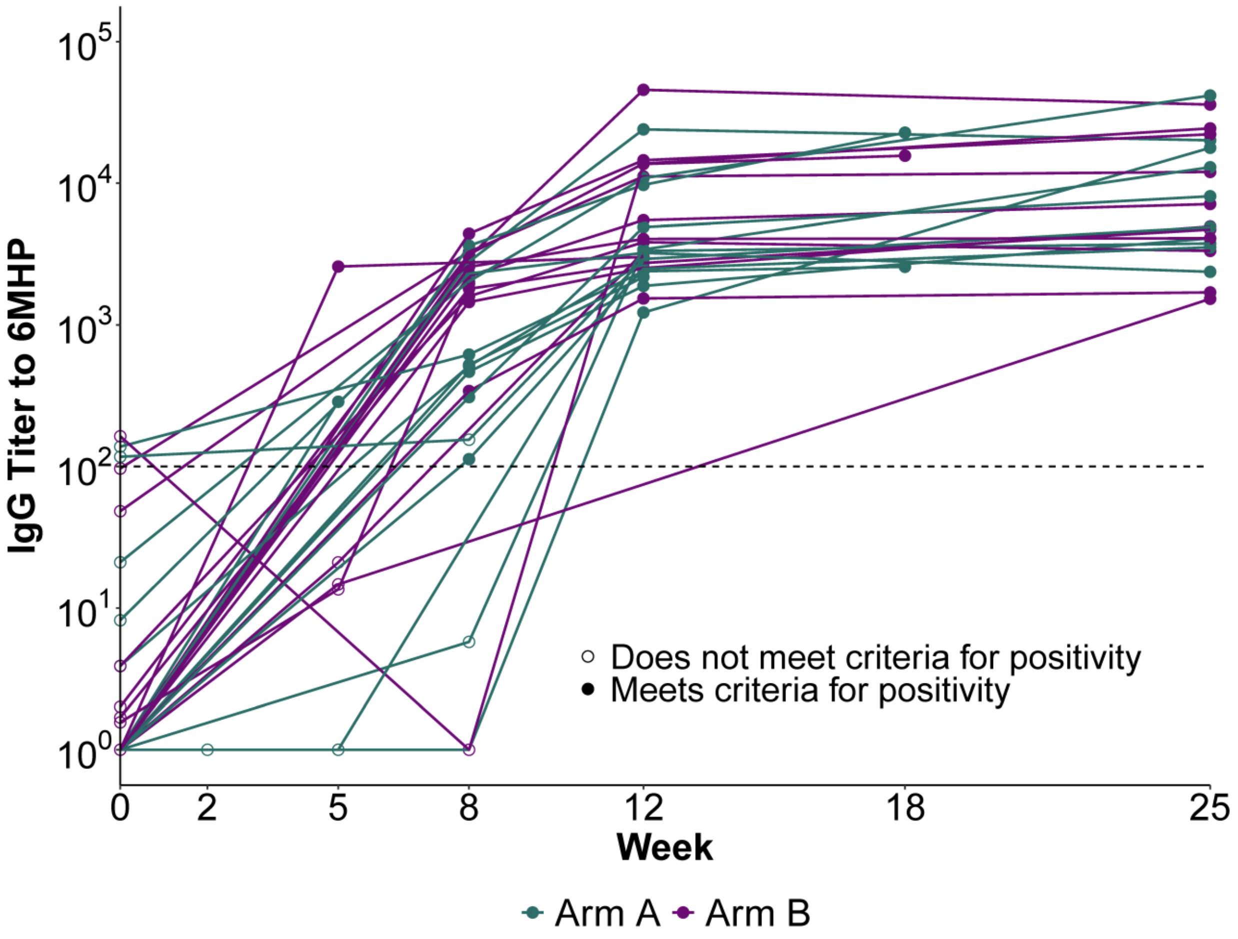

Figure S2. IgG antibody responses to 6MHP. Participants with serum samples (n=31) were evaluated by ELISA. One participant on Arm A was missing a week 0 sample, and thus the week 2 sample was used as baseline. Plots show estimated IgG titer to 6MHP by treatment arm on log10 scale, with responses requiring a minimum titer of 100 (dashed line) and at least four-fold greater than negative control and any baseline reactivity.
