# Supplementary material for: Randomized Phase I/II Clinical Trial of a Melanoma Helper Peptide Vaccine with or without Systemic Agonistic Anti-CD27 Antibody (Varlilumab)
Source: Cancer Res Commun. 2026 Apr 30;6(4):994–1005. doi: 10.1158/2767-9764.CRC-25-0744 (PMC13130881; doi:10.1158/2767-9764.CRC-25-0744)
Supplement: Table S1 — Representativeness of study participants [file crc-25-0744_table_s1_suppst1.pdf]

**Cancer Type**	Cutaneous Melanoma	Unknown Primary Melanoma	Mucosal Melanoma	Ocular Melanoma
**Considerations related to[1-5]:**				
Sex:	More common in males (M:F ratio 3:2)	More common in males (M:F ratio 2:1)	More common in females (M:F ratio 0.6:1)	More common in males (M:F ratio 1.1:1)
Age	Median age at diagnosis: 66 years, with about 27% diagnosed between 65-74 years.	Median age at diagnosis: 50-60 years, with about 45% over age 60.	Median age at diagnosis about 70 years, with about 72% over age 60.	Median age at diagnosis about 50-60 years, with about 57% over age 60.
Race/ethnicity	98% non-Hispanic Whites. In the U.S., about 0.9% are African-Americans and about 1.3% are Hispanic.	95% non-Hispanic White	86% non-Hispanic White	92% non-Hispanic White
Geography	5th most commonly diagnosed cancer in the United States, with an estimated 100,640 new cases in 2024 (5% of all new cancer cases).	About 3.2% of all melanoma diagnoses.	About 2.2 patients per million per year in the United States	About 5.5 patients per million per year in the United States.
**Other considerations**	This trial included patients with cutaneous melanoma and one with an unknown primary, which is commonly considered similar to a cutaneous melanoma. It also included patients with rarer melanomas arising from ocular or mucosal primary sites.			
**Overall representativeness of this study**	The age distribution of patients in this study is similar to those reported by age group for cutaneous melanoma. There was approximately equal representation of males to females enrolled in this trial. All trial participants were non-Hispanic ethnicity, and all were White except for one African-American. These demographics reflect what would be expected for the sample size (n=33) of this trial based on incidence rates in the United States by race/ethnicity (expected enrollment of 0-1 patients within minority racial/ethnic groups). There were no exclusion criteria based on race/ethnicity in this trial.			

Table S1. Representativeness of study participants.

[1] SEER*Explorer: An interactive website for SEER cancer statistics [Internet]. Surveillance Research Program, National Cancer Institute; 2025 Jul 2. [cited 2025 Nov 19]. Available from: https://seer.cancer.gov/statistics-network/explorer/. Data source(s): SEER Incidence Data, November 2024 Submission (1975-2022), SEER 21 registries.

[2] Chang AE, Karnell LH, Menck HR The National Cancer Data Base Report on Cutaneous and Noncutaneous Melanoma: A Summary of 84,836 Cases from the Past decade. Cancer 1998; 83(8): 1664-1678.

[3] Alfaar AS, Abdel-Rahman MH Osman MH. Longitudinal nationwide analysis of uveal melanoma in the United States 1995-2018, Intl J of Clinical Oncology (2025) 30:2375-2386.

[4] Broit N, Johansson PA, Rodgers CB, Walpole ST, Newell F; Nicholas K. Hayward NK, Pritchard AL. Meta-Analysis and Systematic Review of the Genomics of Mucosal Melanoma. Mol Cancer Res (2021) 19 (6): 991–1004.

[5] Kamposioras K, Pentheroudakis G, Pectasides D, Pavlidis N. Malignant melanoma of unknown primary site. To make the long story short. A systematic review of the literature. Crit Rev Oncol Hematol. 2011;78(2):112–26.
