# Supplementary material for: Randomized Phase I/II Clinical Trial of a Melanoma Helper Peptide Vaccine with or without Systemic Agonistic Anti-CD27 Antibody (Varlilumab)
Source: Cancer Res Commun. 2026 Apr 30;6(4):994–1005. doi: 10.1158/2767-9764.CRC-25-0744 (PMC13130881; doi:10.1158/2767-9764.CRC-25-0744)
Supplement: Table S2 — Dose-limiting toxicity rates by treatment arm [file crc-25-0744_table_s2_suppst2.pdf]

	Arm A	Arm B	Total
**Before protocol amendment**	1/8 (13%)	4/9 (44%)	5/17 (29%)
**After protocol amendment**	0/9 (0%)	1/7 (14%)	1/16 (6%)
**Total**	1/17 (6%)	5/16 (31%)	6/33 (18%)

Table S2. Dose-limiting toxicity rates by treatment arm. Rates of dose-limiting toxicities (DLTs) by treatment arm before and after major protocol amendment on December 3, 2020 to reduce vaccine site toxicity.
