# Supplementary material for: Randomized Phase I/II Clinical Trial of a Melanoma Helper Peptide Vaccine with or without Systemic Agonistic Anti-CD27 Antibody (Varlilumab)
Source: Cancer Res Commun. 2026 Apr 30;6(4):994–1005. doi: 10.1158/2767-9764.CRC-25-0744 (PMC13130881; doi:10.1158/2767-9764.CRC-25-0744)
Supplement: Table S4 — Treatment-related adverse events prior to protocol amendment [file crc-25-0744_table_s4_suppst4.pdf]

| | | Arm A (N=8) | | | | Arm B (N=9) | | | | Total (N=17) | | | |
|---|---|---|---|---|---|---|---|---|---|---|---|---|---|
| **Category** | **TRAE, n (%)** | **G1** | **G2** | **G3** | **Total** | **G1** | **G2** | **G3** | **Total** | **G1** | **G2** | **G3** | **Total** |
| **Maximum, n (%)** | | **2 (25)** | **3 (38)** | **3 (38)** | **8 (100)** | **2 (22)** | **3 (33)** | **4 (44)** | **9 (100)** | **4 (24)** | **6 (35)** | **7 (41)** | **17 (100)** |
| BLOOD/LYMPHATIC | ANEMIA | 1 (13) | | | 1 (13) | | | | | 1 (6) | | | 1 (6) |
| GASTROINTESTINAL | DIARRHEA | 1 (13) | 1 (13) | | 2 (25) | 1 (11) | | | 1 (11) | 2 (12) | 1 (6) | | 3 (18) |
| | NAUSEA | 1 (13) | | | 1 (13) | 1 (11) | 1 (11) | | 2 (22) | 2 (12) | 1 (6) | | 3 (18) |
| | ORAL PAIN | | | | | 1 (11) | | | 1 (11) | 1 (6) | | | 1 (6) |
| | VOMITING | | | | | 1 (11) | | | 1 (11) | 1 (6) | | | 1 (6) |
| GENERAL AND ADMINISTRATION SITE | CHILLS | 2 (25) | | | 2 (25) | 1 (11) | | | 1 (11) | 3 (18) | | | 3 (18) |
| | FATIGUE | 4 (50) | | | 4 (50) | 4 (44) | 1 (11) | | 5 (56) | 8 (47) | 1 (6) | | 9 (53) |
| | FEVER | | | | | 3 (33) | | | 3 (33) | 3 (18) | | | 3 (18) |
| | FLU LIKE SYMPTOMS | 2 (25) | | | 2 (25) | 4 (44) | | | 4 (44) | 6 (35) | | | 6 (35) |
| | INJECTION SITE REACTION | 4 (50) | 3 (38) | 1 (13) | 8 (100) | 5 (56) | 3 (33) | | 8 (89) | 9 (53) | 6 (35) | 1 (6) | 16 (94) |
| | MALAISE | | | | | 1 (11) | | | 1 (11) | 1 (6) | | | 1 (6) |
| | PAIN | | 1 (13) | | 1 (13) | 1 (11) | 1 (11) | | 2 (22) | 1 (6) | 2 (12) | | 3 (18) |
| IMMUNE SYSTEM | ALLERGIC REACTION | | | | | | 1 (11) | | 1 (11) | | 1 (6) | | 1 (6) |
| INVESTIGATIONS | LYMPHOCYTE COUNT DECREASED | 1 (13) | 2 (25) | 1 (13) | 4 (50) | 1 (11) | | 1 (11) | 2 (22) | 2 (12) | 2 (12) | 2 (12) | 6 (35) |
| | NEUTROPHIL COUNT DECREASED | 1 (13) | | | 1 (13) | | 1 (11) | | 1 (11) | 1 (6) | 1 (6) | | 2 (12) |
| | THYROID STIMULATING HORMONE INCREASED | 1 (13) | | | 1 (13) | | | | | 1 (6) | | | 1 (6) |
| | WHITE BLOOD CELL DECREASED | | | | | | 1 (11) | | 1 (11) | | 1 (6) | | 1 (6) |
| METABOLISM/NUTRITION | ANOREXIA | | | | | 2 (22) | 1 (11) | | 3 (33) | 2 (12) | 1 (6) | | 3 (18) |
| MUSCULOSKELETAL/CONNECTIVE TISSUE | ARTHRALGIA | 2 (25) | | | 2 (25) | 2 (22) | | | 2 (22) | 4 (24) | | | 4 (24) |
| | MYALGIA | 1 (13) | | | 1 (13) | 4 (44) | | | 4 (44) | 5 (29) | | | 5 (29) |
| NERVOUS SYSTEM | DIZZINESS | 1 (13) | | | 1 (13) | | | | | 1 (6) | | | 1 (6) |
| | HEADACHE | 2 (25) | | | 2 (25) | 2 (22) | | | 2 (22) | 4 (24) | | | 4 (24) |
| RESPIRATORY/THORACIC/MEDIASTINAL | COUGH | 1 (13) | | | 1 (13) | 1 (11) | | | 1 (11) | 2 (12) | | | 2 (12) |
| | NASAL CONGESTION | 1 (13) | | | 1 (13) | | | | | 1 (6) | | | 1 (6) |
| | PNEUMONITIS | | | | | 1 (11) | 1 (11) | | 2 (22) | 1 (6) | 1 (6) | | 2 (12) |
| | SORE THROAT | 1 (13) | | | 1 (13) | 1 (11) | | | 1 (11) | 2 (12) | | | 2 (12) |
| SKIN/SUBCUTANEOUS TISSUE | DRY SKIN | 1 (13) | | | 1 (13) | 1 (11) | | | 1 (11) | 2 (12) | | | 2 (12) |
| | HYPERHIDROSIS | 1 (13) | | | 1 (13) | 1 (11) | | | 1 (11) | 2 (12) | | | 2 (12) |
| | OTHER: weeping/exudate | | | | | 1 (11) | | | 1 (11) | 1 (6) | | | 1 (6) |
| | PRURITUS | | | | | 1 (11) | | | 1 (11) | 1 (6) | | | 1 (6) |
| | SKIN INDURATION | 2 (25) | 5 (63) | | 7 (88) | 4 (44) | 3 (33) | | 7 (78) | 6 (35) | 8 (47) | | 14 (82) |
| | SKIN ULCERATION | 1 (13) | | 1 (13) | 2 (25) | 1 (11) | 1 (11) | 3 (33) | 5 (56) | 2 (12) | 1 (6) | 4 (24) | 7 (41) |
| VASCULAR | FLUSHING | | | | | 1 (11) | | | 1 (11) | 1 (6) | | | 1 (6) |

**Table S4. Treatment-related adverse events prior to protocol amendment.** The number of participants that experienced each treatment-related adverse event (TRAE) is shown by grade (G1-G3) for each treatment arm prior to major protocol amendment on December 3, 2020. No grade 4 or 5 TRAEs were observed. Numbers in parenthesis represent the percentage of participants reporting the TRAE of that grade for each treatment arm. The summary row labeled Maximum refers to the total number of participants reporting any TRAE of that grade for each treatment arm.
