# Supplementary material for: Randomized Phase I/II Clinical Trial of a Melanoma Helper Peptide Vaccine with or without Systemic Agonistic Anti-CD27 Antibody (Varlilumab)
Source: Cancer Res Commun. 2026 Apr 30;6(4):994–1005. doi: 10.1158/2767-9764.CRC-25-0744 (PMC13130881; doi:10.1158/2767-9764.CRC-25-0744)
Supplement: Table S5 — Treatment-related adverse events after protocol amendment [file crc-25-0744_table_s5_suppst5.pdf]

		Arm A (N=9)				Arm B (N=7)				Total (N=16)			
**Category**	**TRAE, n (%)**	**G1**	**G2**	**G3**	**Total**	**G1**	**G2**	**G3**	**Total**	**G1**	**G2**	**G3**	**Total**
**Maximum, n (%)**		**3 (33)**	**5 (56)**	**1 (11)**	**9 (100)**	**5 (71)**	**1 (14)**	**1 (14)**	**7 (100)**	**8 (50)**	**6 (38)**	**2 (13)**	**16 (100)**
BLOOD/LYMPHATIC	ANEMIA	1 (11)			1 (11)					1 (6)			1 (6)
	EOSINOPHILIA					1 (14)			1 (14)	1 (6)			1 (6)
EAR/LABYRINTH	OTHER: Ear Pressure	1 (11)			1 (11)					1 (6)			1 (6)
EYE	BLURRED VISION							1 (14)	1 (14)			1 (6)	1 (6)
	RETINAL DETACHMENT							1 (14)	1 (14)			1 (6)	1 (6)
GASTROINTESTINAL	CONSTIPATION	1 (11)			1 (11)					1 (6)			1 (6)
	DIARRHEA	1 (11)			1 (11)					1 (6)			1 (6)
	NAUSEA	1 (11)			1 (11)					1 (6)			1 (6)
GENERAL AND ADMINISTRATION SITE	CHILLS	1 (11)			1 (11)	3 (43)			3 (43)	4 (25)			4 (25)
	EDEMA LIMBS	1 (11)			1 (11)					1 (6)			1 (6)
	FATIGUE	3 (33)			3 (33)	3 (43)			3 (43)	6 (38)			6 (38)
	FEVER	1 (11)			1 (11)	1 (14)	1 (14)		2 (29)	2 (13)	1 (6)		3 (19)
	FLU LIKE SYMPTOMS	1 (11)			1 (11)	2 (29)			2 (29)	3 (19)			3 (19)
	INJECTION SITE REACTION	8 (89)			8 (89)	6 (86)	1 (14)		7 (100)	14 (88)	1 (6)		15 (94)
	LOCALIZED EDEMA	1 (11)			1 (11)					1 (6)			1 (6)
	OTHER: Swelling Legs	1 (11)			1 (11)					1 (6)			1 (6)
	PAIN	1 (11)			1 (11)					1 (6)			1 (6)
INJURY/POISONING/PROCEDURAL	INFUSION RELATED REACTION	1 (11)			1 (11)					1 (6)			1 (6)
INVESTIGATIONS	LYMPHOCYTE COUNT DECREASED		5 (56)		5 (56)	1 (14)			1 (14)	1 (6)	5 (31)		6 (38)
	NEUTROPHIL COUNT DECREASED	1 (11)			1 (11)	1 (14)			1 (14)	2 (13)			2 (13)
	THYROID STIMULATING HORMONE INCREASED	1 (11)			1 (11)					1 (6)			1 (6)
	WHITE BLOOD CELL DECREASED			1 (11)	1 (11)	1 (14)			1 (14)	1 (6)		1 (6)	2 (13)
METABOLISM/NUTRITION	HYPERKALEMIA					1 (14)			1 (14)	1 (6)			1 (6)
	HYPOKALEMIA	1 (11)			1 (11)					1 (6)			1 (6)
	HYPONATREMIA					1 (14)			1 (14)	1 (6)			1 (6)
MUSCULOSKELETAL/CONNECTIVE TISSUE	MYALGIA	2 (22)			2 (22)	1 (14)			1 (14)	3 (19)			3 (19)
	PAIN IN EXTREMITY	1 (11)			1 (11)					1 (6)			1 (6)
NERVOUS SYSTEM	DIZZINESS	1 (11)			1 (11)					1 (6)			1 (6)
	HEADACHE	2 (22)			2 (22)	1 (14)			1 (14)	3 (19)			3 (19)
RESPIRATORY/THORACIC/MEDIASTINAL	ALLERGIC RHINITIS					1 (14)			1 (14)	1 (6)			1 (6)
	COUGH	1 (11)			1 (11)					1 (6)			1 (6)
SKIN/SUBCUTANEOUS TISSUE	DRY SKIN	1 (11)			1 (11)	1 (14)			1 (14)	2 (13)			2 (13)
	ERYTHEMA MULTIFORME	2 (22)			2 (22)	1 (14)			1 (14)	3 (19)			3 (19)
	PRURITUS	2 (22)			2 (22)					2 (13)			2 (13)
	RASH MACULO-PAPULAR	2 (22)			2 (22)	1 (14)			1 (14)	3 (19)			3 (19)
	SKIN HYPERPIGMENTATION	1 (11)			1 (11)					1 (6)			1 (6)
	SKIN INDURATION	1 (11)			1 (11)	1 (14)			1 (14)	2 (13)			2 (13)
	SKIN ULCERATION		1 (11)		1 (11)						1 (6)		1 (6)
VASCULAR	FLUSHING	2 (22)			2 (22)					2 (13)			2 (13)
	HOT FLASHES					1 (14)			1 (14)	1 (6)			1 (6)

Table S5. Treatment-related adverse events after protocol amendment. The number of participants that experienced each treatment-related adverse event (TRAE) is shown by grade (G1-G3) for each treatment arm after major protocol amendment on December 3, 2020. No grade 4 or 5 TRAEs were observed. Numbers in parenthesis represent the percentage of participants reporting the TRAE of that grade for each treatment arm. The summary row labeled Maximum refers to the total number of participants reporting any TRAE of that grade for each treatment arm.
