# Supplementary material for: Randomized Phase I/II Clinical Trial of a Melanoma Helper Peptide Vaccine with or without Systemic Agonistic Anti-CD27 Antibody (Varlilumab)
Source: Cancer Res Commun. 2026 Apr 30;6(4):994–1005. doi: 10.1158/2767-9764.CRC-25-0744 (PMC13130881; doi:10.1158/2767-9764.CRC-25-0744)
Supplement: Table S6 — All adverse events [file crc-25-0744_table_s6_suppst6.pdf]

Category	AE, n (%)	Arm A (N=17)				Arm B (N=16)				Total (N=33)			
		G1	G2	G3	Total	G1	G2	G3	Total	G1	G2	G3	Total
**Maximum, n (%)**		**2 (12)**	**8 (47)**	**7 (41)**	**17 (100)**	**2 (13)**	**6 (38)**	**8 (50)**	**16 (100)**	**4 (12)**	**14 (42)**	**15 (45)**	**33 (100)**
BLOOD/LYMPHATIC	ANEMIA	4 (24)	1 (6)		5 (29)	5 (31)	2 (13)		7 (44)	9 (27)	3 (9)		12 (36)
	EOSINOPHILIA					1 (6)			1 (6)	1 (3)			1 (3)
CARDIAC	SINUS BRADYCARDIA	6 (35)			6 (35)	4 (25)			4 (25)	10 (30)			10 (30)
	SINUS TACHYCARDIA	1 (6)			1 (6)					1 (3)			1 (3)
EAR/LABYRINTH	EAR PAIN	1 (6)			1 (6)					1 (3)			1 (3)
	OTHER: Ear Pressure	1 (6)			1 (6)					1 (3)			1 (3)
	TINNITUS	1 (6)			1 (6)					1 (3)			1 (3)
	VERTIGO					1 (6)			1 (6)	1 (3)			1 (3)
EYE	BLURRED VISION					1 (6)		1 (6)	2 (13)	1 (3)		1 (3)	2 (6)
	CATARACT						1 (6)		1 (6)		1 (3)		1 (3)
	DRY EYE	1 (6)			1 (6)					1 (3)			1 (3)
	OTHER: erythema right eye	1 (6)			1 (6)					1 (3)			1 (3)
	RETINAL DETACHMENT							1 (6)	1 (6)			1 (3)	1 (3)
	WATERING EYES	2 (12)			2 (12)					2 (6)			2 (6)
GASTROINTESTINAL	ABDOMINAL PAIN	1 (6)			1 (6)	1 (6)			1 (6)	2 (6)			2 (6)
	BLOATING		1 (6)		1 (6)						1 (3)		1 (3)
	CONSTIPATION	2 (12)			2 (12)	2 (13)			2 (13)	4 (12)			4 (12)
	DIARRHEA	4 (24)	1 (6)		5 (29)	2 (13)			2 (13)	6 (18)	1 (3)		7 (21)
	DRY MOUTH	1 (6)			1 (6)					1 (3)			1 (3)
	ENTEROCOLITIS		1 (6)		1 (6)						1 (3)		1 (3)
	GASTROESOPHAGEAL REFLUX DISEASE	1 (6)			1 (6)					1 (3)			1 (3)
	MUCOSITIS ORAL	3 (18)			3 (18)	1 (6)			1 (6)	4 (12)			4 (12)
	NAUSEA	2 (12)	1 (6)		3 (18)	1 (6)	1 (6)		2 (13)	3 (9)	2 (6)		5 (15)
	ORAL PAIN					1 (6)			1 (6)	1 (3)			1 (3)
	VOMITING	3 (18)			3 (18)	1 (6)			1 (6)	4 (12)			4 (12)
GENERAL AND ADMINISTRATION SITE	CHILLS	4 (24)			4 (24)	5 (31)			5 (31)	9 (27)			9 (27)
	EDEMA LIMBS	2 (12)			2 (12)		1 (6)		1 (6)	2 (6)	1 (3)		3 (9)
	FATIGUE	8 (47)			8 (47)	8 (50)	1 (6)		9 (56)	16 (48)	1 (3)		17 (52)
	FEVER	1 (6)			1 (6)	4 (25)	1 (6)		5 (31)	5 (15)	1 (3)		6 (18)
	FLU LIKE SYMPTOMS	3 (18)			3 (18)	6 (38)			6 (38)	9 (27)			9 (27)
	INJECTION SITE REACTION	12 (71)	3 (18)	1 (6)	16 (94)	11 (69)	4 (25)		15 (94)	23 (70)	7 (21)	1 (3)	31 (94)
	LOCALIZED EDEMA	2 (12)			2 (12)					2 (6)			2 (6)
	MALAISE					1 (6)			1 (6)	1 (3)			1 (3)
	OTHER: Swelling Legs	1 (6)			1 (6)					1 (3)			1 (3)
	PAIN	4 (24)	1 (6)		5 (29)	2 (13)	1 (6)		3 (19)	6 (18)	2 (6)		8 (24)
IMMUNE SYSTEM	ALLERGIC REACTION						1 (6)		1 (6)		1 (3)		1 (3)
INFECTIONS/INFESTATIONS	EYE INFECTION		1 (6)		1 (6)						1 (3)		1 (3)
	FOLLICULITIS					1 (6)			1 (6)	1 (3)			1 (3)
	OTHER: ear infection		1 (6)		1 (6)						1 (3)		1 (3)
	TOOTH INFECTION						1 (6)		1 (6)		1 (3)		1 (3)
	UPPER RESPIRATORY INFECTION	1 (6)			1 (6)	2 (13)	1 (6)		3 (19)	3 (9)	1 (3)		4 (12)
	URINARY TRACT INFECTION						1 (6)		1 (6)		1 (3)		1 (3)
INJURY/POISONING/PROCEDURAL	BRUISING	1 (6)			1 (6)					1 (3)			1 (3)
	FALL	1 (6)			1 (6)	1 (6)			1 (6)	2 (6)			2 (6)
	FRACTURE						1 (6)		1 (6)		1 (3)		1 (3)
	INFUSION RELATED REACTION	1 (6)			1 (6)					1 (3)			1 (3)
	OTHER: discomfort at prior surgical site	1 (6)			1 (6)					1 (3)			1 (3)
	OTHER: red papules	1 (6)			1 (6)					1 (3)			1 (3)
	OTHER: tick bite	1 (6)			1 (6)					1 (3)			1 (3)
	OTHER: Insect bite	1 (6)			1 (6)					1 (3)			1 (3)
INVESTIGATIONS	ALANINE AMINOTRANSFERASE INCREASED	1 (6)			1 (6)					1 (3)			1 (3)
	ASPARTATE AMINOTRANSFERASE INCREASED					4 (25)			4 (25)	4 (12)			4 (12)
	BLOOD BICARBONATE DECREASED					1 (6)			1 (6)	1 (3)			1 (3)
	BLOOD LACTATE DEHYDROGENASE INCREASED	1 (6)			1 (6)					1 (3)			1 (3)
	CREATININE INCREASED					1 (6)			1 (6)	1 (3)			1 (3)
	LYMPHOCYTE COUNT DECREASED	1 (6)	9 (53)	1 (6)	11 (65)	3 (19)		1 (6)	4 (25)	4 (12)	9 (27)	2 (6)	15 (45)
	NEUTROPHIL COUNT DECREASED	2 (12)			2 (12)	1 (6)	1 (6)		2 (13)	3 (9)	1 (3)		4 (12)
	THYROID STIMULATING HORMONE INCREASED	2 (12)			2 (12)					2 (6)			2 (6)
	WHITE BLOOD CELL DECREASED	1 (6)		1 (6)	2 (12)	2 (13)	1 (6)		3 (19)	3 (9)	1 (3)	1 (3)	5 (15)
METABOLISM/NUTRITION	ANOREXIA	3 (18)			3 (18)	3 (19)	1 (6)		4 (25)	6 (18)	1 (3)		7 (21)
	DEHYDRATION		1 (6)		1 (6)						1 (3)		1 (3)
	HYPERGLYCEMIA	1 (6)			1 (6)	3 (19)			3 (19)	4 (12)			4 (12)
	HYPERKALEMIA	1 (6)			1 (6)	2 (13)	1 (6)		3 (19)	3 (9)	1 (3)		4 (12)
	HYPOCALCEMIA					2 (13)			2 (13)	2 (6)			2 (6)
	HYPOGLYCEMIA	1 (6)	1 (6)		2 (12)	3 (19)			3 (19)	4 (12)	1 (3)		5 (15)
	HYPOKALEMIA	1 (6)			1 (6)	1 (6)		1 (6)	2 (13)	2 (6)		1 (3)	3 (9)
	HYPONATREMIA	4 (24)	1 (6)		5 (29)	3 (19)			3 (19)	7 (21)	1 (3)		8 (24)
MUSCULOSKELETAL/CONNECTIVE TISSUE	ARTHRALGIA	2 (12)			2 (12)	3 (19)			3 (19)	5 (15)			5 (15)
	JOINT EFFUSION		1 (6)		1 (6)						1 (3)		1 (3)
	MUSCLE WEAKNESS LOWER LIMB	1 (6)			1 (6)					1 (3)			1 (3)
	MYALGIA	3 (18)			3 (18)	5 (31)			5 (31)	8 (24)			8 (24)
	OTHER: Bilateral axillae soreness	1 (6)			1 (6)					1 (3)			1 (3)
	PAIN IN EXTREMITY	1 (6)			1 (6)					1 (3)			1 (3)
NERVOUS SYSTEM	DIZZINESS	2 (12)		1 (6)	3 (18)	1 (6)			1 (6)	3 (9)		1 (3)	4 (12)
	HEADACHE	4 (24)			4 (24)	4 (25)			4 (25)	8 (24)			8 (24)
	OTHER: Taste Alterations	1 (6)			1 (6)					1 (3)			1 (3)
	PARESTHESIA	1 (6)			1 (6)	1 (6)			1 (6)	2 (6)			2 (6)
PSYCHIATRIC	ANXIETY	1 (6)			1 (6)	1 (6)			1 (6)	2 (6)			2 (6)
	DEPRESSION	1 (6)			1 (6)	1 (6)			1 (6)	2 (6)			2 (6)
RENAL/URINARY	DYSURIA					1 (6)			1 (6)	1 (3)			1 (3)
	GLUCOSURIA	1 (6)			1 (6)	1 (6)			1 (6)	2 (6)			2 (6)
	HEMATURIA					2 (13)			2 (13)	2 (6)			2 (6)
	PROTEINURIA	2 (12)			2 (12)	1 (6)			1 (6)	3 (9)			3 (9)
	URINARY FREQUENCY	1 (6)			1 (6)	1 (6)			1 (6)	2 (6)			2 (6)
	URINARY TRACT PAIN					1 (6)			1 (6)	1 (3)			1 (3)
	URINARY URGENCY					1 (6)			1 (6)	1 (3)			1 (3)
RESPIRATORY/THORACIC/MEDIASTINAL	ALLERGIC RHINITIS	1 (6)	1 (6)		2 (12)	1 (6)			1 (6)	2 (6)	1 (3)		3 (9)
	COUGH	3 (18)			3 (18)	3 (19)			3 (19)	6 (18)			6 (18)
	DYSPNEA					1 (6)			1 (6)	1 (3)			1 (3)
	NASAL CONGESTION	2 (12)			2 (12)	1 (6)			1 (6)	3 (9)			3 (9)
	PNEUMONITIS					1 (6)	1 (6)		2 (13)	1 (3)	1 (3)		2 (6)
	SNEEZING	1 (6)			1 (6)					1 (3)			1 (3)
	SORE THROAT	3 (18)			3 (18)	1 (6)			1 (6)	4 (12)			4 (12)

		Arm A (N=17)				Arm B (N=16)				Total (N=33)			
**Category**	**AE, n (%)**	**G1**	**G2**	**G3**	**Total**	**G1**	**G2**	**G3**	**Total**	**G1**	**G2**	**G3**	**Total**
SKIN/SUBCUTANEOUS TISSUE	ALOPECIA	2 (12)			2 (12)					2 (6)			2 (6)
	DRY SKIN	3 (18)			3 (18)	2 (13)			2 (13)	5 (15)			5 (15)
	ERYTHEMA MULTIFORME	2 (12)			2 (12)	1 (6)			1 (6)	3 (9)			3 (9)
	HYPERHIDROSIS	1 (6)			1 (6)	1 (6)			1 (6)	2 (6)			2 (6)
	OTHER: contact dermatitis	1 (6)			1 (6)					1 (3)			1 (3)
	OTHER: weeping/exudate					1 (6)			1 (6)	1 (3)			1 (3)
	OTHER: spider bite papule	1 (6)			1 (6)					1 (3)			1 (3)
	OTHER: Heat Rash	1 (6)			1 (6)					1 (3)			1 (3)
	OTHER: Dermatology skin tag removal	1 (6)			1 (6)					1 (3)			1 (3)
	OTHER: laceration	1 (6)			1 (6)					1 (3)			1 (3)
	PRURITUS	2 (12)			2 (12)	1 (6)			1 (6)	3 (9)			3 (9)
	RASH MACULO-PAPULAR	2 (12)			2 (12)	3 (19)			3 (19)	5 (15)			5 (15)
	SKIN HYPERPIGMENTATION	1 (6)			1 (6)					1 (3)			1 (3)
	SKIN INDURATION	3 (18)	5 (29)		8 (47)	5 (31)	3 (19)		8 (50)	8 (24)	8 (24)		16 (48)
	SKIN ULCERATION	1 (6)	1 (6)	1 (6)	3 (18)	1 (6)	1 (6)	3 (19)	5 (31)	2 (6)	2 (6)	4 (12)	8 (24)
VASCULAR	FLUSHING	2 (12)			2 (12)	1 (6)			1 (6)	3 (9)			3 (9)
	HOT FLASHES	1 (6)			1 (6)	1 (6)			1 (6)	2 (6)			2 (6)
	HYPERTENSION		2 (12)	3 (18)	5 (29)		4 (25)	5 (31)	9 (56)		6 (18)	8 (24)	14 (42)
	HYPOTENSION	1 (6)			1 (6)					1 (3)			1 (3)

Table S6. All adverse events. The number of participants that experienced each adverse event (AE) is shown by grade (G1-G3) for each treatment arm. No grade 4 or 5 AEs were observed. Numbers in parenthesis represent the percentage of participants reporting the AE of that grade for each treatment arm. The summary row labeled Maximum refers to the total number of participants reporting any AE of that grade for each treatment arm.
