# Supplementary material for: Randomized Phase I/II Clinical Trial of a Melanoma Helper Peptide Vaccine with or without Systemic Agonistic Anti-CD27 Antibody (Varlilumab)
Source: Cancer Res Commun. 2026 Apr 30;6(4):994–1005. doi: 10.1158/2767-9764.CRC-25-0744 (PMC13130881; doi:10.1158/2767-9764.CRC-25-0744)
Supplement: Table S7 — Immune response rates by protocol version [file crc-25-0744_table_s7_suppst7.pdf]

	Any Response	Durable Response	Persistent Response[a]	Early + Late Response	Memory Response		Expansion at Week 26
**Population**	Protocol-defined	Protocol-defined	Protocol-defined	Evaluable	Protocol-defined[b]	Evaluable	Evaluable
	**n/N (%)**	**n/N (%)**	**n/N (%)**	**n/N (%)**	**n/N (%)**	**n/N (%)**	**n/N (%)**
**Before Amendment**							
**Arm A**	4/8 (50)	1/8 (13)	0/6 (0)	2/6 (33)	1/6 (17)	2/5 (40)	3/5 (60)
**Arm B**	6/9 (67)	1/9 (11)	0/9 (0)	1/6 (17)	1/9 (11)	1/5 (20)	2/5 (40)
**Total**	10/17 (59)	2/17 (12)	0/15 (0)	3/12 (25)	2/15 (13)	3/10 (30)	5/10 (50)
**RD (A-B) (90% CI)**	-17% (-52 to +23%)	1% (-29 to +33%)	0% (-24 to +33%)	17% (-27 to +55%)	6% (-27 to +43%)	20% (-30 to +62%)	+20% (-32 to 63%)
**After Amendment**							
**Arm A**	6/9 (67)	2/9 (22)	2/8 (25)	2/7 (29)	1/7 (14)	2/4 (50)	2/4 (50)
**Arm B**	4/7 (57)	3/7 (43)	1/7 (14)	3/6 (50)	3/7 (43)	3/5 (60)	3/5 (60)
**Total**	10/16 (63)	5/16 (31)	3/15 (20)	5/13 (38)	4/14 (29)	5/9 (56)	5/9 (56)
**RD (A-B) (90% CI)**	10% (-30 to +47%)	-21% (-56 to +18%)	11% (-27 to +45%)	-21% (-60 to +24%)	-29% (-62 to +13%)	-10% (-58 to +42%)	-10% (-58 to +42%)

Table S7. Immune response rates by protocol version. Response rates are reported as the percentage of responses (n) out of participants (N) enrolled before and after the major protocol amendment. The difference in response rates between arms (A – B) is shown with 90% CI.

[a] Three of 33 were not evaluable for pRsp due to no sample at week 18 or week 25. Of the 30 evaluable, 5 were considered response failures due to dose limiting toxicities (DLTs) (n=4) or recurrence (n=1) despite no samples evaluable at week 18 or week 25. Two participants experienced late DLTs, but had evaluable samples for pRsp at week 18 or week 25.

[b] Of the 30 participants who were evaluable for pRsp, one was excluded from evaluation for mRsp due to having a missing week 25 sample but positive response at week 26, and thus was unevaluable for a 2-fold increase.
