# Supplementary material for: Randomized Phase I/II Clinical Trial of a Melanoma Helper Peptide Vaccine with or without Systemic Agonistic Anti-CD27 Antibody (Varlilumab)
Source: Cancer Res Commun. 2026 Apr 30;6(4):994–1005. doi: 10.1158/2767-9764.CRC-25-0744 (PMC13130881; doi:10.1158/2767-9764.CRC-25-0744)
Supplement: Table S8 — Changes in absolute number of circulating Tregs over time [file crc-25-0744_table_s8_suppst8.pdf]

	Estimate	95% CI	p value
**Change from baseline**			
**Arm A**			
**Week 3**	-0.34	-0.48 to -0.19	**<.0001**
**Week 12**	-0.66	-0.81 to -0.50	**<.0001**
**Week 25**	-0.29	-0.49 to -0.09	**0.0048**
**Week 26**	-0.50	-0.69 to -0.32	**<.0001**
**Arm B**			
**Week 3**	-0.0047	-0.14 to 0.14	0.95
**Week 12**	0.015	-0.12 to 0.15	0.83
**Week 25**	0.054	-0.10 to 0.21	0.48
**Week 26**	0.045	-0.11 to 0.20	0.55
**Difference Arm B – A**			
**Week 3**	0.33	0.13 to 0.54	**0.0015**
**Week 12**	0.67	0.46 to 0.88	**<.0001**
**Week 25**	0.34	0.093 to 0.59	**0.0077**
**Week 26**	0.55	0.31 to 0.79	**<.0001**
**Change from Week 25 to 26**			
**Arm A**	-0.21	-0.39 to -0.03	**0.021**
**Arm B**	-0.0083	-0.092 to 0.075	0.84
**Difference Arm B – A**	0.20	0.01 to 0.40	**0.043**

Table S8. Changes in absolute number of circulating Tregs over time. Changes in circulating Tregs from baseline to week 26 by repeated measures modeling of log10 transformed data. Significant $p < 0.05$, bolded.
