# Supplementary material for: Randomized Phase I/II Clinical Trial of a Melanoma Helper Peptide Vaccine with or without Systemic Agonistic Anti-CD27 Antibody (Varlilumab)
Source: Cancer Res Commun. 2026 Apr 30;6(4):994–1005. doi: 10.1158/2767-9764.CRC-25-0744 (PMC13130881; doi:10.1158/2767-9764.CRC-25-0744)
Supplement: Table S9 — Changes in absolute number of circulating CD4+ T cells over time [file crc-25-0744_table_s9_suppst9.pdf]

| | Estimate | 95% CI | p value |
|---|---|---|---|
| **Change from baseline** | | | |
| **Arm A** | | | |
| **Week 3** | -0.23 | -0.36 to -0.11 | **0.0005** |
| **Week 12** | -0.54 | -0.68 to -0.41 | **<.0001** |
| **Week 25** | -0.38 | -0.55 to -0.21 | **<.0001** |
| **Week 26** | -0.51 | -0.67 to -0.35 | **<.0001** |
| **Arm B** | | | |
| **Week 3** | -0.0032 | -0.13 to 0.12 | 0.96 |
| **Week 12** | 0.019 | -0.10 to 0.14 | 0.76 |
| **Week 25** | -0.047 | -0.18 to 0.086 | 0.48 |
| **Week 26** | 0.021 | -0.11 to 0.15 | 0.76 |
| **Difference Arm B – A** | | | |
| **Week 3** | 0.23 | 0.053 to 0.41 | **0.011** |
| **Week 12** | 0.56 | 0.38 to 0.74 | **<.0001** |
| **Week 25** | 0.33 | 0.12 to 0.54 | **0.0028** |
| **Week 26** | 0.53 | 0.33 to 0.74 | **<.0001** |
| **Change from Week 25 to 26** | | | |
| **Arm A** | -0.13 | -0.27 to 0.0011 | 0.052 |
| **Arm B** | 0.068 | 0.0062 to 0.13 | **0.032** |
| **Difference Arm B – A** | 0.20 | 0.054 to 0.35 | **0.0083** |

**Table S9. Changes in absolute number of circulating $CD4^+$ T cells over time.** Changes in circulating $CD4^+$ T cells from baseline to week 26 by repeated measures modeling of log10 transformed data. Significant $p < 0.05$, bolded.
