# Supplementary material for: Randomized Phase I/II Clinical Trial of a Melanoma Helper Peptide Vaccine with or without Systemic Agonistic Anti-CD27 Antibody (Varlilumab)
Source: Cancer Res Commun. 2026 Apr 30;6(4):994–1005. doi: 10.1158/2767-9764.CRC-25-0744 (PMC13130881; doi:10.1158/2767-9764.CRC-25-0744)
Supplement: Table S10 — Changes in circulating Tregs as a percentage of CD4+ T cells over time [file crc-25-0744_table_s10_suppst10.pdf]

	Estimate	95% CI	p value
**Change from baseline**			
**Arm A**			
**Week 3**	-0.11	-0.22 to 0.0091	0.070
**Week 12**	-0.11	-0.24 to 0.010	0.072
**Week 25**	0.081	-0.068 to 0.23	0.28
**Week 26**	-0.013	-0.15 to 0.12	0.85
**Arm B**			
**Week 3**	-0.026	-0.13 to 0.078	0.62
**Week 12**	-0.012	-0.12 to 0.094	0.83
**Week 25**	0.093	-0.027 to 0.21	0.13
**Week 26**	0.025	-0.095 to 0.15	0.68
**Difference Arm B – A**			
**Week 3**	0.081	-0.075 to 0.24	0.30
**Week 12**	0.10	-0.061 to 0.27	0.22
**Week 25**	0.012	-0.18 to 0.20	0.90
**Week 26**	0.038	-0.14 to 0.22	0.68
**Change from Week 25 to 26**			
**Arm A**	-0.094	-0.22 to 0.030	0.14
**Arm B**	-0.068	-0.12 to -0.018	**0.0087**
**Difference Arm B – A**	0.026	-0.11 to 0.16	0.70

Table S10. Changes in circulating Tregs as a percentage of $CD4^+$ T cells over time. Changes in circulating Tregs as a percentage of $CD4^+$ T cells from baseline to week 26 by repeated measures modeling of log10 transformed data. Significant $p < 0.05$, bolded.
