# Supplementary material for: Randomized Phase I/II Clinical Trial of a Melanoma Helper Peptide Vaccine with or without Systemic Agonistic Anti-CD27 Antibody (Varlilumab)
Source: Cancer Res Commun. 2026 Apr 30;6(4):994–1005. doi: 10.1158/2767-9764.CRC-25-0744 (PMC13130881; doi:10.1158/2767-9764.CRC-25-0744)
Supplement: Supplementary File 1 — Protocol [file crc-25-0744_supplementary_file_1_-_protocol_suppsp.pdf]

Cover Page for Protocol

The Mel65 Clinical Trial (NCT03617328) Protocol is attached, and includes a summary of all amendments since the original drafts. Several amendments were made prior to enrolling the first patient, as noted in section 9.6 "Summary of Changes". The enrollment began in September 2019; so, the protocol details summarized in this manuscript reflect the protocol at the time enrollment began.

UNIVERSITY OF VIRGINIA
HUMAN IMMUNE THERAPY CENTER

UVA-MEL-65
EVALUATION OF SAFETY AND DURABLE IMMUNOGENICITY OF MELANOMA VACCINATION, WITH OR WITHOUT SYSTEMIC CDX-1127, IN PATIENTS WITH STAGE II-IV MELANOMA

ATTACH TO THE FRONT OF EVERY COPY OF THIS PROTOCOL

Confidentiality Statement
The information contained in this document is the property of the University of Virginia Human Immune Therapy Center. Distributing, copying, or disclosing information contained in this document requires prior authorization from the Human Immune Therapy Center, except that this document may be disclosed to the appropriate institutional review boards and representatives of the U.S. Food and Drug Administration.

SIGNATURE PAGE

Sponsor

**Name**	**Signature**	**Date**

Principal Investigator

**Name**	**Signature**	**Date**

INVESTIGATOR'S AGREEMENT

I confirm that I have read this protocol and I agree to conduct the study as outlined herein. I agree to conduct the study in accordance with the ethical principles that have their origin in the Declaration of Helsinki and that are consistent with Good Clinical Practices, as outlined in ICH E6, and the applicable laws and regulations.

UNIVERSITY OF VIRGINIA
HUMAN IMMUNE THERAPY CENTER
UVA-MEL-65

EVALUATION OF SAFETY AND DURABLE IMMUNOGENICITY OF MELANOMA VACCINATION, WITH OR WITHOUT SYSTEMIC CDX-1127, IN PATIENTS WITH STAGE II-IV MELANOMA

Key Roles and Study Governance

**Sponsor**	Craig L. Slingluff, Jr., MD<br>University of Virginia Health System<br>Cancer Center Office of Clinical Research &<br>Human Immune Therapy Center<br>1340 Jefferson Park Ave.<br>Pinn Hall, Room 1352<br>Charlottesville, VA 22908
**Principal Investigator**	Craig L. Slingluff, Jr., MD
**Participating Institutions**	University of Virginia<br>Craig L. Slingluff, Jr., MD (Principal Investigator)<br>cls8h@virginia.edu<br>434-924-9311
**Biostatistician**	Gina R. Petroni, PhD<br>grp4c@virginia.edu
**Authors**	Craig L. Slingluff, Jr., MD Timothy Bullock, PhD<br>Kimberly Bullock, PhD Gina R. Petroni, PhD
**Data Coordinating Center**	University of Virginia School of Medicine, Clinical Trials Office<br><br>Gina Duda<br>GMS4X@hscmail.mcc.virginia.edu<br>(434) 924-9553

PROTOCOL SYNOPSIS 6

1.0 BACKGROUND AND SCIENTIFIC RATIONALE 12

1.1 SUMMARY 12
1.2 STUDY RATIONALE 12
1.3 STUDY DRUG: 6MHP 16
1.4 MONTANIDE ISA-51 24
1.5 POLYICLC 24
1.6 CDX-1127 25
1.7 HUMAN EXPERIENCE WITH THE COMBINATIONS OF 6MHP + POLYICLC + IFA WITH OR WITHOUT CDX-1127 IN THE FIRST 15 PARTICIPANTS ON THE PRESENT TRIAL 27
1.8 SUMMARY 28

2.0 OBJECTIVES 28

2.1 PRIMARY OBJECTIVES 28
2.2 SECONDARY OBJECTIVES 28
2.3 EXPLORATORY OBJECTIVES 29

3.0 PARTICIPANT SELECTION CRITERIA 29

3.1 INCLUSION CRITERIA 29
3.2 EXCLUSION CRITERIA 30
3.3 REGISTRATION AND RANDOMIZATION 32

4.0 STUDY DRUGS 33

4.1 6MHP VACCINE 33
4.2 POLYICLC (HILTONOL) 34
4.3 CDX-1127 (VARLILUMAB) 35
4.4 STUDY DRUG ACCOUNTABILITY 36

5.0 TREATMENT PLAN 36

5.1 MANAGEMENT OF PARTICIPANTS 36
5.2 ADMINISTRATION OF 6MHP, MONTANIDE ISA-51, AND POLYICLC 36
5.3 ADMINISTRATION OF VARLILUMAB (CDX-1127) 37
5.4 DOSE MODIFICATIONS 38
5.5 DOSE DELAYS 39
5.6 DISCONTINUATION OF THERAPY 41
5.7 REPLACEMENT OF STUDY PARTICIPANTS 42
5.8 CONCOMITANT MEDICATIONS 42
5.9 PERMITTED MEDICATIONS OR TREATMENTS 42
5.10 SUPPORTIVE CARE GUIDELINES FOR CDX-1127 43
5.11 MANAGEMENT OF TOXICITY 45
5.12 TREATMENT COMPLIANCE 45
5.13 BIOPSIES 45

6.0 CLINICAL AND LABORATORY EVALUATIONS 46

6.1 PHYSICAL EXAMS AND EVALUATIONS 46
6.2 PATHOLOGY REVIEW 46
6.3 PERFORMANCE STATUS 46
6.4 CLINICAL LABS 46
6.5 TOXICITY ASSESSMENTS 47
6.6 RESEARCH BLOOD SAMPLES 47

6.7 VACCINE SITE BIOPSIES 48
6.8 TUMOR BIOPSIES (OPTIONAL, AT THE TIME OF PROGRESSION) 48
6.9 ASSESSMENTS 50
6.10 STUDY CALENDAR 51

7.0 STATISTICAL CONSIDERATIONS 51

7.1 EVALUATION OF SAMPLE POPULATIONS AND ENDPOINTS 51
7.2 SAMPLE SIZE AND ACCRUAL 53
7.3 RANDOMIZATION 54
7.4 SAFETY MONITORING 54
7.5 ANALYSES 54

8.0 ADVERSE EVENT DATA COLLECTION AND MONITORING 55

8.1 DEFINITIONS 55
8.2 ATTRIBUTION ASSESSMENT 57
8.3 DATA COLLECTION 58
8.4 RISKS AND SAFETY 58
8.5 ADVERSE EVENT CLASSIFICATIONS 64
8.6 REPORTING ADVERSE EVENTS 65
8.7 ADVERSE EVENT REVIEW AND MONITORING 69
8.8 UNANTICIPATED PROBLEMS 70
8.9 DOSE-LIMITING TOXICITIES 70
8.10 DATA BREACH 71
8.11 PROTOCOL DEVIATION 72
8.12 DATA COLLECTION 73
8.13 MONITORING PLAN 73
8.14 STUDY CONDUCT AND ETHICAL CONSIDERATIONS 75
8.15 STUDY DISCONTINUATION AND CLOSURE 76

9.0 APPENDICES 77

9.1 STUDY CALENDARS 78
9.2 AJCC STAGING SYSTEM (AJCC VERSION 8) 83
9.3 ECOG PERFORMANCE STATUS 87
9.4 NEW YORK HEART ASSOCIATION DISEASE CLASSIFICATION 88
9.5 RECIST 1.1 CRITERIA 89
9.6 SUMMARY OF CHANGES 90

Protocol Synopsis

Title
Evaluation of Safety and Durable Immunogenicity of Melanoma Vaccination, With or Without Systemic CDX-1127, in Patients with Stage II-IV Melanoma (Mel65)

Investigational Drugs
Vaccines:
- 6 melanoma helper peptides (6MHP) (Table 1)

Local adjuvants:
- Montanide ISA-51 (an incomplete Freund's adjuvant)
- polyICLC (Hiltonol)

Systemic agent:
- CDX-1127 agonistic antibody to CD27

Table 1: Peptides used in the 6 Melanoma Helper Peptide (6MHP) vaccine

Amino Acid Sequence	*Epitope (source protein, residues)*	*Reference*
AQNILLSNAPLGPQFP	Tyrosinase $_{56\text{-}70}$ (alanine added#)	1
FLLHHAFVDSIFEQWLQRHRP	Tyrosinase $_{386\text{-}406}$	2
RNGYRALMDKSLHVGTQCALTRR	Melan-A/MART-1$_{51\text{-}73}$	3
TSYVKVLHHMVKISG	MAGE-3 $_{281\text{-}295}$	4
LLKYRAREPVTKAE	MAGE-1,2,3,6 $_{121\text{-}134}$	5
WNRQLYPEWTEAQRLD	gp100 $_{44\text{-}59}$	6,7
#An alanine residue was added to the N-terminus to prevent cyclization		

FDA approved drugs for the intended indication
N/A

Indication
Stage IIB-IV melanoma, plus stage IIA with high risk features (Class 2) on Melanoma DecisionDx or DecisionDX-UM assay. Most patients are expected to be clinically free of disease after surgery, but patients with small radiologic or clinical findings of an indeterminate nature may still be eligible.

Objectives and endpoints

Primary
1) Safety: To test the safety of CDX-1127 administered concurrently with a melanoma vaccine (6MHP). The melanoma vaccine will include Montanide ISA-51 and will be co-administered with polyICLC.

   Safety will be evaluated by adverse event assessments, including CTCAE v5.0 and sub-classified by irAE categories. [Endpoint: dose-limiting toxicities (DLTs) based on CTCAE v5.0 treatment related adverse events]

2) Immunogenicity: To determine whether addition of CD27 antibody to a melanoma vaccine improves the persistence of $CD4^{+}$ Th1 responses to vaccine antigens. [Endpoint: $CD4^{+}$ responses to 6MHP (by ELIspot), measured as the percent of patients with persistent responses (pRsp) at either day 127 or day 176, or both].

Secondary

1) To assess whether CD27 antibody decreases regulatory T cells ($FoxP3^+$ cells) in the vaccine site microenvironment at weeks 3 and 12. [Endpoint: in vaccine site biopsies $FoxP3^+$ $CD4^+$ T cells per mm^2 in by immunohistochemistry and gene expression analysis)].
   Note: with revision to reduce ulceration at vaccine sites, biopsies are not required at week 12 for participants whose week 12 visit would be due after IRB approval of Protocol v12-03-2020..
2) To determine whether addition of CD27 antibody to a melanoma vaccine,
   a. Decreases circulating regulatory T cells [Endpoint: circulating Tregs, % of $CD4^+$ T cells responding to vaccine antigens; $FoxP3^+$ $CD4^+$ T cells as measured by flow cytometry]
   b. Improves the frequency of $CD4^+$ Th1 responses to vaccine antigens [Endpoint: $CD4^+$ response to 6MHP measured by ELIspot; maximum increase after vaccination at any time point]
   c. Improves the frequency of durable $CD4^+$ Th1 responses to vaccine antigens (dRsp) [Endpoint: $CD4^+$ response to 6MHP measured by ELIspot; at two consecutive time points (day 8 to 85)]
   d. Increases the $CD4^+$ Th1 memory response (mRsp) to vaccine antigens at 6 months. [Endpoint: $CD4^+$ response to 6MHP by ELIspot a week after booster vaccine on day 176]

Exploratory

1. To assess the impact of CD27 antibody on induction of
   a. Antibodies to helper peptides in 6MHP (assessed in serum by ELISA for IgG). [Endpoint: IgG antibodies to 6MHP]
   b. $CD8^+$ T cell responses to melanoma antigens in the vaccine, or to other antigens by epitope spreading. [Endpoint: rate of $CD8^+$ T cell responses to peptides not in vaccine]
   c. $CD4^+$ T cell activation versus exhaustion. [Endpoint: proportion of $CD4^+$ and $CD8^+$ T cells that express PD-1, TIM-3, LAG-3, TIGIT with respect to HLA-DR as an activation marker by flow cytometry]
2. To obtain preliminary estimates of disease-free survival (DFS) and overall survival.

Design

This is an open-label, multi-center randomized phase I/II study

Regimen

All participants will receive a melanoma vaccine (6MHP) and will be randomized to receive (or not) systemic CDX-1127. The melanoma vaccine will be co-administered locally with polyICLC through Day 78. The polyICLC will be incorporated into the peptide emulsion with Montanide ISA-51 (IFA). On day 176, the melanoma vaccine will be administered without polyICLC. The schema is shown in Figure 1.

Figure 1: Protocol Schema
A

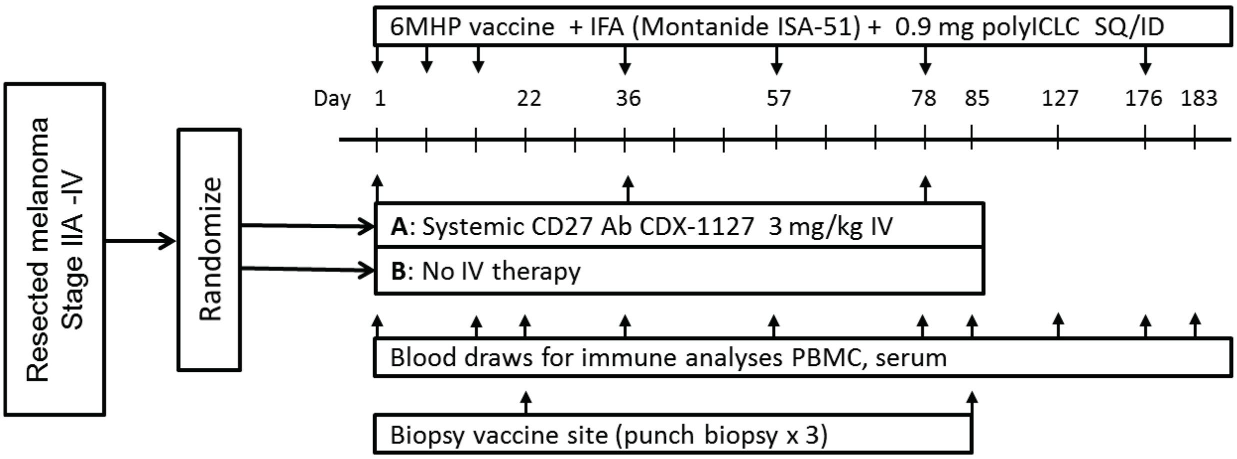

B

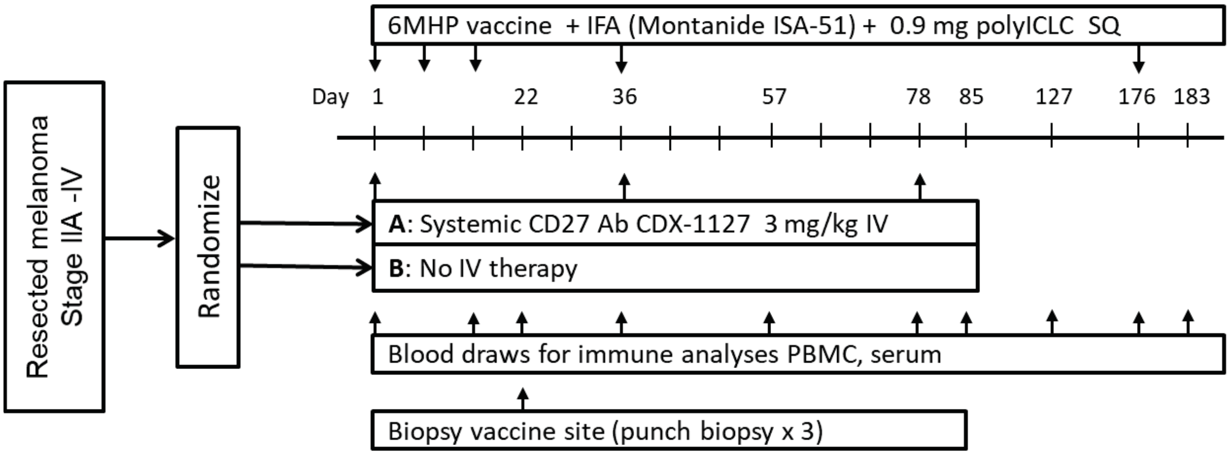

Figure 1. Clinical trial design. Patients will be enrolled in a phase I/II design to evaluate the safety and immunologic effects of adding agonistic CD27 antibody CDX-1127 to a cancer vaccine. Patients with stage IIB-IV melanoma at original diagnosis will be eligible, as well as some patients with high-risk stage IIA melanoma by the DecisionDx melanoma or DecisionDX-UM test. Patients will receive the multi-peptide vaccine 6MHP (200 mcg of each melanoma peptide). 6MHP vaccines will be administered with TLR3 agonist polyICLC (Hiltonol) in an emulsion with Montanide ISA-51. Immunologic effects will be assessed in peripheral blood. A booster vaccine (without polyICLC) administered day 176 will permit evaluation of recall memory responses a week later (d183). The study opened with the design shown in **(A),** but was revised after several patients had dose-limiting vaccine site ulcerations, with 3 changes to reduce the risk of vaccine site ulcerations (i) vaccines all administered subcutaneously rather than half-subcutaneous and half-intradermally, (ii) reduced from 7to 5 vaccines, and (iii) removed the vaccine site biopsy at week 12, as shown in **(B)**. Participants enrolled after approval of Protocol v12-03-2020 will follow schema B. Participants enrolled prior to the amendment, but who were between week 3 and 12 at the time the amendment was approved will follow the amended schema **(B)**.

Biopsies

Vaccine Site Biopsies: Each participant will undergo a biopsy of the vaccine site at day 22, one week after vaccine 3. For participants reaching week 12 prior to approval of the amendment to reduce vaccine site ulceration, a biopsy of the vaccine site is also performed on day 85, one week after vaccine 6. The biopsies will consist of three 4-mm punch biopsies of skin.

Population

The main criteria for inclusion include:

- Age 18 years or older.
- A diagnosis of melanoma at high risk of recurrence/metastasis after surgical or ablative therapy (stage IIB-IV). Patients with stage IIA melanoma who are found to be high-risk based on the DecisionDx Melanoma or DecisionDX-UM test may also be eligible.

Accrual Goal

Target accrual is estimated to be 30 eligible patients (15 per arm). Allowing for 10% ineligibility/dropout/lost-to-follow-up, up to 34 patients may be accrued.

Table 2: List of Abbreviations

Abbreviation	Full text
β–HCG	Beta Human chorionic gonadotropin (pregnancy test)
6MHP	6 melanoma-derived class II MHC-restricted helper peptides
12-MP	12 melanoma-derived class I MHC-restricted peptides
Ab	antibody
AE	adverse event
AJCC	American Joint Committee on Cancer
ALT	alanine aminotransferase
ANA	anti-nuclear antibody (serum test)
ANC	absolute neutrophil count
APC	antigen presenting cell
AST	aspartate aminotransferase
CC	Cancer Center
cc	cubic centimeter
CD27	Cluster of differentiation 27
CFR	Code of Federal Regulations
cm	centimeter
CRF	case report form
CT	computed tomography
CTA	cancer-testis antigens
CTCAE	Common Terminology Criteria for Adverse Events
CTL	cytotoxic T lymphocyte
DC	dendritic cells
dL	deciliter
DLT	dose limiting toxicity
dRsp	durable response
DSMC	Data and Safety Monitoring Committee
DTH	delayed type hypersensitivity
ECOG	Eastern Cooperative Oncology Group
ELISA	enzyme linked immunosorbent assay
FBS	fetal bovine serum
FDA	Food and Drug Administration
g	gram
GM-CSF	granulocyte-macrophage stimulating colony
GMP	good manufacturing practice
Hgb	hemoglobin
HGBA1C	hemoglobin a1c
HITC	Human Immune Therapy Center
HIV	human immunodeficiency virus
HLA	human leukocyte antigen
HPLC	high performance liquid chromatography
ID	intradermal
IFN	interferon
IL-2	interleukin-2
IL-7	interleukin-7
IML	Immune Monitoring Laboratory

In.	inch
IND	investigational new drug
IRB	Institutional Review Board
IV	intravenous
kg	kilogram
LC	Langerhans cells
LDH	lactate dehydrogenase
m	meter
mcg	microgram
mCy	metronomic cyclophosphamide
MDP	melanocyte differentiation proteins
mg	milligram
MHC	major histocompatability complex
ml	milliliter
mm	millimeter
MRI	magnetic resonance imaging
mRsp	memory response
NBT/BCIP	Nitro blue tetrazolium chloride/5-Bromo-4-Chloro-3-Indolyl Phosphate
NCI	National Cancer Institute
NSAID	non-steroidal anti-inflammatory drug
PBL	peripheral blood lymphocytes
PBS	phosphate buffered saline
PD-1	Programmed-death 1
PET	positron emission tomography
PI	principal investigator
PRC	protocol review committee
pRsp	persistent response
RF	rheumatoid factor (serum test)
SD	standard deviation
SIN	sentinel immunized node
SQ	subcutaneous
T_h	$CD4^+$ helper T cells
TIL	tumor infiltrating lymphocytes
TNF	tumor necrosis factor
ULN	upper limits of normal
USP	United States Pharmacopeia
UVA	University of Virginia
UVA CC	University of Virginia Coordinating Center
WBC	white blood cell

1.0 BACKGROUND AND SCIENTIFIC RATIONALE

1.1 Summary

Therapeutic blockade of immune checkpoint molecules has induced clinical benefit for patients with several cancers[12-14]. In counterbalance to checkpoint molecules are a set of costimulatory molecules, including CD27. CD27 contributes to activation and survival of T cells[15-17]; fully stimulated T cells may be resistant to deletion by checkpoint inhibitors [18,19]. Notably, CD27-stimulation lowers expression of inhibitory molecules on $CD8^+$ T cells, and reduces the frequency of regulatory T cells (Treg) in the tumor microenvironment (TME) [18]. Our experience with a fully human agonistic CD27 antibody (CDX-1127/Varlilumab, Celldex) indicates it is safe and bioactive in cancer patients when delivered systemically: it increases frequencies of activated ($HLA\text{-}DR^+$) CD8+ T cells and $CD56^{lo}$ NK cells, while the proportion of Tregs is decreased (NCT01460134 [20]). In a murine model, a CD27 antibody enhanced the immunologic benefit and tumor control induced with a tumor vaccine [21]. Thus, there is rationale for studying immunologic effects of agonistic antibodies to CD27, in combination immunotherapy with cancer vaccines. The present clinical trial will test the safety and immunologic outcomes of administration of an agonistic antibody to CD27 in conjunction with an antigen vaccine: an enhanced helper peptide vaccine, 6MHP. The present proposal will test the hypotheses that agonism of CD27 systemically, in conjunction with a TLR agonist, is safe and augments vaccine efficacy by optimizing T cell induction, improving T cell survival, and decreasing regulatory T cells.

Amendment to reduce vaccine site ulceration (12-03-2020): Three vaccine site ulcerations meeting criteria for DLTs were observed by 11/23/20, one on arm A and two on Arm B. There was no evidence that the CD27 Ab contributed to this risk. The study was held once earlier for DLTs on arm B which included a grade 3 pneumonitis and was reinitiated after review with the DSMC and modification to the study exclusion criteria. The second DLT, vaccine site ulceration on Arm B, required an additional study hold resulting in a study modification to reduce vaccine site ulceration by administering vaccines subcutaneously only, by limiting to 5 vaccines, and by removing the vaccine site biopsy at week 12.

1.2 Study Rationale

Melanoma is increasing in incidence faster than any other cancer, with over 76,000 new invasive melanomas diagnosed this year and more than 10,000 deaths expected[22].

Systemic management of melanoma in the adjuvant setting

The only FDA-approved therapies for melanoma in the adjuvant setting are high-dose interferon-alpha (HD-IFN), pegylated interferon-alpha (PEG-IFN) and high-dose ipilimumab (IPI). HD-IFN was approved for patients with resected stage IIB, IIC, and III melanoma; however, it is toxic enough that a large subset of patients fail to complete the treatment regimen and the clinical benefit is modest, at best, with about 10% of patients experiencing prolonged disease-free survival (DFS)[23]. However, overall survival (OS) benefit is less clear. The ECOG 1684 trial did show overall survival benefit in about 10% of patients[24], but the larger follow-up study ECOG 1690 showed no survival benefit ($p = 0.99$)[25]. Summary data from those and other studies support DFS benefit (HR 0.82) and a smaller OS benefit (HR 0.89) at the price of significant toxicity and uncertainty about the optimal dose and regimen [26]. A short course of HD- IFN was not effective [27]. An alternate interferon regimen also is approved for adjuvant therapy of stage IIB-III melanoma. This therapy uses pegylated interferon-alpha (PEG-IFN), which has delayed relapse at a level similar to that of HD-IFN, but definitive proof of a survival advantage remains to be

demonstrated, and its use is also limited by toxicity [28,29]. Since the benefit of current approved IFN therapy regimens is small and the toxicity high, many oncologists recommend that patients explore clinical trials, usually of melanoma vaccines.

The CTLA-4 blocking antibody, ipilimumab, has recently been approved for use in the adjuvant setting, and specifically for patients with lymph node metastases measuring at least 1 mm in diameter. This approval was based on the proven therapeutic value for advanced melanoma [30,31] and the outcome of a randomized phase III clinical trial of high-dose ipilimumab (10 mg/kg x up to 15 doses) vs observation for patients rendered clinically free of disease after resection of melanoma metastases in regional nodes, at least 1 mm in diameter[32,33]. Patients who received ipilimumab in the randomized phase III clinical trial (EORTC 18071) experienced improved relapse-free survival and overall survival, with HR of 0.76 and 0.72, respectively [32,34]. This clinical benefit, however, was associated with high toxicity rates, where grade 3-4 adverse events were reported in 54% of ipilimumab treated patients, vs 26% of the placebo patients. In particular, grade 3-4 immune-related adverse event rates were 42% and 3%, respectively. Also 1.1% of patients receiving this dose of ipilimumab died from treatment-related adverse events [32]. Thus, ipilimumab is a meaningful option for a subset of patients with stage III melanoma. However, it is not approved for high risk stage IIB-IIC melanoma, for stage III melanoma with intransit metastases, for stage III melanoma with metastases less than 1 mm in diameter, or for resected stage IV melanoma. Also, despite the proven benefit, the risk of treatment-related death and the risk of severe toxicity limits its use even in patients who are eligible for it. An ECOG-ACRIN trial E1609 has completed enrollment of stage IIIB-IV patients in a 3-arm randomized phase III trial of HD-IFN vs 3 mg/kg ipilimumab vs 10 mg/kg ipilimumab. That trial has the potential to identify benefit of the lower dose of ipilimumab.

Another immune checkpoint inhibitor, the PD-1 blocking antibody nivolumab, has also recently been approved for use in the adjuvant setting for patients with melanoma. This approval was based on the results of the randomized, double-blind, phase 3 BMS CA209-238 study comparing recurrence-free survival outcomes of patients who received 3 mg/kg nivolumab every 2 weeks versus 10 mg/kg ipilimumab every 3 weeks for 4 doses and then every 12 weeks for up to 1 year. Patients who received nivolumab had a 12 month rate of recurrence-free survival of 70.5% compared to a rate of 60.8% for subjects treated with ipilimumab [128]. In addition, treatment-related grade 3 and 4 toxicities were reported in 14.4% of patients in the nivolumab group and in 45.9% of those in the ipilimumab group. PD-1 blocking antibodies have shown a favorable safety profile and improved efficacy and durability in patients with melanoma compared with ipilimumab, but overall checkpoint inhibitors carry the risk of serious immune-related and in some instances potential life-threatening toxicities. Long-term risks from checkpoint inhibitors are still under investigation.

Thus, available options for adjuvant therapy for melanoma include regimens that are approved for stage II-III melanoma, but the use of these therapies is limited by their toxicity profiles, and for HD-IFN and PEG-IFN, are also limited by their modest impact on overall survival. Because of these limitations, many patients who would be eligible for them do not take them and are interested in adjuvant therapy trials with prospect of lower toxicity. Thus, there is a strong and continuing need for adjuvant therapy trials for patients with resected stage II-IV melanoma. Cancer vaccines offer the prospect of low toxicity[35], and are good options to consider for these patients.

<u>Status of cancer vaccines targeting CD8$^+$ T cells</u>. Peptide vaccines for melanoma offer the promise of inducing CD8$^+$ T cells reactive to well-characterized tumor antigens and also enabling assessment of effectiveness of the vaccinations by monitoring antigen-

specific T cell responses[36]. However, clinical experience with peptide vaccines has been mixed[37,38]. On one hand, we and others have found that selected peptides can induce circulating T cell responses in a majority of patients, and that vaccination with a mixture of peptides can be immunogenic in up to 100% of patients [36,39-41]. In some trials, circulating $CD8^+$ T cell immune responses have correlated with clinical outcome [36,42], but this has not been the rule. Also, the magnitude of T cell responses sometimes is substantial, with 1-5% of circulating $CD8^+$ T cells reactive to a single antigen [41,43]; however, responses in most patients represent T cell percentages that are 1-2 orders of magnitude lower, which may or may not be adequate for clinical benefit. The T cell responses to vaccines may be very durable, for months or years, but are at least as likely to be transient, sometimes declining even while still receiving vaccines [44]. Clinically, a peptide vaccine stimulating $CD8^+$ T cells has enhanced survival of patients with advanced melanoma when combined with high dose IL-2 [45]; however, overall clinical response rates with vaccines targeting CD8 T cells have averaged about 3% [38]. Thus, clinical activity of vaccines targeting $CD8^+$ T cells has been disappointing. Opportunities for improving these vaccines include combination with other agents that may enhance T cell responses and modulate tumor-associated immune dysfunction.

Promise of cancer vaccines targeting $CD4^+$ T cells (helper peptide vaccines). In contrast to vaccines targeting CD8+ T cells, "helper peptide" vaccines are designed to induce $CD4^+$ "helper" T cell responses to melanoma antigens. We have performed 4 clinical trials with a helper peptide vaccine (6MHP), comprising 6 melanoma-derived helper peptides. The 6MHP vaccine, administered in an incomplete Freund's adjuvant (IFA, Montanide ISA-51) has been immunogenic in up to 80% of patients [46], and multiple findings suggest that this approach may overcome many of the limitations of vaccines designed to induce $CD8^+$ T cells. Among 91 patients with measurable disease treated in two clinical trials, 7 experienced objective clinical responses (8% ORR)[46,47], and there has been durable clinical benefit, with stable disease and clinical responses lasting 1 – 7 years[46]. In addition, patients with resected stage IV melanoma have experienced an encouraging 74% five-year survival, exceeding that of untreated institutional controls, in an era prior to checkpoint blockade therapy[48]. Thus, there is evidence of clinical activity and clinical benefit with this vaccine alone. Also, unlike the experience with $CD8^+$ T cell responses, the $CD4^+$ T cell responses to the 6MHP vaccines have correlated significantly with overall survival in two separate clinical trials[47,49]. Interestingly, the antibody response to those peptides also correlates with overall survival, and the strongest correlation is for those patients with both antibody (IgG) and $CD4^+$ T cell responses[49].

The mechanisms by which helper peptide vaccines mediate benefit are being elucidated. We have found that these vaccines predominantly induce Th1-type $CD4^+$ T cell responses and that they also induce epitope spreading to $CD8^+$ T cells[50,51]. The induction of IgG antibody to the peptides suggests an integrated immune response and may support T cell responses by opsonizing the peptides for better antigen presentation[49].

One limitation of vaccines specifically targeting $CD8^+$ T cells is the fact that the short peptides used for those vaccines bind very specifically to a single class I MHC molecule, limiting their relevance to patients with those HLA alleles. On the other hand, helper peptides commonly bind much more promiscuously to a wide range of class II MHC molecules. We have found that the 6MHP vaccine induces $CD4^+$ T cell responses restricted by a wide range of HLA-DR molecules, such that the 6MHP vaccine may be employed in patients without any selection for their HLA types[51].

Toxicity with the 6MHP vaccines is low, and it is notable that higher-grade injection site reactions are much less common with 6MHP vaccine than with a mixture of 12 class I MHC-restricted peptides[52]. Thus, the 6MHP vaccine platform offers promise as an experimental therapy that may be employed safely in the adjuvant setting, with evidence of clinical activity, and an integrated immune response.

Limitations of cancer vaccines and need for improved adjuvants. Despite the promise of cancer vaccines, and of the 6MHP vaccines in particular, cancer vaccines are not optimized. Both local adjuvants and systemic immune modulators may be improved. Some data have challenged the effectiveness of the current formulation of incomplete Freund's adjuvant (IFA) with a peptide vaccine, especially for induction of Th1/Tc1 T cell responses [53], while data from our own experience supports its adjuvanticity [54]. However, data from our own experience, and from our collaborators also show that vaccines using short peptides in IFA induce chronic inflammation at the site of vaccination that attracts and retains antigen-specific T cells at the vaccine site and may cause T cell dysfunction [55,56]. Thus, there is a need to optimize adjuvants.

We are completing analysis of data from a trial testing local and systemic adjuvants for the 6MHP vaccines (Mel63, NCT02425306), and have found that T cell responses are enhanced by addition of the TLR agonist polyICLC to IFA locally. That trial and the preliminary data are summarized in Section 1.3.3 and below. These findings provide a promising strategy to build on. The present clinical trial is designed to test whether an agonistic CD27 antibody may enhance immune responses to this improved 6MHP helper peptide vaccine regimen.

Immune costimulatory molecules. Therapeutic blockade of immune checkpoint molecules induces clinical benefit in human cancers [30,65-68]. Similarly, antitumor immunity may also be augmented by agonism of costimulatory molecules, in particular, CD27 [69], which directly activates T cells. Fully stimulated T cells may be resistant to deletion by checkpoint inhibitors [18,19]. Thus, there is rationale for testing this agonistic antibody in combination immune therapy.

Role of CD27 and TLR stimulation as alternative signal 3 for T cell survival and differentiation: Optimal T cell activation and expansion depend on three signals. Signal 1: T cell receptor engaging peptide/MHC; Signal 2: CD28 on T cells engaging CD80/CD86 on a professional antigen-presenting cell (APC; dendritic cell, DC). Signal 3: cytokines (IL-12 or type-1 interferons). Agonism of CD27 (on T cells) by CD70 (on DC) also supports T cell activation, and can mediate signal 3 as an alternative to cytokines, thus supporting T cell survival and T cell memory [15,70,71] and polarization toward Th1[72].

The roles of CD70 and CD27 in T cell stimulation. A critical outcome of CD40 stimulation of DC is expression of the TNF-superfamily member, CD70, which in turn stimulates its receptor, CD27 [15]. CD27 is critical for expansion and survival of $CD8^+$ T cells responding to CD40-stimulated DC[15]. Direct CD27 stimulation of $CD8^+$ T cells overcomes the requirement for $CD4^+$ T cells in the response to vaccinia immunization[16]. CD27 stimulation supports effector $CD8^+$ T cell survival by sustaining expression of anti-apoptotic molecules such as Bcl_{XL} and promoting IL-2 expression [17]. CD27 stimulation also slows growth of transplantable melanomas in an NK and $CD8^+$ T cell-dependent manner; CD27-stimulation of $CD8^+$ T cells increases their persistence in tumor, and these cells are able to retain their function. Notably, CD27-stimulation lowers expression of inhibitory molecules on $CD8^+$ T cells, and reduces the frequency of regulatory T cells (Treg) in the TME [18,73].

The function and survival of antitumor T cells are limited by tumor-associated immune dysfunction and possibly by activation-induced cell death. Activation of CD27 is crucial for T cell survival [74,75], through anti-apoptotic signaling Bcl-x(L) upregulation and Pim-1 induction [17]. CD27 activation also may recruit TRAF2, which can protect cells from TNFα-mediated apoptosis [76-78]. Also, CD27 is critical for T cell persistence and memory [15,70,71,75,79].

The impact of CD27 and CD70 on regulatory T cells (Treg) is debated. On one hand, CD70 expressed by human DCs can promote effector T cell activity and diminish Treg function in vitro[80]. On the other, murine studies suggest that CD70 is necessary for Treg development in the thymus [81] and presence and function in tumors[82], suggesting that Treg may require tonic CD70 signaling. Studies from the Bullock laboratory indicate that intra-tumoral Treg numbers are reduced by stimulating CD27, concomitant with an increase in $CD8^+$ T cell frequencies, resulting in a substantial favorable shift in the CD8:Treg ratio [18]. Particularly relevant are data from the Phase I trial of CDX-1127, where patients treated with the agonistic CD27 Ab had reduced circulating regulatory T cells by day 29 [83]. Reducing Tregs has been a therapeutic goal in cancer therapy; so, this effect would be significant if it is observed in this study, both in circulation and in the vaccine site microenvironment.

Human experience with a fully human agonistic CD27 antibody (CDX-1127/Varlilumab, Celldex)

The CD27 Ab varlilumab has been evaluated as monotherapy in a phase I clinical trial (NCT01460134), and a dose of 3 mg/kg has been identified as safe for future studies [83]. The data from the phase I trial indicate it is safe and bioactive in cancer patients when delivered systemically: it increases frequencies of activated ($HLA\text{-}DR^+$) T cells while the proportion of Tregs was decreased (NCT01460134)[83]. This increase in frequencies of activated T cells was most prominent for $CD4^+$ T cells [83], which supports use of this antibody in combination with a vaccine designed to expand $CD4^+$ tumor-reactive T cells. There was also evidence of clinical activity with this monotherapy. An ongoing trial is testing CD27 Ab CDX-1127 in combination with PD-1 blockade (nivolumab) in a range of solid tumors (NCT02335918).

Goals of the present study: The present study is designed primarily to obtain preliminary data on the effect of the agonistic CDX-1127 antibody on T cell responses to a promising cancer vaccine, 6MHP. The study will also formally test the induction of T cell memory, and whether it is enhanced by CDX-1127, and also whether CDX-1127 decreases Tregs in patients receiving these vaccines. Hypotheses underlying this study are that agonism of CD27, combined with a toll-like receptor (TLR) agonist and helper peptide vaccine, will decrease regulatory T cells, optimize antitumor T cell induction, and improve T cell survival.

Vaccine: 6 melanoma helper peptides (6MHP) (Table 1)

Local adjuvants:

- Montanide ISA-51 (an incomplete Freund's adjuvant)
- polyICLC

Systemic agent:

- CDX-1127 agonistic antibody to CD27

1.3 Study Drug: 6MHP

1.3.1 Rationale for Use of 6MHP

In the current protocol, we are including class II MHC-restricted peptides derived from melanoma proteins in an effort to generate melanoma-specific T_h responses. The melanoma specific class II MHC-restricted peptides (Table 1) are derived from melanocytic differentiation proteins (MDP) and cancer-testis antigens (CTA). The peptides were originally reported to bind to HLA-DR1, -DR4, -DR11, -DR13, and/or -DR15, and approximately 90% of the melanoma patient population will express at least one of

those class II alleles. Our prior work has demonstrated that these peptides, like other HLA-DR restricted peptides are also presented promiscuously on many other HLA-DR molecules [51]; thus, we do not restrict enrollment based on HLA expression.

The melanoma-associated class II MHC-restricted peptides in the 6MHP vaccine include 4 from MDPs (tyrosinase (2), gp100 (1), MelanA/MART-1 (1)), and 2 from CTAs (MAGE proteins). The first report of HLA-DR-restricted peptides recognized by T-cells on melanoma identified tyrosinase$_{56-70}$ and tyrosinase$_{448-462}$ [1]. Both peptides require high concentrations to induce T cell responses, but the former peptide has a higher binding affinity for HLA-DR4 than the latter; therefore, tyrosinase $_{56-70}$ (QNILLSNAPLGPQFP) was chosen for use in this study. Peptides with glutamine (Q) at the N-terminus can be unstable, as the glutamine can cyclize to form pyroglutamate [84]. To avoid that, we added an alanine residue to the N-terminus, to stabilize the glutamine residue. A DR15-restricted peptide, tyrosinase $_{386-406,}$ was reported also to be an antigen for T_h cells and was selected for use in this study [2]. Peptides presented by HLA-DR4 from MART-1/Melan-A have been identified [3], and we have chosen to include Melan-A/MART-1$_{51-73}$ in this trial. The last MDP represented in the vaccine is gp100, from which a peptide at residues 44-59 has been identified. T-cells sensitized against this peptide can recognize melanoma cells, and this epitope has been demonstrated to be naturally processed and presented in the context of HLA-DR4 [6,7,85].

The peptides from CTA to be included are from MAGE proteins. The peptide MAGE-A3 $_{281-295}$ can stimulate peptide reactive CTL in vitro and is strongly recognized by DR11-restricted MAGE-3 reactive CTL[4,5]. Another MAGE peptide is homologous with MAGE-1, 2, 3, and 6, and is restricted by DR13. It represents MAGE $_{121-134}$ [5].

The mixture of 6 peptides has been evaluated at 3 dose levels (200, 400, 800 mcg/dose) with no evident difference in immunogenicity or safety [46]; thus, we have used the lowest dose of 200 mcg per vaccine. The peptides for this trial have been synthesized under GMP conditions by a commercial provider. Prior experience has been with peptides made by Polypeptide Group, San Diego, California. They have been vialed under GLP conditions in our HITC lab clean room, which has limited access, an entry room for gowning and gloving, and positive pressure ventilation and laminar flow hoods. Lot release testing of the final vialed peptides will be completed in accord with CFR guidelines. Stability testing will be conducted regularly, in accord with the chemistry and manufacturing section of the IND application.

1.3.2 Pre-clinical Experience

There is no murine system adequately modeling the human immune response to these peptides. The most meaningful evaluation of this peptide vaccine mixture is in patients with melanoma. This trial is for participants with resected stage IIB/C, III, or IV NED melanoma. These individuals face a high risk (> 40%) of death from melanoma, and the anticipated risk of short-term or long-term toxicity of this vaccine preparation is minimal, while the vaccine may delay or decrease the risk of morbidity and mortality due to melanoma in these participants.

1.3.3 Previous Human Experience

Immunogenicity and Clinical Activity

This protocol builds on 4 prior NIH-funded clinical trials performed at the University of Virginia and collaborating centers:

a) Mel41 (NCT00089219): Phase I/II first-in-humans trial of 6MHP vaccine in stage III/IV melanoma (supported by R21 CA105777)[46];

b) Mel44 (NCT00118274): testing multi-peptide vaccines administered with cyclophosphamide to stimulate $CD8^+$ and $CD4^+$ T cells in patients with high-risk melanoma (supported by R01 CA118386)[40],

c) ECOG trial E1602 (NCT00071981) testing multi-peptide vaccines to stimulate $CD8^+$ and $CD4^+$ T cells in advanced melanoma (supported by R01 CA104362)[47],

d) Mel63 (NCT02425306), testing 6MHP vaccine plus each of 4 adjuvant preparations. This trial has just completed enrollment.

Together these trials demonstrate the safety, immunogenicity, and clinical activity of the 6MHP vaccine.

Table 3: Clinical response rates to 6MHP vaccines in patients with advanced melanoma in Mel41 and E1602 trials (RECIST)

Study	N	CR+ PR	CR+PR +SD	RR	DCR
Mel41	17	2	4	12%	24%
E1602 Arm D	42	3	15	7.1%	36%
E1602 Arm C	32	2	8	6.3%	25%
**All studies**	**91**	**7**	**27**	**7.7%**	**30%**
Mel41+Arm D	59	5	19	8.5%	32%

Vaccination with 6MHP was immunogenic in 81% of patients with stage III-IV melanoma, when administered with Montanide ISA-51 (an incomplete Freund's adjuvant, IFA) + GM-CSF as the vaccine adjuvant, and when measuring immune responses in vaccine-draining nodes (sentinel immunized nodes)[46]**.** When immune responses are measured in peripheral blood, they are detected in 40-60% of patients [40,46,47]. In patients with advanced melanoma, this vaccination strategy has been associated also with clinical regressions in 7-12% of patients and with stable disease for an additional 12-29% (mean disease control rate, DCR, 30%) as shown in Table 3. In the Mel41 trial, durations of SD and clinical responses have ranged from 1 to 7 years [46].

In a separate study we found that GM-CSF did not increase immunogenicity compared to IFA alone. Instead, we found that IFA alone was a better adjuvant than IFA+GM-CSF for induction of $CD4^+$ T cell responses to an intermediate length helper peptide[41]. Though GM-CSF continues to be used as a vaccine adjuvant in other settings, studies by us and others [86] provide randomized prospective data on its use in humans in combination with other adjuvants - in both cases, the addition of GM-CSF was associated with lower immunogenicity, worse clinical outcome, or both [41,86]. Thus, in a subsequent trial, we evaluated the 6MHP vaccine in Montanide ISA51, without GM-CSF, in patients with no clinical evidence of disease (resected

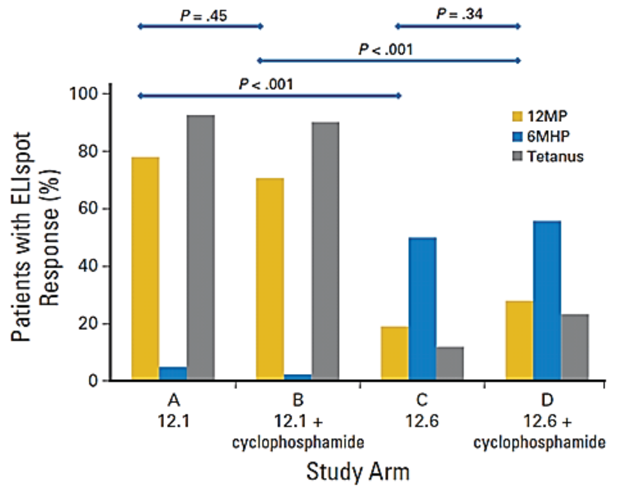

Figure 2: Mel44 trial. Immune response rates for 12MP, 6MHP, and tetanus peptide by ELISpot assay for each study arm, by evaluation of PBMC through day 50. *P* values for the response to 12MP are noted for four pairs of study arms. 12.1 [MELITAC 12.1], vaccines with 12MP plus tetanus peptide AQYIKANSKFIGITEL; 12.6 [MELITAC 12.6], vaccines with 12MP plus 6MHP.

stage IIB-IV) [40]. Immune responses to 6MHP in the blood were detected in 48% of patients.

We tested, in two trials (Mel44 and E1602), whether co-administration of the 6MHP vaccine with a peptide vaccine that stimulates $CD8^+$ T cells (12 MHC Class I restricted peptides, 12MP [36]) would induce greater $CD8^+$ T cell reactivity and increased clinical responses than vaccination with 12MP alone. In the Mel44 trial, patients were vaccinated with 12MP+6MHP (MELITAC 12.6) or 12MP+tetanus helper peptide (MELITAC 12.1), with IFA alone as the adjuvant, in patients with resected stage IIB-IV melanoma and no measurable disease. That trial was designed to enroll 160 eligible patients, plus allowances for ineligibility and over-enrollment. We enrolled 170 patients at 3 centers. Immune responses were evaluable for 161 (96%) of 167 eligible patients. T cell responses were measured by IFNγ ELIspot assay directly ex vivo, among $CD8^+$ T cells for 12MP, and among $CD4^+$ T cells for tetanus helper peptide (arms A and B), and 6MHP (arms C and D).

The combination of 6MHP with 12MP paradoxically reduced the circulating $CD8^+$ T-cell response rate (Figure 2)[40]. Responses to the tetanus helper peptide were detected in 91% of patients vaccinated with it, and the mixture of 6 melanoma helper peptides (6MHP) induced T_H cell responses in 52% of those vaccinated with it[40]. Patients on the Mel44 trial were randomized to pre-treatment with one dose of cyclophosphamide (CY, 300 mg/m^2), which had no effect on $CD8^+$ or $CD4^+$ T cell responses (Figure 2). Clinical outcome was not altered by adding 6MHP or CY to 12MP.

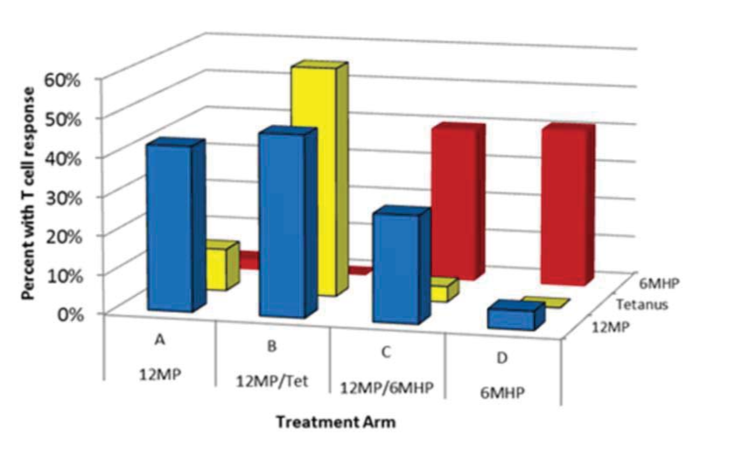

Figure 3. **E1602 trial. Immune responses to 12MP, 6MHP, and tetanus peptide.** A) The proportions of analyzable patients with CTL response to 12MP (blue bars), helper T cell response to tetanus peptide (yellow bars), and helper T cell response to 6MHP (maroon bars) are shown for each study arm.

In the E1602 trial, patients with advanced measurable melanoma were vaccinated in a 4-arm randomized phase II design: Patients on arms A, B, C, and D were vaccinated with 12MP, 12MP+tetanus helper peptide, 12MP+6MHP, or 6MHP alone, respectively, and with IFA+GM-CSF as vaccine adjuvant[47]. Similar to the finding in the Mel44 trial, the combination of 12MP+6MHP did not induce greater $CD8^+$ T cell responses or better clinical outcome. However, T cell responses were observed in the peripheral blood in about 40% of patients vaccinated with 6MHP (Figure 3), and objective clinical responses were observed in arms C and D: 2 partial responses (PR) in Arm C (6.3%) and 3 in Arm D (7.1%) (Table 3). There was additional evidence of clinical benefit, as the 1 year survival for Arms C and D exceeded the 95% confidence interval from a meta-analysis of outcomes from cooperative group trials (Figure 4). Importantly, there was also a significant prolongation of survival for patients who had a helper T cell response to the 6MHP vaccines (Arms C+D, $p = 0.005$, Figure 4). That difference persisted in a multivariate analysis ($p = 0.038$, HR 0.5 [47]). The

association between immune response to helper peptides was specific for the 6MHP, as no difference in survival was observed with immune response to the tetanus peptide for Arm B (not shown).

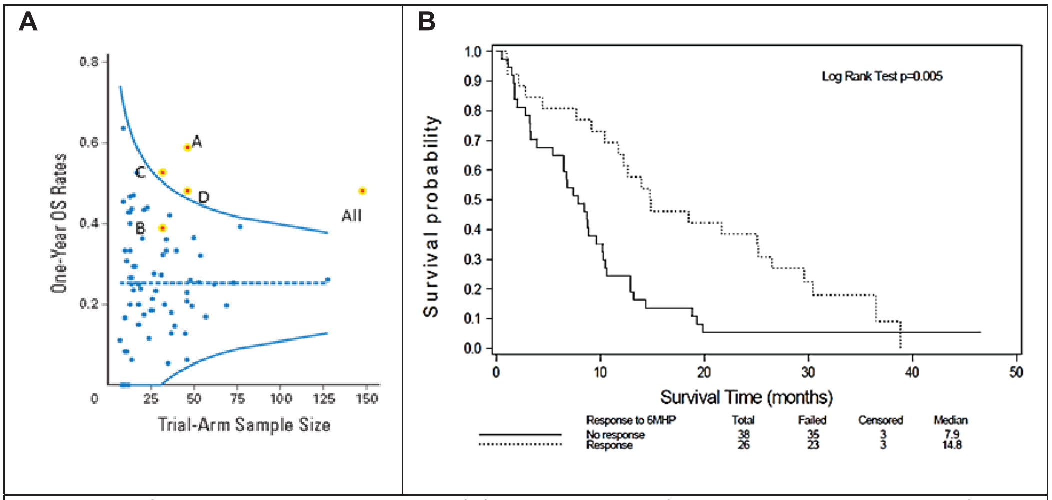

Figure 4. Overall survival on E1602. (A) 1 year survival for each treatment arm and for the full study population is shown on the plot of 1 year OS for all melanoma trials as previously published by Korn et al[87] and (B) Overall survival by helper T cell response to 6MHP in analyzable patients on arms C and D of E1602 trial (n = 64).

In the Mel63 trial, patients with resected stage IIA-IV melanoma were enrolled to one of 4 arms, where 4 different vaccine adjuvant combinations were evaluated:

a) Montanide ISA-51 (IFA) alone
b) Montanide ISA-51 + oral metronomic cyclophosphamide (mCy)
c) Montanide ISA-51 + polyICLC
d) Montanide ISA-51 + polyICLC + mCy

Final immune response data are shown in Table 4 below:

Table 4: Assessment of immune response by ELIspot assay, Mel63 trial

Arm	dRsp 6MHP in PBL #/n (%)	Any2Rsp 6MHP in PBL #/n (%)	Min, max # ≥ 5 in PBL		SIN #/n (%)	Min, max # ≥ 5 w/SIN		dRsp 90% CI	Any2Rsp 90% CI
A	0/3 (0)	0/3 (0)	0,1		0/3 (0)	0,1		(0, 63)	(0, 63)
B	2/7 (29)	2/7 (29)	0,3		2/6 (33)	0,4		(5, 66)	(5, 66)
C	3/6 (50)	4/6 (67)[a]	0,5		5/5 (100)	0,6		(15, 85)	(27, 94)
D	16/32 (50)	18/32 (56)[b]	0,7		14/30 (47)	0,8		(34, 66)	(40, 71)

a: #26 rsp at wks 5 & 12
b: #33 rsp at wks 2, 12, 18, 26, 32, #43 rsp at wks 3 & 12

A dRsp is defined as at least a 5-fold increase in immune response to the 6MHP peptide as measured by $CD4^+$ T cells over at least two consecutive time periods during vaccination (days 0 to 85). Any2dRsp is defined as at least a 5-fold increase in immune response to the 6MHP peptide as measured by $CD4^+$ T cells over the full time period (days 0 to 225).

Inclusion of polyICLC appears to enhance immune responses (C vs A, D vs B). Addition of systemic mCy may enhance immune responses compared to IFA alone (B vs A), but

these numbers are very small. However, mCy does not appear to enhance immune responses when added to IFA + polyICLC (D vs C). The immune response rate with IFA + polyICLC is excellent and appears similar to that with addition of mCy.

Toxicity review also shows that there were 3 participants with DLTs: one on Arm B and two on Arm D. These are both arms with mCy (Table 5).

Table 5: DLTs on Mel63 clinical trial

Arm	Arm Description	# pts with DLT	# pts total	%	DLT pts seq# (time)
A	6MHP + IFA	0	3	0.0	
B	6MHP + IFA + mCy	1	7	14.3	22 (day 8)
C	6MHP + IFA + polyICLC	0	6	0.0	
D	6MHP + IFA + polyICLC + mCy	2	32	6.3	7 (day 22), 36 (day 85)
	**Total**	3	48	6.3	

Though it is not clear that mCy was causative for those DLTs, this, plus the immune response data, support future vaccination with peptides in IFA + polyICLC.

<u>Summary of Preliminary Immunogenicity and Clinical Response Data with 6MHP Vaccines</u>

A consistent finding from these 4 trials is that 6MHP vaccines are immunogenic, with 40-50% immune response rate, when measured in blood, and 81% when measured also in vaccine-draining nodes. Also, in the 2 trials that enrolled patients with measurable disease, there were durable clinical responses [46], and there was clinical activity with an 8% RECIST-defined response rate, and 30% disease control rate (Table 3) similar to those of ipilimumab (RR 11%, DCR 29%)[31] and durable clinical responses [47]. Importantly, in E1602, there was a strong and specific association between immune response to the 6MHP and survival, support the clinical relevance of immune responses induced to the 6MHP vaccine [47].

<u>Toxicities Previously Reported for Participants Receiving the 6MHP Vaccine with Montanide ISA- 51 adjuvant, with or without GM-CSF</u>

There is no reason to expect direct toxicity of the melanoma peptides; they are not directly cytotoxic *in vitro* and are not expected to have functional implications. On the other hand, because some of these peptides are identical or similar to a portion of a normal protein, risks of autoimmunity in humans are reasonable to evaluate. The potential implications of autoimmunity against cells of melanocytic lineage are illustrated by reported cases of vitiligo occurring coincident with regressions of melanoma [88,89]. Most of these are limited, often occurring in skin surrounding the regressing melanoma, but occasionally occurring systemically. While pathogenesis of this phenomenon can only be hypothesized, it is reasonable to consider this a worst-case scenario.

The loss of skin and hair pigment can be striking in cases of vitiligo, but is not a cause of morbidity or mortality. Of greater potential concern is the theoretical risk of damage to the retinal pigment epithelium; however, visual loss has not been reported either as a complication of successful immunotherapy with melanoma-reactive CTL or spontaneous

vitiligo. However, some ocular toxicity has been reported that resolved with steroid treatment [90].

Depigmentation of the retinal pigment epithelium has been observed in a small number of patients vaccinated with dendritic cells pulsed with MDP-derived peptides; however, this change was asymptomatic and was not associated with loss of visual acuity (personal communication – Frank Haluska). A careful study of the retinal pigment epithelium using monobenzyl ether of hydroquinone to induce pigment cell destruction on a biochemical basis suggests the safety of pigment cell destruction and supports immunotherapy directed against MDP as a strategy for melanoma therapy (personal communication, JM Kirkwood).

Thus far, 10-15% of participants receiving peptide-based vaccines in the studies at UVA were diagnosed with definite vitiligo[52]. In all cases, the vitiligo was asymptomatic. No major visual/ocular toxicity was reported for participants on these prior trials.

This trial will focus on participants with high risk resected melanoma (stage IIB/C, III, or IV melanoma without measurable disease; and stage IIA melanoma found to be high risk based on gene expression profiling). These individuals face a high risk (> 35%) of premature death, and the anticipated risk of short-term or long-term toxicity of this vaccine preparation is minimal, while the vaccine may delay or decrease the risk of morbidity and mortality due to melanoma in these patients [46,47].

The 6MHP was administered to 209 humans in 3 clinical trials (UVA trials Mel41 [NCT00089219][46] and Mel44 [NCT00118274][40], and ECOG trial E1602 [NCT00071981][47]). Treatment-related adverse events were graded using the NCI Common Terminology Criteria for Adverse Events v 2.0 or 3.0. Overall, the vaccines were well-tolerated. The majority of adverse events were Grade 1 and Grade 2. The most common adverse events (>10%) included:

- injection site reaction
- fatigue
- headache
- rigors/chills
- nausea
- sweating
- myalgias
- arthralgias
- fever
- dizziness
- anorexia
- diarrhea
- cough
- allergic rhinitis
- nasal/paranasal reactions

Toxicity data recorded for 47 participants enrolled on the Mel63 study also support the safety of the 6MHP vaccine. Toxicities were graded using CTCAE v 4.03. The majority of adverse events recorded were Grade 1 and Grade 2. The most common adverse events (>10%) reported included:

- nausea
- chills
- fatigue
- fever
- flu-like symptoms
- injection site reactions
- lymphocyte count decreased
- mucositis oral
- anorexia
- arthralgia
- myalgia
- headache
- cough
- skin induration
- skin ulceration

There were 3 DLTs reported in Mel63.

Arm B: One DLT in 6 patients (17%). One patient had an SAE requiring hospitalization for sepsis, due to an infected knee joint, which was attributed to a skin infection after a fall. This was considered unrelated to the vaccine or local adjuvants.

Arm D: Two DLTs in 28 patients (7%). One patient experienced grade 2 dyspnea that limited exercise. The grade 2 dyspnea recovered spontaneously after discontinuation of the vaccines. This event did not meet the protocol's formal definition of a DLT; however, we reported the event as a DLT because the patient chose to avoid further toxicity and came off study early. One patient experienced a grade 3 vaccine site reaction with ulceration, which healed.

Note, seven additional patients experienced grade 3 injection site reactions, but they did not meet the definition of DLTs.

<u>Ocular Toxicities with 6MHP</u>

Ocular toxicity has been reported in participants receiving the 6MHP vaccine in combination with checkpoint inhibitors. A UVA Mel 64 study participant developed posterior uveitis, which was attributed to treatment with pembrolizumab and possibly related to the combination of pembrolizumab and the 6MHP vaccine. The condition resolved with steroid treatment (unpublished data). A participant on the UVA Mel 62 study developed VKH-like panuveitis syndrome, which was determined to be possibly related to the combination of 6MHP and the checkpoint inhibitor ipilimumab (unpublished data). While uveitis is an expected toxicity in patients treated with ipilimumab, the adverse event was determined to be possibly related to the 6MHP because the etiology of the VKH syndrome is attributed to T cell activity against melanocytic antigens, and the 6MHP vaccine includes melanocytic antigens.

Overall, the 6MHP vaccine and adjuvants have been well-tolerated.

1.4 Montanide ISA-51

1.4.1 Rationale for Use of Montanide ISA-51

Montanide adjuvant has been effective at inducing immune responses against murine viral antigens when administered with a synthetic peptide epitope [93,94]. Montanide ISA-51 has also been used in a peptide plus GM-CSF-in-adjuvant vaccine useful for enhancing both cellular and humoral immunity [95]. The product consists of a mineral oil base comparable to incomplete Freund's adjuvant. However, the Arlacel A emulsifying agent of incomplete Freund's, which has caused reactions in the past, has been replaced with a purified mannoside monooleate called "montanide", which appears to be safer.

1.4.2 Pre-clinical Data

Pre-clinical data for Montanide ISA-51 may be referenced in the Investigator's Brochure.

1.4.3 Previous Human Experience

The UVA HITC has sponsored studies where peptide-based vaccines in Montanide ISA-51 have been safely administered to more than 500 participants. Immunological responses against the immunizing peptides have been detected in most participants. Our recent experience with the current formulation of this adjuvant is published [54,96]. Recent data support use of Montanide ISA-51 in trials with short, and long peptides, as well as with the 6MHP helper peptide vaccines (unpublished findings).

Montanide ISA-51 is produced by Seppic, Inc. (Fairfield, NJ and Paris, France). A master drug file for Montanide ISA-51 is filed with the FDA. This agent has been used in hundreds of patients in our own experience and in thousands of patients worldwide. A description of previous human experience with Montanide ISA-51 may be referenced in the Investigator's Brochure. A description of adverse events related to Montanide ISA-51 administered in combination with 6MHP may be found in Section 8.4.3.

1.5 PolyICLC

1.5.1 Rationale for Use of polyICLC

Polyinosinic-Polycytidylic acid (PolyIC) is a double-stranded RNA (dsRNA) that acts as a TLR3 agonist. However, its short half-life limits its usefulness. To increase half-life and its practical use in clinical settings, it has been stabilized with polylysine and carboxymethylcellulose as polyICLC. Like polyIC, polyICLC is a TLR3 agonist. TLR3 is expressed in the early endosome of myeloid DC; thus, polyICLC preferentially activates myeloid dendritic cells, favoring a Th1 cytotoxic T-cell response [97,98]. PolyICLC also activates NK cells and induces cytolytic potential [98].

1.5.2 Pre-Clinical Data

A description of the pre-clinical data for polyICLC may be referenced in the Investigator's Brochure.

1.5.3 Human Experience

PolyICLC has been used safely in cancer patients, with intramuscular doses up to 50 mcg/kg [99] intravenous doses up to 300 mcg/kg[100]. PolyICLC has been administered with Montanide ISA-51 plus long peptides with increased immunogenicity over Montanide ISA-51 plus peptides alone; the combination was safe, with some significant local reactions but no DLTs [101]. Our own experience with polyICLC + Montanide ISA-51in our ongoing Mel60 (NCT02126579) and Mel63 (NCT02425306) trials supports its safety and support of vaccine immunogenicity. Adverse events from the Mel63 trial are provided in Section 1.3.3.

Additional details on prior human experience with polyICLC may be referenced in the investigator's brochure for polyICLC.

1.5.4 Rationale for Dose and Regimen

We will administer 0.9 mg of polyICLC per vaccine, which represents a 10% decrease in the dose of polyICLC compared to the dose administered in the UVA-Mel60 and UVA-Mel63 protocols. The dose of polyICLC has been reduced to 0.9 mg in the proposed study because there have been changes made to the packaged "dry" weight concentration for polyICLC. PolyICLC was previously packaged at a dry weight concentration of 2 mg/ML and 0.5 mL of the 2 mg/mL formulation was used in the vaccine formulations (UVA-Mel60 and UVA-Mel63). The most current lot of polyICLC is packaged at 1.8 mg/mL dry weight of polyICLC and the intent is to keep the volume of polyICLC the same (0.5 mL) in the vaccine formulations. We do not expect the immunogenicity to be reduced with a 10% reduction in polyICLC.

1.6 CDX-1127

1.6.1 Rationale for Use of CDX-1127

The function and survival of antitumor T cells are limited by tumor-associated immune dysfunction and possibly by activation induced cell death. Activation of CD27 is crucial for T cell survival, and also has been found to decrease regulatory T cells, and thus offers promise to enhance the persistence and function of vaccine-induced T cells. Activation of CD27 supports T cell survival, through antiapoptotic signaling (Bcl-x(L) upregulation and Pim-1 induction[17]), and has a significant role in T cell memory[100]. It may recruit TRAF2, which can protect cells from TNFα-mediated apoptosis [76-78]. In mice, CD27 is critical for T cell persistence and memory [75,79]. In the clinical trial, we will assess whether CD27 agonism increases T cell response persistence and long-term memory after vaccines with short peptides, both with agonists for TLR3 and CD40, and with control adjuvants.

The impact of CD27 and CD70 on regulatory T cells (Treg) is debated. On one hand, CD70 expressed by human DCs can promote effector T cell activity and diminish Treg function in vitro[80]. On the other, murine studies suggest that CD70 is necessary for Treg development in the thymus [81] and presence and function in tumors[82], suggesting that Treg may require tonic CD70 signaling. Studies from the Bullock laboratory indicate that intra-tumoral Treg numbers are reduced by stimulating CD27, concomitant with an increase in $CD8^+$ T cell frequencies, resulting in a substantial favorable shift in the CD8:Treg ratio [18]. Particularly relevant are data from the Phase I trial of CDX-1127, where patients treated with the agonistic CD27 Ab had reduced circulating regulatory T cells by day 29 [83]. Reducing Tregs has been a therapeutic goal in cancer therapy; so, this effect would be significant if it is observed in this study, both in circulation and in the vaccine site

microenvironment. Thus, we will test whether regulatory T cells are reduced, during CDX-1127 therapy, in circulation as well as in the VSME.

1.6.2 Rationale for Dose and Regimen

In the CDX-1127-01 trial, varlilumab was well-tolerated across all doses (0.1 mg/kg – 10 mg/kg), Evidence of biologic activity, including depletion of regulatory T cells and T-cell stimulation, was also reported across all dose levels. Patients enrolled in the expansion cohort received up to 5 cycles of varlilumab: 3 mg/kg of varlilumab administered once per week for 4 weeks followed by an 8-week rest period. One patient experienced a partial response, and 8 patients experienced stable disease.

In the proposed study, we have chosen to administer varlilumab at the 3 mg/kg dose because of the reported safety profile and the reported biologic activity at this dose level. Varlilumab will be administered once every 5-6 weeks (rather than weekly), as prior data from the phase 1 study suggest that patients treated with a 4-week gap in between the first and second dose had better T cell activation profiles compared with the T cell activation profiles evaluated during the weekly dosing schedule. It is hypothesized that continuous stimulation through CD27 can lead to T cell exhaustion. Thus, a gap of more than 4 weeks in between dosing may allow for a break in stimulation through CD27 and enhanced T cell activity.

1.6.3 Pre-clinical Experience

In cynomolgus macaques, five weekly doses up to 25 mg/kg/day induced no adverse changes. Additional details are provided in the investigator brochure.

1.6.4 Previous Human Experience

Current data regarding previous human experience with CDX-1127 may be referenced in the Investigator's Brochure. A summary of findings is provided below.

The *CD27 Ab CDX-1127 (varlilumab)* has been evaluated as monotherapy in a phase I clinical trial (CDX-1127-01; NCT01460134) in 90 patients, and in combinations in 4 other trials (CDX-1127-02, 03, 04 06):

CDX-1127-02: varlilumab + nivolumab in solid tumors

CDX-1127-03: varlilumab + ipilimumab with or without CDX-1401 in melanoma

CDX-1127-04: varlilumab + sunitinib in renal cell carcinoma

CDX-1127-06: varlilumab + atezolizumab in advanced solid tumors

Data are available from the CDX-1127-01 clinical trial. Treatment-related adverse events are summarized in Table 6 and the investigator's brochure. One dose-limiting toxicity (Grade 3 hyponatremia) was reported in the dose-escalation phase of the study.

**Table 6: Treatment-related adverse events with varlilumab monotherapy**	
Treatment-related adverse event	% Rx related AEs
Fatigue	28%
Rash	14%
Nausea	12%
Decreased appetite	12%
Diarrhea	10%
Headache	8%
Vomiting	8%
Itching	8%
Fever	6%

Infusion reactions have been rare when CDX-1127 is administered as monotherapy, and have been infrequent when CDX-1127 is co-administered with checkpoint blockade antibodies. Prior infusion reactions were managed with premedication using diphenhydramine and acetaminophen.

Terminal phase half-life ranged from 6 days at 1 mg/kg dosing, to 10.6 days at 10 mg/kg.

There has been evidence of clinical activity of CDX-1127 monotherapy, with one CR in Hodgkin lymphoma (duration 33+ months), and one PR in renal cell cancer, persistent at 24.7 months. There was SD in 13 patients.

Our experience with CDX-1127 indicates it is safe and bioactive in cancer patients when delivered systemically: it increases frequencies of activated (HLA-DR^{+}) CD8+ T cells while the proportion of Tregs is decreased (NCT01460134 [20]).

1.7 <u>Human Experience with the combinations of 6MHP + polyICLC + IFA with or without CDX-1127 in the first 15 participants on the present trial</u>

Experience with DLTs in the first 15 participants on the present trial, as of 11/23/20 includes:

Arm A: 1 DLT:

1) Participant with ulceration at the vaccine site after all 6 vaccines, who developed vaccine site ulceration late.

Arm B: 3 DLTs:

1) Participant with symptomatic pneumonitis meeting DLT criteria.
2) Participant had some toxicities that led to stopping treatment after 3 vaccines (none individually met criteria for DLT, but in aggregate the general symptoms were bothersome to the patient). However, the participant later developed ulceration at the vaccine site after having had only 3 vaccines that satisfied the DLT criteria. (Note: this participant did develop a strong immune response by week 8 despite only 3 vaccines).
3) Participant with ulceration meeting DLT criteria after 4 vaccines.

1.8 Summary

This protocol is to test the safety and immunologic effects of adding systemic administration of CDX-1127 to cancer vaccines. The study design will provide data on safety of adding CDX-1127 to a vaccine regimen, and will provide preliminary data on the immunologic effects of that combination. The vaccine regimen consists of the 6MHP vaccine + Montanide ISA-51 + polyICLC.

Patients in either study arm will be randomized to receive varlilumab or not.

This clinical trial will incorporate immunologic studies of immune responses in blood during the vaccine regimen and also at 6 months, when there will be specific assessment of memory responses to each vaccine. This study will provide first-in-humans data on the impact of CD27 agonism in combination with a vaccine. The study holds promise to optimize the immunogenicity of vaccines comprising class II MHC peptides with either of two improved adjuvant systems, which may have relevance across a spectrum of cancers.

2.0 OBJECTIVES

2.1 Primary Objectives

1) Safety: To test the safety of CDX-1127 administered concurrently with a melanoma vaccine (6MHP). The melanoma vaccine will include Montanide ISA-51 and will be co-administered with polyICLC.

   Safety will be evaluated by adverse event assessments, including CTCAE v5.0 and sub-classified by irAE categories. [Endpoint: dose-limiting toxicities (DLTs) based on CTCAE v5.0 treatment related adverse events]

2) Immunogenicity: To determine whether addition of CD27 antibody to a melanoma vaccine improves the persistence of $CD4^+$ Th1 responses to vaccine antigens. [Endpoint: $CD4^+$ responses to 6MHP (by ELIspot), measured as the percent of patients with persistent responses (pRsp) at either day 127 or day 176, or both].

2.2 Secondary Objectives

1) To assess whether CD27 antibody decreases regulatory T cells ($FoxP3^+$ cells) in the vaccine site microenvironment at weeks 3 and 12. [Endpoint: in vaccine site biopsies $FoxP3^+$ $CD4^+$ T cells per mm^2 in by immunohistochemistry and gene expression analysis)] **Note:** with revision to reduce ulceration at vaccine sites, biopsies are not required at week 12 for participants whose week 12 visit would be due after IRB approval of Protocol v12-03-2020.
2) To determine whether addition of CD27 antibody to a melanoma vaccine,
   a) Decreases circulating regulatory T cells [Endpoint: circulating Tregs, % of $CD4^+$ T cells responding to vaccine antigens; $FoxP3^+$ $CD4^+$ T cells as measured by flow cytometry]
   b) Improves the frequency of $CD4^+$ Th1 responses to vaccine antigens [Endpoint: $CD4^+$ response to 6MHP measured by ELIspot; maximum increase after vaccination at any time point]
   c) Improves the frequency of durable $CD4^+$ Th1 responses to vaccine antigens (dRsp) [Endpoint: $CD4^+$ response to 6MHP measured by ELIspot; at two

consecutive time points (day 8 to 85)]

d) Increases the $CD4^+$ Th1 memory response (mRsp) to vaccine antigens at 6 months. [Endpoint: $CD4^+$ response to 6MHP by ELIspot a week after booster vaccine on day 176]

2.3 Exploratory Objectives

1) To assess the impact of CD27 antibody on induction of
   a. Antibodies to helper peptides in 6MHP (assessed in serum by ELISA for IgG). [Endpoint: IgG antibodies to 6MHP]
   b. $CD8^+$ T cell responses to melanoma antigens in the vaccine, or to other antigens by epitope spreading. [Endpoint: rate of $CD8^+$ T cell responses to peptides not in vaccine]
   c. $CD4^+$ T cell activation versus exhaustion. [Endpoint: proportion of $CD4^+$ and $CD8^+$ T cells that express PD-1, TIM-3, LAG-3, TIGIT with respect to HLA-DR as an activation marker by flow cytometry]
2) To obtain preliminary estimates of disease-free survival (DFS) and overall survival.

3.0 PARTICIPANT SELECTION CRITERIA

3.1 Inclusion Criteria

1) Either of the following patient populations may be eligible:
   a. Patients with stage IIB, IIC, III, or IV cutaneous, mucosal, or unknown primary melanoma at original diagnosis or at restaging after recurrence, rendered clinically free of disease by surgery, other therapy, or spontaneous remission, within 6 months prior to registration. Patients with high-risk stage IIA cutaneous melanoma (by DecisionDx Melanoma test, Castle Biosciences, Inc., Friendswood, TX) also may be eligible.
   b. Patients with stage IIB, IIC, III, or IV uveal melanoma at original diagnosis or at restaging after recurrence, rendered clinically free of disease by surgery, other therapy, or spontaneous remission within 6 months prior to registration. Patients with uveal melanoma treated definitively with plaque therapy may also be eligible if treated within 6 months prior to registration. Patients with high-risk stage IIA uveal melanoma (Class 2 DecisionDX-UM Melanoma test, Castle Biosciences, Inc., Friendswood, TX) also may be eligible.

2) Patients with small radiologic or clinical findings of an indeterminate nature may still be eligible: examples include a new 5 mm lung nodule that is too small to characterize or an asymptomatic 12 mm bony lucency that is not classic for malignancy, where clinical care may otherwise be to follow the patient with repeat imaging rather than to treat the lesion.

   Participants may have had cutaneous, uveal, mucosal primary melanoma, or an unknown primary melanoma. Diagnosis of melanoma must be confirmed by cytological or histological examination. Staging of cutaneous melanoma will be based on version 8 AJCC staging system (Section 9.2). Patients with primary uveal melanoma that are considered high-risk by DecisionDX-UM will not require pathologic review.

3) Participants will be required to have radiological studies to rule out radiologically evident melanoma metastasis. Required studies include:
   - Chest CT scan,

- Abdominal and pelvic CT scan, and
- Head CT scan or MRI
- PET/CT fusion scan may replace scans of the chest, abdomen, and pelvis.

4) Participants who have had brain metastases will be eligible if all of the following are true:
    - Each brain metastasis must have been completely removed by surgery or each unresected brain metastasis must have been treated with stereotactic radiosurgery.
    - No brain metastasis is > 2 cm in diameter at the time of registration.
    - Any neurologic symptoms attributable to brain metastases have returned to baseline.
    - There is no evidence of new or enlarging brain metastases.

5) The most recent surgical resections or gamma-knife therapy for malignant melanoma must have been completed ≥ 1 week and ≤ 6 months prior to registration.

6) ECOG performance status of 0 or 1 (Section 9.3).

7) Ability and willingness to give informed consent.

8) Laboratory parameters as follows:
    - ANC > 1000/mm3
    - Platelets > 100,000/mm3
    - Hgb > 9 g/dL
    - HgbA1c ≤ 8.5%
    - AST and ALT ≤ 2.5 x upper limits of normal (ULN)
    - Bilirubin ≤ 2.5 x ULN (except in patients with Gilbert's disease, where bilirubin to 4x ULN is allowed)
    - Alkaline phosphatase ≤ 2.5 x ULN
    - Creatinine ≤ 1.5 x ULN

9) Age 18 years or older at registration.

10) Participants must have at least one intact (undissected) axillary and/or inguinal lymph node basin.

3.2 Exclusion Criteria

1) Participants who have received the following medications or treatments at any time within 4 weeks of registration:
    - Chemotherapy
    - Interferon (e.g. Intron-A®)
    - Radiation therapy (Stereotactic radiotherapy, such as gamma knife, can be used ≥ 1 week and ≤ 6 months prior to registration)
    - Allergy desensitization injections
    - High doses of systemic corticosteroids, with the following qualifications and exceptions:
        - o In patients with adrenal or pituitary insufficiency replacement steroid doses are allowed; however, daily doses of 10 mg or more of prednisone (or equivalent) per day administered

parenterally or orally are not allowed in patients with normal adrenal and pituitary function.
  - o Inhaled steroids (e.g.: Advair®, Flovent®, Azmacort®) are permitted at low doses (less than 500 mcg fluticasone per day, or equivalent) [126,127].
  - o Topical, nasal, and intra-articular corticosteroids are acceptable.
- Growth factors (e.g. Procrit®, Aranesp®, Neulasta®)
- Interleukins (e.g. Proleukin®)
- Any investigational medication
- Targeted therapies specific for mutated BRAF or for MEK

2) Participants who are currently receiving nitrosoureas or who have received this therapy within 6 weeks of registration.

3) Participants who are currently receiving a checkpoint molecule blockade therapy, or who have received this therapy within 12 weeks of registration.

4) Participants with known or suspected allergies to any component of the vaccine.

5) Participants who were vaccinated previously with 6MHP are excluded. Participants who have received vaccinations containing agents other than the synthetic peptides included in this protocol and have recurred during or after administration of the vaccine will be eligible to enroll 12 weeks following their last vaccination.

6) Participants who have previously received CDX-1127 or other CD27 agonistic antibody.

7) Pregnancy. Female participants of childbearing potential must have a negative pregnancy test (urinary or serum beta-HCG) obtained within 2 weeks prior to registration. Males and females must agree, in the consent form, to use effective birth control methods during the course of active study treatment and for up to 70 days after the last dose of study treatment.

8) HIV positivity or evidence of active Hepatitis C virus (testing to be done within 6 months of study entry).

9) Female participants must not be breastfeeding.

10) Participants in whom there is a medical contraindication or potential problem in complying with the requirements of the protocol in the opinion of the investigator.

11) Participants classified according to the New York Heart Association classification as having Class III or IV heart disease (Section 9.4).

12) Participants with uncontrolled diabetes, defined as having an HgbA1c > 8.5%.

13) Participants must not have had prior autoimmune disorders requiring cytotoxic or immunosuppressive therapy, or autoimmune disorders with visceral involvement. Participants with an active autoimmune disorder requiring these therapies are also excluded. The following will not be exclusionary:
    - The presence of laboratory evidence of autoimmune disease (e.g. positive ANA titer) without symptoms
    - Clinical evidence of vitiligo
    - Other forms of depigmenting illness
    - Mild arthritis requiring NSAID medications

- Resolved childhood asthma/atopy
- Intermittent use of bronchodilators or local steroid injections
- Hypothyroidism stable on hormone
- Replacement for Addison's disease

14) Participants with known addiction to alcohol or drugs who are actively taking those agents, or participants with recent (within 1 year) or ongoing illicit IV drug use.

15) Participants who have received a live vaccine within 30 days of registration.

16) Body weight < 110 pounds at registration, due to the amount and frequency with which blood will be drawn.

17) Participants with prior autoimmune pneumonitis.

3.3 Registration and Randomization

All participants must sign the consent form prior to determination of eligibility for this study. All participants who meet the inclusion/exclusion criteria may be registered. Registration will occur following verification of eligibility by the treating physician. Participants should receive their first study treatment within 3 weeks of registration.

When a site is ready to enroll a patient, the following documentation must be scanned and emailed to the UVA Coordinating Center (UVA CC):

- Patient and staff signed informed consent form (ICF)
- Completed Inclusion/Exclusion checklist demonstrating participant eligibility
- Supporting documentation needed to confirm eligibility (lab results, scan results etc.)

Consult the Manual of Operations for instructions on sending this information. The Coordinating Center will consult with the Sponsor if questions arise in confirming eligibility. The UVA CC will communicate the participant number and treatment dose assignment to the enrolling site.

Registration will occur following verification of eligibility by the treating physician.

Participants who are consented and accrued to the study should be registered in OnCore in accordance with the Clinical Trial Management System Policy via the UVa OnCore Resources link in Oncore. General guidelines are available in the OnCore User Manual and Data Entry Guide.

Treatment randomization will be discussed with participants during the process of informed consent. Treatment randomization will be based upon the study design as shown in Figure 1, until a safety bound has been triggered or target accrual has been met. The randomization sequence will be generated by the study statisticians. Randomization will occur after registration within 1 week of the start of treatment.

This study does not involve any blinding or masking procedures. Participants will be told the treatment they are receiving.

4.0 STUDY DRUGS

4.1 6MHP Vaccine

4.1.1 Peptide Synthesis

The vaccine drug product 6MHP to be administered consists of 6 peptides. All peptides were synthesized directly from amino acids by Multiple Peptide Systems (now Polypeptide Group, San Diego, CA) under GMP conditions. Recombinant vectors in bacteria or viruses were not used. The synthetic peptides were purified by HPLC. The identity of the synthetic peptides has been confirmed by verifying their mass and amino acid sequences by mass spectrometry. Details of the synthesis, certificates of analysis, and technical summaries may be cross-referenced in IND 12191 and may be found in the chemistry and manufacturing section of the IND submission (IND 10825).

4.1.2 Storage of Individual Peptides

Each bulk peptide was supplied to the HITC as lyophilized powder without excipients and stored at a temperature ≤ -70°C and protected from light.

4.1.3 Reconstitution and Vialing of the Vaccine

Lyophilized peptides were reconstituted, mixed and vialed under GMP conditions or under good laboratory practices (GLP) conditions in a clean lab room. Details of the vialing may be cross-referenced in IND12191 and may be found in the chemistry and manufacturing section of the IND submission (IND 10825).

The sponsor is providing peptides to each site.

4.1.4 Vaccine Storage

The vials of lyophilized peptide are stored at a temperature ≤ -70°C and protected from light. Once thawed, the vial(s) must be used for preparation of the vaccine within 24 hours.

4.1.5 Lot Testing

Each lot of peptide vaccine is evaluated as required by the FDA for identity, sterility, general safety, purity, and pyrogenicity. The details of these tests may be cross-referenced in IND12191 and may be found in the chemistry and manufacturing section of the IND submission (IND 10825).

4.1.6 Stability testing

The peptide vaccine will undergo stability testing yearly.

4.1.7 Packaging and Labeling

A vial of lyophilized vaccine contains 300 mcg of each peptide.

Each vial of lyophilized peptide is labeled with the following information:

- Short name of the product

- Product number
- Proper name of the product
- Name and address of the vialing facility
- Lot number
- Date of manufacture (the date of vialing the reconstituted peptides)
- Serial number
- Quantity of each peptide per vial
- Vial contains no preservative, store at ≤ -70°C
- "Caution: New Drug – Limited by US Federal law to investigational use"

4.1.8 Adjuvants

Class II MHC-restricted melanoma peptides (6-MHP; 200 mcg) in aqueous solution are combined with Hiltonol/polyICLC then mixed 1/1 with Montanide ISA-51 to form water-in-oil emulsions (see separate documentation for vaccine mixing instructions).

4.1.9 Packaging and Labeling

Montanide ISA-51 consists of a mineral oil base similar to incomplete Freund's adjuvant (IFA). However, the Arlacel A emulsifying agent of incomplete Freund's, which has caused reactions in the past, has been replaced with a purified mannide monooleate called "montanide", which appears to be safer. Montanide ISA-51 is provided as a GMP-grade sterile liquid in single use 3-mL vials (or similar size).

Details on packaging and labeling may be found in the drug master file for Montanide ISA-51. A letter to cross-reference the drug master file is included with the IND submission (IND 10825).

4.1.10 Storage

The Montanide ISA-51 is stored at room temperature.

4.1.11 Supply

Montanide ISA-51 is manufactured by Seppic, Inc. (Fairfield, NJ).

The sponsor will provide Montanide ISA-51 to each site.

4.2 POLYICLC (HILTONOL)

4.2.1 Packaging and Labeling

PolyICLC (Hiltonol) is provided for use as an adjuvant as a clinical grade reagent in single-use vials containing 1 mL of a 1.8 mg/mL solution (1.8 mg/mL "dry" polyIC concentration).

Details on packaging and labeling may be found in the drug master file for polyICLC. A letter to cross-reference the drug master file is included with the IND submission (IND 10825).

4.2.2 <u>Storage</u>

The polyICLC (Hiltonol) is stored at 2-8°C.

4.2.3 <u>Supply</u>

PolyICLC (Hiltonol) is manufactured by Oncovir, Inc. (Washington, D.C).

The Sponsor will provide polyICLC to each site.

4.3 CDX-1127 (Varlilumab)

The information below was taken from the IB for CDX-1127.

4.3.1 <u>Packaging and Labeling</u>

CDX-1127 Drug Product is formulated as a clear, colorless sterile solution intended for single-use parenteral administration. Each vial contains a nominal 5.0 mg/mL CDX-1127 protein in a 10 mL volume of buffered solution composed of Sodium Phosphate, Potassium Phosphate, Potassium Chloride, Sodium Chloride, and Polysorbate 80 with a pH of 7.0.

Varlilumab will be labeled according to the requirements of local law and legislation.

4.3.2 <u>Storage</u>

- CDX-1127 drug product is shipped in insulated shippers and will be stored at 2 - 8°C (36 - 46°F) until use. Celldex Therapeutics, Inc. will be contacted if temperature excursions occur.

- CDX-1127 will be protected from light. However, sufficient light protection is provided by the secondary container (carton); no specific light protection is needed during preparation of the dosing solution and infusion.

- CDX-1127 is not formulated with a preservative. Therefore, once the sterile vials are entered (i.e., once CDX-1127 is drawn into a syringe), the drug will be used as soon as possible (within 4 hours or in accordance with any applicable institutional guidance).

- Additional guidance regarding storage and handling of varlilumab will be provided within a pharmacy summary sheet provided by Celldex.

4.3.3 <u>Supply</u>

CDX-1127 is manufactured by Celldex Therapeutics, Inc.

Celldex Therapeutics, Inc. will provide varlilumab to each site.

4.3.4 <u>Preparation</u>

The varlilumab dose to be administered will be diluted to a final volume of 90 ml for infusion, according to the instructions provided by Celldex in the pharmacy

summary sheet. No dilution is necessary in cases where the drug volume is greater than 90 ml.

4.4 Study Drug Accountability

Study drug should only be used for the purposes of this study protocol. All study drugs will be stored in a secure area at each participating site. Study drug accountability will be maintained by the sites.

Any unused vials of 6MHP, poly-ICLC or Montanide ISA-51 will either be returned to the Sponsor at the Sponsor's request or will be destroyed in accordance with institutional policies, as directed by the Sponsor.

Any unused vials of CDX-1127 will either be returned to Celldex Therapeutics, Inc. at their request or will be destroyed in accordance with institutional policies, as directed by Celldex Therapeutics, Inc.

5.0 TREATMENT PLAN

5.1 Management of Participants

This study will be conducted on an outpatient basis, with participants scheduled to be evaluated as needed for clinical care, and as specified in the study calendar (Section 9.1) through 6 months (or more often if needed for testing or medical reasons). Participants will be off treatment follow-up at about 8 months, or when another therapy is initiated, whichever occurs first. Once off treatment follow-up, participants will be followed yearly for progression-free survival and overall survival.

5.2 Administration of 6MHP, Montanide ISA-51, and polyICLC

5.2.1 Overview

The peptides will be prepared by combining the 6MHP mixture of 6 peptides, which are provided as 300 mcg (0.3mg) of each peptide per vial with Montanide ISA-51 or polyICLC and Montanide ISA-51. More information is provided below, for each study arm. Details of making emulsions with Montanide ISA-51 are provided in the mixing instructions.

In the initial study design, subjects received 6MHP + polyICLC in an emulsion of Montanide ISA-51 adjuvant on days 1, 8, 15, 36, 57 and 78. With approval of Protocol v12-03-2020, vaccines on days 57 and 78 will not be administered, and subjects will receive 6MHP + polyICLC in an emulsion of Montanide ISA-51 adjuvant on days 1, 8, 15, and 36.

Subjects will receive 6MHP in an emulsion of Montanide ISA-51 adjuvant as part of a booster vaccine on day 176.

In the initial study design, each vaccine was administered subcutaneously (50%) and intradermally (50%) at one skin location. After approval of Protocol v12-03-2020, each vaccine will be administered subcutaneously (100%) at one skin location. The same skin location will be used for all vaccines. If the vaccine site has severe inflammation or ulceration, the next vaccine may be placed near the original site.

On days when both varlilumab and vaccine are administered, the vaccine may be administered before or after varlilumab, but preferably before.

5.2.2 Dose Calculations

Days 1, 8, 15, 36 (and for days 57 and 78 before approval of Protocol v12-03-2020):

- At each designated time-point, 200 mcg each of the 6 peptides (Table 1) plus 0.9 mg of polyICLC will be emulsified in Montanide ISA-51 adjuvant (1:1 ratio). A total of 2 mL will be administered.

Day 176:

- 200 mcg each of the 6 peptides (Table 1) will be emulsified in Montanide ISA-51 adjuvant (1:1 ratio). A total of 2 mL will be administered.

Pre-medications

None required, though oral antihistamines (eg: loratadine), acetaminophen, or NSAIDs may be administered for symptom management.

If patients have pain at the injection site, EMLA ® cream may be administered topically before the vaccine is administered. This will be considered on a case by case basis as lidocaine may induce immunosuppression.

5.2.3 Preparation of Study Drug

Directions on how to prepare the investigational drug will be provided in vaccine mixing sheets. Vaccines will be stored at room temperature until the vaccine is administered. Ideally, the vaccine should be administered 1-2 hours after mixing. If the vaccine is not administered within 4 hours after mixing, it should be discarded.

5.2.4 Post-Vaccine Observation

All participants will be closely observed for adverse events for at least 20 minutes following each vaccination. Any time thereafter, participants should report any adverse events to the research coordinator or research clinician.

5.3 Administration of Varlilumab (CDX-1127)

Information taken from IB for varlilumab.

5.3.1 Overview

Subjects in Arm A will receive three doses of CDX-1127(3 mg/kg)

- Day 1
- Day 36
- Day 78

The study drug will be administered intravenously over a 90-minute time period.

CDX-1127 will be administered as an intravenous infusion in accordance with the applicable study protocol. CDX-1127 should not be administered as a bolus injection.

5.3.2 Dose Calculations

A participant's dose of CDX-1127 will be calculated based on their actual body weight at the time of screening. A participant's dose of CDX-1127 will remain constant at each time point, unless they experience a greater than 10% change in body weight.

Body Weight(kg) x Dose of CDX-1127(3 mg/kg) = Volume of CDX-1127(mL)
5mg/mL

5.3.3 Pre-medications

Prophylactic premedication will not be given initially; however, if a patient experiences a grade 2 infusion related reaction, that patient should be pre-medicated for subsequent infusions with diphenhydramine 50 mg (or equivalent) and/or 500 to 750 mg paracetamol (acetaminophen) at least 30 minutes prior to CDX-1127 administrations. Should 2 or more patients develop ≥ Grade 2 symptoms, these prophylactic pre-medications are recommended for future infusions in all subsequent patients. Prophylactic premedication with corticosteroids may also be added if clinically indicated.

5.3.4 Investigational Drug Precautions

- Celldex Therapeutics, Inc. recommends wearing a laboratory coat and gloves when dispensing and administering the study drug.
- Avoid mixing the investigational agent with other drugs in the infusion bag or the administration set.

5.3.5 Preparation of Study Drug

- CDX-1127 will be diluted to a final volume of 90 mL for infusion. Directions on how to prepare the investigational drug will be provided in a pharmacy summary sheet provided by Celldex Therapeutics, Inc.
- CDX-1127 will be administered as a 90-minute ±10 minute IV infusion using an infusion pump with a 0.22 micron-in-line filter.

5.3.6 Post-Infusion Observation

- Participants will be observed for 1-hour post-infusion. Vitals should be collected at the end of the observation period.

5.4 Dose Modifications

5.4.1 6MHP, Montanide ISA-51, and polyICLC

- There will be no dose modifications of vaccine components.

5.4.2 Varlilumab

- A participant's dose of CDX-1127 will be calculated based on their actual body weight at the time of screening, and the dose will remain constant at each time point, unless they experience a greater than 10% change in body weight. No other changes in the dose of investigational drug are permitted.

5.5 Dose Delays

5.5.1 Dose Delays Due to Toxicity

- Dose delays for vaccines and CDX-1127

  In circumstances where assessment of an AE is limited, such as by intercurrent illness, or when laboratory studies are required to assess for other causes of toxicity, the vaccine* and/or CDX-1127 schedule may be interrupted for up to 21 days.

  - If a toxicity can be clearly attributed to either the vaccine or CDX-1127, then only the investigational drug that caused the toxicity will be delayed.
  - One vaccine and/or one CDX-1127 delay up to 21 days is allowed if due to an AE, regardless of attribution.
  - If there is more than one vaccine and/or one CDX-1127 delay by 7 days or more due to an AE, regardless of attribution, the investigational drug that caused the toxicity should be discontinued. The remaining investigational drug may be continued.
  - Missed doses may be shifted. Participants who are treated outside of the established schedule should return to the original schedule as soon as possible, keeping in mind that there must be at least a minimum of 6 days in between CDX-1127 infusions and a minimum of 5 days in between vaccines.

  *The vaccine includes all components that make up the vaccine (e.g.6MHP + Montanide ISA-51 with or without).

5.5.2 Delayed Visits for Reasons Other Than Toxicity

A schedule for return visits should be established at the first visit. If a participant misses a treatment, the missed treatment will be administered as soon as possible, so that subsequent treatments will be given in the appropriate intervals. Treatment may be continued for an additional time period, if needed. Participants who are treated outside of the established schedule should return to the original schedule as soon as possible keeping in mind that there must be at least a minimum of 6 days in between CDX-1127 infusions and a minimum of 5 days in between vaccines.

Note: Labs and study treatment/procedures may occur on different days as long as each occurs within the specified established window.

Table 7 defines what constitutes a delayed visit, whether the participant should continue to be treated, and whether a protocol violation should be reported and recorded. The range of days is counted from the original scheduled date.

Table 7

Delayed Visit for Reasons other than Toxicity			
**Treatment Period**	**Range of Days[a]**	**Participant Treatment**	**Protocol Deviation**
Vaccine 1/Varli[c]			
Day 1	± 2 days	Vaccine/Labs/Varli	No
	± 3 to 7 days	Vaccine/Labs/Varli	Yes
	± 8 or more days	Labs	Yes
Vaccine 2			
Day 8	± 2 days	Vaccine	No
	± 3 to 7 days	Vaccine	Yes
	± 8 or more days	none	Yes
Vaccine 3			
Day 15	± 2 days	Vaccine/Labs	No
	± 3 to 7 days	Vaccine/Labs	Yes
	± 8 or more days	Labs	Yes
Assessment/Biopsy			
Day 22	± 2 days	Biopsy/Labs	No
	± 3 to 7 days	Biopsy/Labs	Yes
	± 8 or more days	Labs	Yes
Vaccine 4/Varli[c]			
Day 36	± 7 days	Vaccine/Varli/Labs	No
	± 8 to 14 days	Vaccine/Varli/Labs	Yes
	± 15 or more days	Labs	Yes
Vaccine 5			
Day 57	± 7 days	Vaccine/Labs before Protocol v12-03-2020. Labs only after approval of Protocol v12-03-2020.	No
	± 8 to 14 days	Vaccine/Labs before Protocol v12-03-2020. Labs only after approval of Protocol v12-03-2020.	Yes
	± 15 or more days	Labs	Yes
Vaccine 6/Varli[c]			
Day 78	± 7 days	Vaccine/Varli/Labs before Protocol v12-03-2020. Varli/Labs after approval of Protocol v12-03-2020.	No
	± 8 to 14 days	Vaccine/Varli/Labs before Protocol v12-03-2020. Varli/Labs after approval of Protocol v12-03-2020.	Yes
	± 15 or more days	Labs	Yes
Assessment/Biopsy			
Week 12 (day 85)	± 7 days	Labs/Biopsy/Scans before Protocol v12-03-2020.	No

Delayed Visit for Reasons other than Toxicity			
**Treatment Period**	**Range of Days[a]**	**Participant Treatment**	**Protocol Deviation**
		Labs/Scans after approval of Protocol v12-03-2020.	
	± 8 to 14 days	Labs/Biopsy/Scans before Protocol v12-03-2020. Labs/Scans after approval of Protocol v12-03-2020.	Yes
	± 15 or more days	Labs	Yes
Week 18 (day 127)	± 7 days	Labs	No
	± 8 to 14 days	Labs	Yes
	± 15 or more days	Labs	Yes
Booster vaccine			
Week 25 (day 176)	± 7 days	Labs/Booster vaccine	No
	± 8 to 30 days	Labs/Booster vaccine	Yes
Week 26 (day 183)	± 2 days[d]	Scans[b]/Labs	No
	± 3 or more days[d]	Scans[b]/Labs	Yes
Discontinuation (if applicable)			
EOT 1	-	-	-
EOT 2	± 2 days	Labs	No
	± 3 or more days	Labs	Yes
Safety Follow-Up: 30 days post discontinuation (FUV1)			
	± 7 days	Phone call	No
	± 8 to 30 days	Phone call	Yes

[a] A participant will be taken off protocol treatment if more than one vaccination is delayed [3 days or more] during the treatment period.
[b] Scans are required for participants who complete the vaccine sequence and who are scheduled to complete day 183.
[c] Arm A only.
[d] If the day 176 visit is delayed, the day 183 visit needs to be 7 days later than the day 176 visit. This range of days applies around the 7 day interval.

5.6 Discontinuation of Therapy

Protocol treatment will be discontinued for any of the following reasons:

- Any dose-limiting toxicity as defined in Section 8.9.
- Disease progression requiring other therapy (e.g. surgery under general anesthesia, radiation, chemotherapy, or steroid therapy). The appearance of small metastases or recurrent tumor deposits will not be a basis for discontinuing the study treatment. Biopsy to determine the nature of new lesions or minor surgical procedures to excise a new lesion (with or without anesthesia) will not be a basis for discontinuing study treatment.
- Initiation of cytotoxic chemotherapy, radiation therapy, surgery for resection of disease, steroid therapy, or other immunosuppressive therapy.
- Any other potential adverse reaction deemed sufficiently serious to warrant discontinuation of therapy by the Principal Investigator or one of the Associate Investigators.

- Noncompliance with the requirements of the study.
- Therapy may be discontinued at the participant's request.
- Therapy may be discontinued at the discretion of an Investigator.
- Pregnancy. Pregnant participants will continue to be followed for the duration of the pregnancy.

Participants who discontinue treatment will be followed according to the follow-up schedule outlined in Section 9.1, unless a participant has withdrawn consent.

5.7 Replacement of Study Participants

A participant who is enrolled but does not receive study drug or does not undergo any of the study related procedures may be replaced. Every attempt will be made to evaluate any data from these participants for endpoint assessment.

5.8 Concomitant Medications

Medications taken in the month prior to registration will be recorded on the baseline case report form. This includes prescription medications, over-the-counter medications, injected medications, biological products, blood products, imported drugs, or street drugs. Participants should be maintained on drugs that they were taking prior to entry unless a change in regimen is medically indicated.

The following are non-permitted medications or treatments

- Cytotoxic chemotherapy
- Interferon therapy (e.g. Intron-A®)
- Radiation therapy
- Nitrosoureas
- Allergy desensitization injections
- Corticosteroids, as detailed in Section 3.2, with exceptions in Section 5.10.
- Growth factors (e.g. Procrit®, Aranesp®, Neulasta®)
- Interleukins (e.g. Proleukin®)
- Antibodies to PD-1 or other immune checkpoint blockade therapies (e.g. Keytruda®)
- Other investigational medications
- Street drugs
- Targeted therapies specific for mutated BRAF or for MEK

5.9 Permitted Medications or Treatments

- Nonsteroidal anti-inflammatory agents
- Anti-histamines (e.g. Claritin®, Allegra®)
- Topical corticosteroids (refer to Exclusion Criteria for permitted steroids)
- Steroid treatment for infusion reactions
- Replacement steroids for adrenal insufficiency
- Short-term therapy for acute conditions not specifically related to melanoma
- Chronic medications except those listed in Section 5.8
- Non-live vaccines, including non-live influenza vaccines, are permitted, but should be administered at least 2 weeks prior to or at least 2 weeks after a

study drug. Live vaccines should not be administered within 30 days of registration or within 30 days after the last dose of study drug.

5.10 Supportive Care Guidelines for CDX-1127

CDX-1127 is expected to have agonistic properties only in conjunction with antigen specific immune responses, i.e., modulation of T cell responses by CDX-1127 is expected to require additional signals occurring in the context of ongoing immune responses. CDX-1127 is not expected to have “superagonist” properties as was observed with the TeGenero anti-CD28 monoclonal antibody, i.e., induction of massive T cell activation occurring independent of antigen specific immune responses that resulted in cytokine storm and life-threatening adverse events.

The agonist properties of CDX-1127 could theoretically promote the growth of hematological malignancies that express CD27, although the data available do not support this possibility (please refer to section 4.2.9 of the investigator’s brochure).

Immune-related adverse events (irAE) have been observed following administration of immunomodulatory monoclonal antibodies, in particular, ipilimumab, and it is possible that CDX-1127 may also induce such events. An irAE has been defined as a clinically significant adverse event of any organ that is associated with study drug exposure, of unknown etiology, and is consistent with an immune-mediated mechanism. Serologic, immunologic, and histologic (biopsy) data should be used to support an irAE diagnosis. Appropriate efforts should be made to rule out neoplastic, infectious, metabolic, toxin, or other etiologic causes of the adverse event. Most commonly irAEs involve skin, GI tract, endocrine organs and liver, but can involve any organ system.

Cytokine Mediated Acute Syndromes/Infusion Reactions

Since CDX-1127 contains only human protein sequences, it is unlikely to be immunogenic and induce a hypersensitivity reaction. It is possible that CDX-1127 could induce a cytokine release syndrome or cytokine storm that could manifest as an infusion reaction occurring during or shortly after completion of the infusion. If such a reaction were to occur, it might manifest with fever, chills, rigors, headache, rash, pruritus, arthralgia, hypo- or hypertension, bronchospasm, change in mental status, or other symptoms. Severe reactions may result in renal failure, coma, disseminated intravascular coagulation or other life-threatening symptoms and death. Treatment of an infusion reaction is discussed below and includes slowing, interruption or discontinuation of the infusion with or without supportive care. **Any infusion reaction ≥ Grade 3 should be reported to Celldex or designee, immediately but no later than 24 hours of UVA CC awareness of the event.** A serum sample should be obtained as soon as possible following the onset of any suspected case of cytokine release syndrome. The serum should immediately be stored at ≤ -20°C.

Infusion reactions should be graded according to NCI-CTCAE (Version 5.0) guidelines, http://ctep.cancer.gov. Treatment recommendations are provided below and may be modified based on local treatment standards and guidelines and investigator judgment, as appropriate. Medications to treat hypersensitivity are available at the outpatient site when CDX-1127 is being administered.

For Grade 1 symptoms: (Mild transient reaction; infusion interruption not indicated; intervention not indicated)

- Remain at bedside and monitor subject until recovery from symptoms.

For Grade 2 symptoms: (infusion interruption indicated but responds promptly to symptomatic treatment [e.g., antihistamines, NSAIDS, narcotics, corticosteroids, i.v. fluids]; prophylactic medications indicated for ≤ 24 hours)

- Stop the CDX-1127 infusion, begin an i.v. infusion of normal saline, and treat the subject with diphenhydramine 50 mg i.v. (or equivalent) and/or 500 to 750 mg paracetamol/acetaminophen; remain at bedside and monitor subject until resolution of symptoms. Corticosteroid therapy may also be administered per investigator judgment. If the infusion is interrupted, then restart the infusion at 50% of the original infusion rate when symptoms resolve; if no further complications ensue after 30 minutes, the rate may be increased to 100% of the original infusion rate. Monitor subject closely. If symptoms recur then no further CDX-1127 will be administered at that visit. The amount of study drug infused must be recorded on the case report form (CRF). Subjects who experience an adverse event, including an infusion reaction of Grade 2, during the post-infusion observation period that does not resolve during this time should be observed for 24 hours or until the adverse event resolves with vital sign measurements every 4 hours and additional evaluations as medically indicated for the management of the adverse event.

For Grade 3 or Grade 4 symptoms: (Grade 3: prolonged [i.e., not rapidly responsive to symptomatic medication and/or brief interruption of infusion]; recurrence of symptoms following initial improvement; hospitalization indicated for other clinical sequelae [e.g., renal impairment, pulmonary infiltrates]. Grade 4: life-threatening consequences; urgent intervention indicated).

- Immediately discontinue infusion of CDX-1127. Begin an i.v. infusion of normal saline, and treat the subject -per investigator judgment. Subjects who experience an adverse event, including an infusion reaction of Grade ≥ 3, regardless of resolution, may be observed for up to 24 additional hours, based on the investigator's judgment. Restarting the infusion, and subsequent retreatment will be at the discretion of the investigator.

  In the case of late-occurring hypersensitivity symptoms (e.g., appearance of a localized or generalized pruritus within 1 week after treatment), symptomatic treatment may be given (e.g., oral antihistamine, or corticosteroids).

  Investigators may follow their institutional guidelines for the treatment of anaphylaxis.

<u>Immune-related Adverse Events (irAE)</u>

It is possible that syndromes may develop that are most consistent with an underlying enhanced immune response as the driving factor. Such events most commonly consist of rash, diarrhea and colitis, autoimmune hepatitis, and endocrinopathies (primary hypopituitarism caused by hypophysitis, hypo- or hyperthyroidism, and adrenal insufficiency), but may also cause arthritis, glomerulonephritis, thrombocytopenia and involve multiple organ systems. Experience with previous immunomodulatory mAbs indicates that irAEs are typically low grade and self-limited and more often occur after multiple doses. However, some irAEs may be severe and life threatening.

Colitis, characterized by diarrhea and which may be accompanied by abdominal pain and/or GI bleeding, is of particular concern based on experience with ipilimumab. This

irAE can rapidly worsen from low to high grade and result in gastrointestinal perforation and death. Events of Grade 3 or Grade 4 diarrhea as well as Grade 2 diarrhea with blood in stool should be evaluated for colitis. **Any ≥ Grade 2 irAE, such as diarrhea/colitis or liver enzyme elevation, must be reported to Celldex, or designee, within 24 hours of UVA CC awareness of the event.**

Other Possible Adverse Events

Tumor lysis syndrome: It is possible that via direct Fc mediated effector mechanisms, CDX-1127 may result in tumor lysis syndrome. This may be particularly germane for CD27 expressing hematologic tumors. Rapid lysis of tumors may result in asymptomatic laboratory abnormalities to clinical changes secondary to electrolyte disturbances, including cardiac arrhythmias, neuromuscular irritability, tetany, seizures, and mental status changes.

Proliferation of CD27-expressing tumors: CD27 is a signaling molecule that is involved in the survival and proliferation of normal lymphocytes. Although CD27 signaling requires concomitant T cell receptor stimulation to promote T cell activation and proliferation, it is conceivable that in tumor cells CD27 signaling alone may lead to growth promoting effects. The nonclinical data with a limited number of human tumor cell lines and primary tumors suggest that CDX-1127 does not promote the growth of CD27 expressing tumors.

5.11 Management of Toxicity

The study will be monitored continuously for treatment-related adverse events. Expected treatment-related toxicities of 6MHP combined with IFA and/or polyICLC will be managed in accordance with Section 5.5.1, which allows for dose delays, but not dose reductions.

Please consult the Investigator Brochure for CDX-1127 for additional information on irAEs and guidelines for treatment.

5.12 Treatment Compliance

Treatment compliance may be evaluated through drug accountability assessments and through the evaluation of subject medical records and CRF documents.

5.13 Biopsies

Vaccine Site Biopsies
Before Protocol v12-03-2020, each participant had a biopsy of a vaccine site at two time points (days 22 and 85 or End of Treatment). After approval of Protocol v12-03-2020, only the biopsy at day 22 will be done. The biopsy will consist of three 4-mm punch biopsies of skin.

Prior to Protocol v12-03-2020, the vaccine site biopsy at day 85 was required for all participants, except that it was optional for participants who discontinued vaccine therapy before vaccine 6 but did not discontinue varlilumab therapy if applicable, and it should not have been done if the participant had ulceration at the vaccine site or had a vaccine-site related DLT.

Tumor Biopsy
Optional at the time of recurrence or later as clinically indicated.

6.0 CLINICAL AND LABORATORY EVALUATIONS

The following evaluations will be performed on an outpatient basis. Please refer to Section 9.1 for scheduling.

6.1 Physical Exams and Evaluations

- Medical History
- Complete Physical Exam (includes weight, performance status, medication review, neurologic function-general)
- Vital signs (includes temperature, pulse, respiratory rate, and blood pressure)
- Assessment of skin and nodal basins for evidence of disease recurrence or metastasis
- Assessment of skin for vitiligo
- Assessment of hair and eye color
- Designation of vaccination sites (at screening only)
- Height (baseline only)
- Assessment of baseline symptoms (baseline only)

6.2 Pathology Review

- Review of pathology at the enrolling institution

6.3 Performance Status

- ECOG performance status criteria will be used in the evaluations (Section 9.3).

6.4 Clinical Labs

Laboratory tests for hematology, chemistry, urinalysis, and other are specified in Table 8. Pre-dose laboratory procedures can be conducted up to 72 hours prior to dosing. Results must be reviewed by the investigator or qualified designee and found to be acceptable prior to each dose of varlilumab. Any abnormal laboratory values captured which are not included in Table 8 but are considered to be pertinent positive clinical signs/symptoms, and laboratory results obtained as part of routine care of patients will be recorded in the UVA Cancer Center database and reported as described in Section 8.6.1. If there is any doubt on the part of study personnel concerning what constitutes a pertinent positive finding, the PI and sponsor will be consulted.

Table 8: Clinical Labs			
**Hematology**	**Chemistry**	**Urinalysis**	**Other**
Hematocrit	Albumin	Blood	Serum β-human chorionic gonadotropin (β-hCG)[1]
Hemoglobin	Alkaline phosphatase	Glucose	ANA
Platelet count	Alanine aminotransferase (ALT)	Protein	Rf
WBC (total and differential)	Aspartate aminotransferase (AST)	Specific gravity	
Red Blood Cell Count	Carbon Dioxide *(CO_2 or bicarbonate)*	Microscopic exam *(If abnormal)*	Blood for correlative studies: Section 6.9.
Absolute Neutrophil Count	Calcium	Urine pregnancy test[1]	HIV[2]
Absolute Lymphocyte Count	Chloride		HCV[2]
Absolute Eosinophil Count	Creatinine		HGB-A1C
	Glucose		
	Potassium		
	Sodium		
	Total Bilirubin		
	Direct Bilirubin		
	Total protein		
	Blood Urea Nitrogen		

[1] Perform on women of childbearing potential only. If urine pregnancy results cannot be confirmed as negative, a serum pregnancy test will be required.

[2] Antibody screen; reflexive testing to determine whether active disease is present.

6.5 Toxicity Assessments

- Assessment of adverse events. The NCI Common Terminology Criteria for Adverse Events (CTCAE) version 5.0 will be used for the characterization and grading of adverse events.
- Toxicity diaries will be distributed to participants and reviewed by study personnel.

6.6 Research Blood Samples

Blood should be obtained prior to any drug therapy and prior to a vaccine injection if a vaccine is scheduled to be administered on that date. Results of research blood tests are not required prior to administering the vaccine on that date.

The following blood samples for research will be collected and processed.

Lymphocytes: Green Top Tubes		
Day	Amount	Purpose
Day 1	120 cc	HLA typing and immunologic testing described in Section 6.9
All other visits	80 cc	Immunologic testing described in Section 6.9
Serum: Red Top Tubes		
Day 1 and Day 57	20 cc	Immunologic testing described in Section 6.9
All other visits	20 cc	Immunologic testing described in Section 6.9

6.7 Vaccine Site Biopsies

6.7.1 Sampling

Prior to Protocol v12-03-2020, each participant had a biopsy of a vaccine site at two time points (days 22 and 85). After approval of Protocol v12-03-2020, each participant will undergo a biopsy at a vaccine site at day 22 only. The biopsy will consist of three 4-mm punch biopsies of skin. The biopsy will be performed on skin at the site of the most recent vaccine.

6.7.2 Procedure

Three 4-mm punch biopsies will be obtained under local anesthesia. As each is removed, the specimens will be placed into the following:

1) liquid nitrogen (1 specimen),
2) formalin (1 specimen),
3) RNA-later (1 specimen)

6.7.3 Evaluations

The skin of the vaccine sites may be evaluated for immune activation and cellular infiltrates using multiple assays, including, but not limited to:

1) Liquid Nitrogen/Quick frozen tissue: Luminex or other protein assays for cytokines and chemokines
2) Formalin-fixed paraffin-embedded tissue: Immunofluorescence or immunohistochemistry for infiltration by immune cells (e.g.: CD8, CD4, dendritic cells), for Th1/Th2/Th17/Treg bias (T-bet, GATA3, RORγt, FoxP3), and other immune related markers as needed.
3) RNA-later: Gene expression profiling of vaccine sites for immune signatures.

6.8 Tumor Biopsies (Optional, at the time of progression)

6.8.1 Size Requirements and Sampling

The size of tissue saved for research will depend on the available tissue. Ideally it will be at least 0.3 cm^3.

6.8.2 Procedure

If during the study, participants develop metastases or recurrences, or progress, these may be removed, and following receipt by pathology, may be evaluated by the study research team.

When appropriate (and we anticipate the majority of cases) the biopsies will be performed under local anesthesia (typically lidocaine HCl 1% and epinephrine 1:100,000 injection + or - 8.4% sodium bicarbonate), in the outpatient clinic or comparable procedure room, using sterile technique. In cases when clinical standard of care requires a larger procedure the biopsies may be performed in the operating room under standard technique.

To minimize errors in analysis due to sampling error and specimen heterogeneity, each study biopsy specimen will be divided into several components and randomly allocated into various preservation conditions. Ideally, tissue will be divided into the following preservation conditions, using core needle biopsies (19 mm long and 2 mm diameter; about 80 mm3), or incisional or excisional biopsies with at least the same minimum tissue volume.

It is most valuable to obtain the following:

- Formalin: 1 core biopsy or similar tissue volume (about 80 mm^3 or greater) will be fixed in formalin, then paraffin-embedded (for histology/immunohistology)
- Quick-frozen: 2 core biopsies or similar tissue volume (each about 80 mm^3 or greater) quick-frozen processed for protein studies, histology, or nucleic acid studies. If only one core can be obtained, this portion should be provided as two specimens (e.g. cut the core biopsy specimen in half).

When sufficient tissue is available, the following should also be obtained:

- Viable cell suspension: 2 core biopsies or similar tissue volume (total about 160 mm^3 or greater) will be processed for single-cell suspension by mechanical disaggregation, then enzymatic digestion (collagenase, hyaluronidase, DNAase). The resulting suspensions will be cryopreserved in FBS serum and DMSO (for cellular immune function and flow cytometry).
- RNA-later: 1 core biopsy or similar tissue volume (about 80 mm^3 or greater) will be placed in RNA-later (for RNA/RT PCR)
- If there is additional tissue, it may be processed for additional immunologic studies.

6.8.3 Evaluations

Tissue samples may be screened for antigen expression or protein profiles using tests such as Western blots, immunohistochemistry, PCR, flow cytometry or gene chip analysis. Tumor escape mechanisms may also be evaluated. Specimens will be used in immunological assays to assess T cell infiltration, T cell function or antibody response. Assays generally used for this type of testing include, but are not limited to, immunohistochemistry, flow cytometric analyses, T cell receptor sequencing, ELIspot assays, ELISAs, chromium-release assays, proliferation assays and intracellular cytokine staining. Specimens may be used to study the

immunologic aspects of the tumor microenvironment or as targets or controls in laboratory assays. Specimens may be used to establish cell lines for long-term studies.

This tissue may also be compared to lesions resected prior to enrollment, which will be requested from the pathology department of each institution as paraffin-embedded tissue samples, and these tissues may be banked for use in future studies. If participants are removed from the study or progress during or after follow-up, tissue may be collected as part of clinical care or for research. The tissue may be collected under this study or under a separate tissue banking study (if patients are removed from the current study). This tissue may be analyzed as described above or banked for use in future studies.

6.9 Assessments

6.9.1 Anti-tumor Activity

Anti-tumor activity will be assessed by delay in melanoma recurrence, by physical examination and routine imaging:

Tumor Imaging

Tumor imaging may include CT/PET-CT scans and/or MRI. These will complement physical exam and other imaging as required, but the primary assessments will be based on CT/PET-CT and/or MRI. For each participant, the same method of assessment should be used at baseline and throughout the course of the study.

Tumor Measurements

Patients enrolled on this trial are not expected to have measurable disease.

6.9.2 Immunologic Assessments

Assessments of T cell function may include, but are not limited to the following:

- ELIspot assays
- ELISAs
- Chromium-release assays
- Proliferation assays
- Intracellular cytokine staining
- T cell receptor sequencing.
- Cytokine bead array
- Flow cytometry
- HLA typing

Characterization of cellular populations may include, but are not limited to the following:

- Immunohistochemistry
- Gene expression analysis
- Flow cytometry
- ELISAs

- Western-blot analysis
- Intracellular cytokine staining
- Cytokine bead array
- T cell receptor analysis

6.10 Study Calendar

See Section 9.1.

7.0 STATISTICAL CONSIDERATIONS

This is an early phase randomized trial to obtain preliminary data on the safety and immunogenicity of CDX-1127 (CD27 antibody) administered concurrently with a melanoma vaccine (6MHP). The primary goals are to assess the safety of CDX-1127 when administered with vaccine; and to obtain preliminary estimates of the difference in immune outcome measures between participants randomized to 6MHP + CDX-1127 (Arm A) versus 6MHP (Arm B). Exploratory goals are to obtain preliminary data on the induction of antibodies; $CD8^+$ T cell response to antigens; cell activation and exhaustion; and clinical outcomes. Eligible participants will be accrued and randomized with equal allocation to one of two treatment arms as shown in Table 9.

Table 9: Arms
A: 6MHP + Montanide ISA-51 + polyICLC + CDX-1127 (6MHP + CDX-1127)
B: 6MHP + Montanide ISA-51 + polyICLC (6MHP)

7.1 Evaluation of Sample Populations and Endpoints

7.1.1 Safety

All participants receiving any protocol defined treatment will be evaluated for safety.

The 6MHP vaccine has been safe, with lower toxicities than with class I peptides, in particular with lower autoimmune or hyperimmune toxicities. Data from MEL63 indicates a DLT rate of 6.3% with a 95% CI(1, 18%). Reported DLT information (ref: Burris et al JCO May 2) for CDX-1127 in patients with advanced solid tumors indicated a DLT rate of 4% with a 95% CI(<1, 20%). We do not anticipate increased toxicities by combining 6MHP vaccine with CDX-1127 therefore, this trial will be designed with stopping rules for DLT rates beyond those expected with vaccine alone or CDX-1127 alone. Formal safety bounds based upon monitoring dose limiting toxicities (DLT) (Section 8.9) will guide decisions about early stopping due to potential safety concerns. If a stopping bound is crossed then accrual to the study will be suspended until the study PI, co-investigators and the DSMC can review the data, and determine if the study should continue, be amended or be closed to further accrual.

7.1.2 Immunogenicity

All eligible participants receiving any protocol defined treatment who have at least two evaluable blood draws (including baseline) for immune analyses will be evaluated for the following immune response parameters. In addition, all

participants are considered ‘evaluable’ for the primary endpoint if they have been evaluated for immune response at baseline and at least day 127; or they have progressed prior to the day 127 assessment; or have stopped treatment due to DLTs.

In PBMC:

- $CD4^+$ Th1 response to vaccine antigens by ELIspot:
    - o Magnitude of response at all times
    - o Frequency of response over time
    - o Persistence of response over time
- Circulating regulatory T cells (Tregs) as measured by flow cytometry over time
- $CD8^+$ T cell responses to peptides not in the vaccine over time
- $CD4^+$ T cell activation versus exhaustion
    - o Activation measured by an increase in %DR^+ $CD4^+$ cells of total $CD4^+$ cells
    - o Exhaustion measured by increase in the % of DR^+CD4^+ T cells that co-express both PD-1 and either LAG3 or TIM3.

In the tumor microenvironment:

- $FoxP3^+$ $CD4^+$ T cells per mm^2 in vaccine site biopsies by immunohistochemistry and gene expression analysis at weeks 3 and 12. **Note:** with revision to reduce ulceration at vaccine sites, biopsies are not required at week 12 for participants whose week 12 visit would be due after IRB approval of Protocol v12-03-2020.

In serum:

- IgG antibodies to 6MHP by ELISA for IgG over time

A $CD4^+$ T cell response to 6MHP (Rsp), is defined as all of the following, based on direct ELIspot assay:

a) an increase of at least 20 IFN-gamma secreting cells per 100,000 $CD4^+$ T cells, over the maximum of the negative controls ($T_{neg\ cntrl}$)
b) no overlap in standard deviations between the response to 6MHP peptide pool (T_{vax}) and $T_{neg\ cntrl}$
c) the ratio of T_{vax} to $T_{neg\ cntrl}$ at one or more time points, after week 0, is at least 5-fold the prevaccine ratio of T_{vax} to $T_{neg\ cntrl}$. Note that any prevaccine ratio of T_{vax} to $T_{neg\ cntrl}$ below 1 will be set to 1 to avoid overinflating that ratio.

A durable response (dRsp) is defined as a Rsp detected over at least two consecutive time periods during vaccination (days 8 through 85).

A persistent response (pRsp) is defined as a dRsp plus a Rsp detected on day 127 or 176.

A memory recall (mRsp) is defined as a Rsp detected at any time point from day 8 through day 85, and a Rsp on day 183 compared to the Rsp on day 176, with all of the following:

a) an increase of at least 20 IFN-gamma secreting cells per 100,000 $CD4^+$ T cells over $T_{neg\ cntrl}$ at day 183.
b) no overlap in standard deviations between T_{vax} and $T_{neg\ cntrl}$ at day 183.

d) at least a 2-fold increase in the ratio of T_{vax} to $T_{neg\ cntrl}$ compared to the ratio of T_{vax} to $T_{neg\ cntrl}$ at day 176. Note that if that ratio at day 176 is below 1, it will be set to 1 to avoid overinflating response assess`ent at day 183.

Evaluable participants who have progressed prior to the day 127 assessment or have stopped treatment due to DLTs will be considered response failures for dRsp, pRsp and mRsp.

Measures of $CD8^+$ T cell responses by ELIspot will use the same criteria defined above, except that that definition of response will be based on response to peptides restricted by Class I MHC molecules, and the data will be normalized to the number of $CD8^+$ T cells in the assays (eg: criterion of at least 20 IFN-gamma secreting cells per 100,000 $CD8^+$ T cells over $T_{neg\ cntrl}$).

7.1.3 Clinical

All eligible participants receiving any protocol defined treatment will be evaluated for clinical response to treatment as defined by the following measures.

- Disease-free survival (DFS), defined as the time from the date of randomization to the date of progression or death from any cause, whichever occurs first. A participant who dies without a reported progression will be considered an event on the date of death. Participants who have neither progressed nor died will be censored on the date of last evaluable tumor assessment.
- Overall survival (OS), defined as the time from the date of randomization to the date of death from any cause. Participants who do not experience an event (death) will be censored on the date of last follow-up/contact.

7.2 Sample Size and Accrual

Target sample size was based on the number of participants that could be accrued within the funding amount. This was set at 30 evaluable participants.

A primary endpoint for this study is a persistent response (pRsp) to vaccine antigens (see section 7.1.2). Results from Mel63 for the 38 evaluable participants treated on Arms C & D (6MHP + Montanide ISA-51 + polyICLC without & with mCy) resulted in 5 participants with a pRsp (13.1%). The 90% CI for this estimate is (5.3% to 25.7%) resulting in a half-width of 10.2%. For this study, an observed difference in the persistent response rates in the range of 28% to 31% (approximately 3 times the half-width) is needed to support that the addition of CDX-1127 to 6MHP vaccine warrants further study. This determination is based upon having positive lower and upper 90% CI bounds for the difference in pRsp for study Arm B – Arm A assuming Arm A pRsp rates of 13.1% to 25.7% using the score method of Miettinen and Nurminen.

Maximum target sample size is estimated to be 34 participants, which assumes a 10% dropout/ineligibility/lost to follow-up adjustment. Accrual is estimated at approximately 20 participants per year, therefore, accrual to the study should be completed in two years with final analysis occurring 9-12 months later.

7.3 Randomization

Participants will be randomized 1:1 to treatment arms A and B with random block sizes of 2 and 4.

7.4 Safety Monitoring

Toxicities will be monitored using CTCAE 5.0 criteria, with additional recording of immune-related adverse events (irAE). Stopping rules will be based on expected DLT rates discussed in Section 7.1.1 for each of the combination arms. Significant increases over the reported rates are not expected but would be a reason for early discontinuation. Thus, formal safety bounds based upon the observed number of participants who experience a DLT as defined in Section 8.9 will guide decisions about early stopping due to potential safety concerns. If a stopping bound is crossed then accrual to the study will be suspended until the study PI, co-investigators, sponsors and the DSMC can review the data, and determine if the study should continue, be amended or be closed to further accrual. The rate of DLTs will be monitored up to 30 days after the last vaccination. A sequential probability ratio test (SPRT) based upon a binomial test of proportions for DLTs will be used. Only the upper boundary will be used for monitoring to protect against excessive failure rates. The stopping boundary are for a SPRT contrasting a 5% versus 25% DLT rate, with nominal type I and II errors of 10% and 10%, respectively.

Stopping guidelines have triggered two study reviews and amendments. Upon approval of treatment change in Protocol v12-03-2020, the monitoring stopping guidelines will be applied separately to the subset of participant's accrued pre and post approval of Protocol v12-03-2020.

Table 10: Stopping Guidelines for DLTs

Number of participants pre and post approval of Protocol v12-03-2020	Boundary
2-6	≥ 2
7-14	≥ 3
15-16	≥ 4

7.5 Analyses

Study populations and evaluation criteria are noted in Section 7.1. Adverse events will be summarized by frequency and magnitude of event. Incidence of DLTs will be monitored against the safety bounds above. The bounds are non-binding, and provide a guideline that may result in study modification or closure.

A 5-fold or greater CD4+ Th1 memory response to vaccine antigens compared to baseline is consider a response. Amounts less than 5-fold are considered as a non-response. Immune and clinical endpoints are defined in Section 7.1.2 and in Section 7.1.3, respectively. Time to event measures (DFS and OS) will be estimated by the methods of Kaplan and Meier.

This study is designed to estimate differences and not to make statistical comparisons between arms. All $CD4^+$ T cell and CD8+ T cell ELIspot results will be assessed relative to the participant specific baseline measure with graphical representation of measures over time. The difference in the proportion of participants considered to have a durable response between arms will be estimated and a 90% confidence interval around the difference, using the score method of Miettinen and Nurminen. Similarly, the difference in

the proportion of participants considered to have a memory response at day 183 between arms will be estimated and a 90% confidence interval around the difference calculated. In addition, repeated measure models will be explored to estimate difference in patterns of response between arms over time for CD4+ T cell, CD8+ T cell and percent of circulating Tregs (with a log10 transformation). Within an arm, for each participant, the difference in amount of $FoxP3^{+}$ cells in the vaccine site microenvironment at weeks 3 and 12 (for those with biopsies done on week 12) will be calculated. Estimated difference in mean change by arm with 90% CI estimates around the difference in means will be used to estimate potential differences by arm.

8.0 ADVERSE EVENT DATA COLLECTION AND MONITORING

8.1 Definitions

8.1.1 Adverse event (AE)

Any unfavorable and unintended sign (including an abnormal laboratory finding), symptom, or disease temporally associated with the use of a medical treatment or procedure regardless of whether it is considered related to the medical treatment or procedure (attribution of unrelated, unlikely, possible, probable, or definite). Medical conditions or diseases present before starting the investigational drug will be considered AEs if they worsen after starting study treatment.

8.1.2 Unexpected AE or unexpected suspected adverse reaction. (21 CFR 312.32)

An adverse event or suspected adverse reaction is considered “unexpected” if it is not listed in the investigator brochure or is not listed at the specificity or severity that has been observed; or, if an investigator brochure is not required or available, is not consistent with the risk information described in the general investigational plan or elsewhere in the current application, as amended. “Unexpected,” as used in this definition, also refers to adverse events or suspected adverse reactions that are mentioned in the investigator brochure as occurring with a class of drugs or as anticipated from the pharmacological properties of the drug, but are not specifically mentioned as occurring with the particular drug under investigation.

8.1.3 Serious AE –

Any adverse drug experience occurring at any dose that results in any of the following outcomes:

- Death;
- A life-threatening adverse drug experience;
- Inpatient hospitalization, or prolongation of existing hospitalization (as defined below in this section);
- A persistent or significant disability/incapacity; or a congenital anomaly/birth defect.
- Important medical events that may not result in death, be life threatening, or require hospitalization may be considered a serious adverse drug experience when, based upon medical judgment, they may jeopardize the patient or participant and may require medical or

surgical intervention to prevent one of the outcomes listed in this definition.

- Hospitalization for expedited AE reporting purposes is defined as an inpatient hospital stay equal to or greater than 24 hours. Hospitalization is used as an indicator of the seriousness of the adverse event and should be reserved for situations where the adverse event truly fits this definition and not for hospitalizations associated with less serious events. For example, the following are not considered serious adverse events:
    - a hospital visit where a patient is admitted for observation or minor treatment (e.g. hydration) and released in less than 24 hours
    - hospitalization for pharmacokinetic sampling
    - admission to hospice
    - hospitalizations planned before entry into the clinical study
    - hospitalization for elective treatments
    - hospitalizations to work up Grade 1 adverse events

8.1.4 **<u>Unanticipated problem –</u>**

An unanticipated problem is any event/experience that meets ALL 3 criteria below:

- Is unexpected in terms of nature, severity or frequency given the research procedures that are described in the protocol-related documents AND in the characteristics of the participant population being studied.
- Is related or possibly related to participation in research. This means that there is a reasonable possibility that the incident may have been caused by the procedures involved in the research study.
- The incident suggests that the research placed the participant or others at greater risk of harm than was previously known or recognized OR results in actual harm to the participant or others.

8.1.5 **<u>Protocol Deviation-</u>**

A protocol deviation is defined as any change, deviation, or departure from the study design or procedures of a research project that is NOT approved by the institution's IRB prior to its initiation or implementation, OR deviation from standard operating procedures, Good Clinical Practices (GCPs), federal, state or local regulations. Protocol violations may or may not be under the control of the study team or UVA staff. These protocol violations may be major or minor violations.

8.1.6 **<u>Suspected Adverse Reaction (as defined in 21 CFR 312.32 (a))-</u>**

Any adverse event for which there is a reasonable possibility that the drug caused the adverse event. For the purposes of IND safety reporting, "reasonable possibility" means there is evidence to suggest a causal relationship between the drug and the adverse event. Suspected adverse reaction implies a lesser degree of certainty about causality than adverse reaction, which means any adverse event caused by a drug.

8.2 Attribution Assessment

8.2.1 Attribution –

The determination of whether an adverse event is related to a medical treatment or procedure. The attribution groups are:

Definite – Applies to those adverse events which, the investigator feels are incontrovertibly related to study drug. An adverse event may be assigned an attribution of definitely related if or when (must have all of the following):

- It follows a reasonable temporal sequence from administration of the test drug.
- It could not be reasonably explained by the known characteristics of the participant's clinical state, environmental or toxic factors, or other modes of therapy administered to the participant.
- It disappears or decreases on cessation or reduction in dose with re-exposure to drug. (Note: This is not to be constructed as requiring re-exposure of the participant; however, the group of definitely related can only be used when a recurrence is observed.)
- It follows a known pattern of response to the test drug.

Probable – Applies to those adverse events for which, after careful consideration at the time they are evaluated, are felt with a high degree of certainty to be related to the test drug. An adverse event may be considered probably related if or when (must have three of the following):

- It follows a reasonable temporal sequence from administration of the test drug.
- It could not be reasonably explained by the known characteristics of the participant's clinical state, environmental or toxic factors, or other modes of therapy administered to the participant.
- It disappears or decreases on cessation or reduction in dose. There are important exceptions when an adverse event does not disappear upon discontinuation of the drug, yet drug-relatedness clearly exists (e.g. bone marrow depression, fixed drug eruptions, tardive dyskinesia).
- It follows a known pattern of response to the test drug.

Possible – Applies to those adverse events for which, after careful consideration at the time they are evaluated, a connection with the test drug administration appears unlikely but cannot be ruled out with certainty. An adverse event may be considered possibly related if or when (must have two of the following):

- It follows a reasonable temporal sequence from administration of the test drug.
- It could not readily have been produced by the participant's clinical state, environmental or toxic factors, or other modes of therapy administered to the participant.
- It follows a known pattern of response to the test drug.

Unlikely – Applies to those adverse events for which, after careful consideration at the time they are evaluated, are judged to be unrelated to the test drug. An adverse event may be considered unlikely if or when (must have two of the following):

- It does not follow a reasonable temporal sequence from administration of the test drug.
- It could readily have been produced by the participant's clinical state, environmental or toxic factors, or other modes of therapy administered to the participant.
- It does not follow a known pattern of response to the test drug.
- It does not reappear or worsen when the drug is re-administered.

Unrelated – Applies to those adverse events, which after careful consideration, are clearly and incontrovertibly due to extraneous causes (disease, environment, etc.).

8.3 Data collection

Data will be collected using a centralized electronic case report form in **ON**-line **C**linical **O**ncology **R**esearch **E**nvironment = **Oncore**.

8.4 Risks and Safety

8.4.1 Adverse Event Descriptions and Grading Scales

The NCI Common Terminology Criteria for Adverse Events (CTCAE) version 5.0 will be used for the characterization and grading of adverse events.

8.4.2 Time Span for Reporting Adverse Events

Reporting of AEs will begin when the participant is first administered the study drug or has a study related procedure. Events occurring through 30 days after administration of the last dose of vaccine or varlilumab, regardless of attribution, will be reported. Participants are to be contacted 30 days after receiving study drug to obtain adverse event data. This may be done by phone, mail, email, or in person. For patients who discontinue protocol therapy prior to day 176, they should be contacted 30 days after the last vaccine or varlilumab administration for completion of AE data collection.

AEs should be followed to resolution or stabilization. If an AE worsens and becomes an SAE, it should be reported as serious per the guidelines specified for SAE reporting.

AEs that are possibly, probably, or definitely related to any of the study drugs will be recorded until the participant completes treatment follow-up. If, during treatment follow-up, the participant receives an alternative anti-cancer treatment, participants will be off treatment follow-up and will be followed yearly for disease progression and survival.

8.4.3 Agent-Specific Adverse Events List:

PolyICLC

Prior overall clinical experience and a summary of adverse events for polyICLC are provided in Sections 4 and 5 of the investigator's brochure.

Additional data for 45 patients treated with 20 mcg/kg 3x/week are summarized in Table 11. These doses equate to about 1.4 mg per dose and 4.2 mg per week (much higher than the dose that will be used in this protocol).

Table 11: Adverse Events (treatment-related) for PolyICLC

Category	Toxicity	Grade	Comment
Nervous system disorders	Headache	Grade 2	1 of 22 (<5%)
	Tremors	Grade 3	2 of 45 (4%) poss related
Musculoskeletal and connective tissue disorders	Muscle weakness	Grade 2	1 of 45 (<2%) experienced at Grade 3
Respiratory, thoracic and mediastinal disorders	Dyspnea	Grade 2	1 of 45 (<2%) experienced at Grade 3
	Hypoxia	Grade 2	1 of 45 (<2%) experienced at Grade 3
Metabolism and nutrition disorders	Hypernatremia	Grade 2	1 of 45 (<2%) experienced at Grade 3
Investigations	Elevated transaminases (GPT)	Grade 3	4 of 45 (9%) 3 cases possibly related; one case probably related. Typically transient.
	Elevated Alkaline phosphatase	Grade 3	7% of patients, in IB
	Leukocytopenia	Grade 3	2 of 45 (<4%) in published work; 20% in IB
	Thrombocytopenia	Grade 3	14% of patients in IB
	Neutropenia	Grade 3	10% of patients in IB
Blood/lymph disorders	Anemia	Grade 3	13-31% of patients in IB
General disorders and administration site conditions	Vaccine site reaction	Grade 2	1 of 22 (<5%)
	Fever	Grade 3	14% of patients in RCC trial
	Chills	Grade 3	10% of patients in RCC trial
	Fatigue	Grade 3	10% of patients in RCC trial

This higher dosage of polyICLC induced grade 3 toxicities in a subset of patients. However, in a trial of a peptide vaccine administered in an emulsion with polyICLC and Montanide ISA-51, there were no grade 3 toxicities. For that cohort of 11 patients, the following grade 2 toxicities were reported: injection site reaction (27%), panniculitis (1/11 = 9%)(46). In that trial they administered 1.4 mg of polyICLC with each dose, every 3 weeks x 5 (total 7 mg over 12 weeks). We will administer 0.9 mg of polyICLC with each dose. Thus, we expect toxicities from polyICLC in this trial to be similar to those in that peptide vaccine trial with the exception of panniculitis, which we have not observed in our ongoing experience with polyICLC.

Table 12 includes adverse drug reactions for polyICLC that are considered expected for the purposes of expedited reporting.

Table 12: Adverse drug reactions expected for polyICLC		
	Grade 2	Grade 3
Vaccination Complication[a]	+	+[b]
Skin Induration	+	
Skin Ulceration	+	+[c]
Fatigue	+	

[a] Note: In prior studies, vaccine related toxicities were captured under the injection site reaction category. Using CTCAE v5.0, vaccine related toxicities will be captured under vaccine complications. Skin induration and skin ulceration will continue to be captured under their respective categories.

[b]Vaccination complication with necrosis, erythema ≤ 20 cm, and/or induration/swelling ≤ 20 cm is expected. Vaccination complication with severe pain or limiting self-care ADL is not expected. Other grade 3 vaccination complications are not expected.

[c] Grade 3 skin ulceration at a vaccine site is expected as long as no ulcer exceeds 2 cm in greatest dimension

<u>6MHP + Montanide + polyICLC</u>

Adverse events specific for the 6MHP vaccine administered in an emulsion with Montanide ISA-51 with or without polyICLC and with or without metronomic cyclophosphamide (mCy) are provided in Table 13 and Table 14.

Table 13: Adverse events (treatment-related) for 6MHP + Montanide ISA-51 +polyICLC and +/- oral metronomic cyclophosphamide					
	6MHP + Mont + polyICLC (Mel63 n=6)		6MHP + Mont + polyICLC + mCy (Mel63 n=28)		
	Grade		Grade		
**Toxicity (based on max grade)**	**1**	**2**	**1**	**2**	**3**
Maximum overall		6 (100%)	6 (21%)	20 (71%)	2 (7%)
LOCAL, INJECTION SITE					
Injection site reaction		6 (100%)	12 (43%)	16 (57%)	
Skin induration	2 (33%)	4 (67%)	12 (43%)	16 (57%)	
Ulceration	1 (17%)		2 (7%)		1 (4%)
CONSTITUTIONAL					
Fatigue	4 (67%)		16 (57%)	2 (7%)	
Headache	3 (50%)		4 (14%)		
Rigors, Chills	2 (33%)		5 (18%)		
Nausea	4 (67%)		4 (14%)	1 (4%)	
Myalgias	2 (33%)		3 (11%)	1 (4%)	
Arthralgias			4 (14%)	1 (4%)	
Fever			2 (7%)	1 (4%)	
Dizziness			1 (4%)		

Table 13: Adverse events (treatment-related) for 6MHP + Montanide ISA-51 +polyICLC and +/- oral metronomic cyclophosphamide					
	6MHP + Mont + polyICLC (Mel63 n=6)		6MHP + Mont + polyICLC + mCy (Mel63 n=28)		
	Grade		Grade		
**Toxicity (based on max grade)**	**1**	**2**	**1**	**2**	**3**
Anorexia			2 (7%)		
Diarrhea			1 (4%)		
Cough			2 (7%)		
Pain larynx/throat			2 (7%)		
Flushing	1 (17%)				
Hot flashes			2 (7%)		
Vomiting	1 (17%)				
Flu-like syndrome	1 (17%)		5 (18%)		
Hyperhidrosis	1 (17%)				
Mucositis			1 (4%)		
Constipation			1 (4%)		
Autoimmune reaction			1 (4%)		
Wound complication				1 (4%)	
Pain, other			1 (4%)		
Bruising			1 (4%)		
Other	1 (17%)				
CLINICAL LABORATORY					
Lymphopenia				3 (11%)	1 (4%)
Blood/lymphatic (other)			1 (4%)		

Table 14: Adverse events (treatment-related) for 6MHP + Montanide ISA-51				
	6MHP + Mont (Mel41, E1602, and Mel44 n= 209 [40,46,47])			
	Grade			
**Toxicity (based on max grade)**	**1**	**2**	**3**	**4**
LOCAL, INJECTION SITE				
Injection site reaction	18%	56%	2.4%	
Skin induration				
Ulceration		5%	1.4%	
CONSTITUTIONAL				
Fatigue	43%	8%	3.4%	
Headache	27%	1.4%	0.5%	
Rigors, Chills	24%	1.4%		
Nausea	23%	2%	0.5%	
Sweating	19%	1%		
Myalgias	18%	0.5%		
Arthralgias	17%	0.5%		
Fever	16%	2%		

**Table 14: Adverse events (treatment-related) for 6MHP + Montanide ISA-51**				
	**6MHP + Mont (Mel41, E1602, and Mel44 n= 209 [40,46,47])**			
	**Grade**			
**Toxicity (based on max grade)**	**1**	**2**	**3**	**4**
Dizziness	13%			
Anorexia	13%	3%		
Diarrhea	12%	2%		
Cough	13%			
Allergic rhinitis	11%			
Nasal/paranasal reactions	11%			
Pain larynx/throat	10%			
Flushing	10%			
Hot flashes				
Pruritis	9%	0.5%		
Rash	6%	3.4%		
Dyspnea	5%	1%	0.5%	
Vomiting	5%	1%	0.5%	
Flu-like syndrome	6%			
Hyperhidrosis				
Mucositis	6%			
Constipation	5%			
Autoimmune reaction	4%	0.5%		
Wound, non-infectious	4%			
Pain, other	2%	1%	0.5%	
Abdominal pain	1.4%		0.5%	
Tinnitus		0.5%	0.5%	
Tumor Pain	0.5%		0.5%	
Hearing (without monitoring program)			0.5%	
Bruising				
Other				
CLINICAL LABORATORY				
Hyperglycemia (not fasting)	22%	1%		
Hemoglobin, low	17%	1%	0.5%	
Hyperkalemia	13%			
Lymphopenia	9%	2.9%	1%	
Leukocytes	8%	1%		
Hyponatremia	7%			
Increased creatinine	6%	0.5%		
Hypoglycemia	6%			
AST, SGOT	5%			0.5%
ALT, SGPT	4%		1%	
Neutrophils	3%	2%		
Blood/lymphatic (other)				
Metabolic, Other	3%	0.5%	0.5%	

Table 14: Adverse events (treatment-related) for 6MHP + Montanide ISA-51				
	6MHP + Mont (Mel41, E1602, and Mel44 n= 209 [40,46,47])			
	Grade			
**Toxicity (based on max grade)**	**1**	**2**	**3**	**4**
Alk phos	4%	0.5%		

Included are events reported in 4% or more of participants or events with one or more grade 3-4 treatment-related AEs. *One patient had a grade 4 ALT elevation (<0.5%)

Table 15 includes adverse drug reactions that are considered expected for the purposes of expedited reporting.

Table 15: Adverse drug reactions expected for 6MHP (>4%-grade 2 or higher)		
	**Grade 2**	**Grade 3**
Vaccination Complication[a]	+	+[b]
Skin Induration	+	
Skin Ulceration	+	+[c]
Fatigue	+	

[a] Note: In prior studies, vaccine related toxicities were captured under the injection site reaction category. Using CTCAE v5.0, vaccine related toxicities will be captured under vaccine complications. Skin induration and skin ulceration will continue to be captured under their respective categories.

[b] Vaccination complication with necrosis, erythema ≤ 20 cm, and/or induration/swelling ≤ 20 cm is expected. Vaccination complication with severe pain or limiting self-care ADL is not expected. Other grade 3 vaccination complications are not expected.

[c] Grade 3 skin ulceration at a vaccine site is expected as long as no ulcer exceeds 2 cm in greatest dimension

<u>CDX-1127 (Varlilumab)</u>

Adverse events for CDX-1127 are described in the investigator's brochure.

Adverse drug reactions that are considered expected for the purposes of expedited reporting may be found in section 6.3 of the investigator's brochure.

<u>Expected toxicities from vaccine site biopsies.</u>

Below is a list of expected AEs related to vaccine site biopsies:

<u>Vaccine Site Biopsies</u>

- Bleeding
- Bruising
- Pain
- Very low risk of infection (less than 2%)
- Delayed wound healing
- Scarring

Below is a list of expected AEs related to tumor biopsies:

Adverse events expected from tumor biopsies

- Bleeding
- Bruising
- Pain
- Infection
- delayed wound healing
- scarring
- numbness

8.5 Adverse Event Classifications

Adverse events (AEs) are classified into sections, specified in the CTCAE v5.0. For specific classifications pertaining to the protocol, we specify the following:

Hematologic/Metabolic- Any AE coded under one of the CTCAE v5.0 categories listed in Table 16 should be reported under the Hematologic/Metabolic adverse event classification:

**Table 16: Hematologic/Metabolic Classifications**	
Section	AE
Blood and lymphatic	Anemia<br>Leukocytosis
Investigations	**ALL EXCEPT:**<br>Carbon monoxide diffusing capacity decreased<br>Ejection fraction decreased<br>Forced expiratory volume decreased<br>Vital capacity abnormal<br>Weight gain<br>Weight loss
Metabolism and nutrition disorders	**ALL EXCEPT:**<br>Alcohol intolerance<br>Anorexia<br>Dehydration<br>Glucose intolerance<br>Iron overload<br>Obesity<br>Tumor lysis syndrome

Non-hematologic/Non-Metabolic- Any AE not reported under hematologic/metabolic, ocular, or allergic/autoimmune, should be reported under the non-hematologic/non-metabolic adverse event classification.

Ocular – Any AE coded under eye disorders in CTCAE v5.0 Adverse Event Terms should be reported under the Ocular adverse event classification:

Allergic/Autoimmune – Only AEs coded as Immune System Disorder: Allergic reaction, autoimmune disorder, or anaphylaxis should be reported under the Allergic/Autoimmune

adverse event classification. Other AEs coded under Immune System Disorder should be reported under Non-hematologic/Non-metabolic adverse event classification.

8.6 Reporting Adverse Events

8.6.1 Process for Reporting AEs:

Dose-limiting toxicities

A DLT must be reported to the UVA Coordinating Center (UVA CC/ Sponsor) within 24 hours of being aware of the event, and associated CRFs must be completed. UVA will report the DLT to Celldex within 24 hours of being aware of the event. The DLT should be entered into OnCore within 5 calendar days of the study team learning of the event. DLT's that are deemed serious and unexpected will be submitted to the IRB per institutional guidelines (see below).

Other AEs

AEs must be recorded into the University of Virginia Cancer Center OnCore database per the following guidelines in Table 17.

Table 17: AE Reporting in OnCore

High Risk Studies
Reporting requirements for AEs that occur within 30 days of the last dose of protocol specified treatment

	Grade 1	Grade 2		Grade 3				Grade 4 & 5
	Expected and unexpected	Expected	Unexpected	Expected		Unexpected		Expected and Unexpected
				Without hospitalization	With hospitalization	Without hospitalization	With hospitalization	
Unrelated Unlikely	OnCore 30 days[a]	OnCore 30 days	OnCore 30 days	OnCore 30 days	OnCore 15 days	OnCore 30 days	OnCore 15 days	OnCore 7 days
Possible Probable Definite	OnCore 30 days[a]	OnCore 30 days	OnCore 15 days	OnCore 30 days	OnCore 15 days	OnCore 7 days	OnCore 7 days	OnCore (24-hrs)* 7 days

*Enter into OnCore database within 24 hours if unexpected and definitely related to protocol specified treatment
Hospitalization defined as an inpatient hospital stay or prolongation of a hospital stay equal to or greater than 24 hours
[a] Grade 1 unexpected or expected hematologic/metabolic events will be recorded in the Cancer Center Database; however, regardless of attribution, these events do not have to be reported.

8.6.2 Serious Adverse Event Reporting

The study clinician will report to the sponsor any serious adverse event, whether or not considered study intervention related, including those listed in the protocol or investigator brochure and must include an assessment of whether there is a reasonable possibility that the study intervention caused the event. Study endpoints that are serious adverse events (e.g. all-cause mortality) must be reported in accordance with the protocol unless there is evidence suggesting a causal relationship between the study intervention and the event (e.g., death from anaphylaxis). In that case, the investigator must immediately report the event to the sponsor.

All serious adverse events (SAEs) will be followed until satisfactory resolution or until the site investigator deems the event to be chronic or the participant is stable. Other supporting documentation of the event may be requested by the UVA Coordinating Center (UVA CC) and should be provided as soon as possible.

Site Reporting Requirements
Any Event Resulting in death that is deemed DEFINITELY related to (caused by) study participation

or

Any event deemed serious

- Report to the UVA CC within 24 hours from the time the study team received knowledge of the event according to UVA CC requirements.
- Report to your IRB in accordance with your IRB guidelines.

  (For sites that use the UVA IRB-HSR as the IRB of record, the UVA CC will report to the UVA IRB-HSR—see sponsor reporting requirements)

Sponsor Reporting Requirements
Note: The UVA CC may submit on behalf of the sponsor.

- All safety reports sent to the FDA will also be sent to Celldex Therapeutics.
- All SAEs occurring through at least 30 days post-treatment should be reported to Celldex by the UVA CC. Any SAE that occurs following this period may be submitted to Celldex at the UVA Investigator's discretion. The principal investigator will inform Celldex in writing using a CIOMS form or MEDWATCH 3500A form of any SAE within 24 hours of being aware of the event (see Section 8.1.3 for the definition of SAE). The written report must be completed and supplied to Celldex by facsimile or email. The initial report must be as complete as possible, including an assessment of the causal relationship between the event and the investigational product(s), if available. Information not available at the time of the initial report (e.g., an end date for the adverse event or laboratory values received after the report) must be documented on a follow-up report.

  Completed SAE reports are to be submitted to:

  Celldex Therapeutics, Inc.
  Pharmacovigilance
  Fax No: 781-644-6434
  Email: SAE@celldex.com (Note: check for removal of HIPAA identifiers prior to sending information).
  For questions regarding SAE reporting: 908-323-2233 (SAE Hotline)

- The UVA CC is responsible for notifying the UVA IRB-HSR of any event resulting in death that is deemed DEFINITELY related to (caused by) study

within 24 hours from the time the study team received knowledge of the event. Report using IRB Online and by telephone.

- The UVA CC is responsible for notifying the Food and Drug Administration (FDA) of any unexpected fatal or life-threatening suspected adverse reaction as soon as possible, but in no case later than 7 calendar days after the sponsor's initial receipt of the information. Report using the FDA Form 3500a.

- The UVA CC is responsible for notifying the UVA IRB-HSR of any serious, unexpected adverse event within 7 calendar days from the time the study team receives knowledge of the event. Timeline includes submission of signed hardcopy of AE form. Report using IRB online.

- The UVA CC is responsible for notifying the FDA and all participating investigators in an Investigational New Drug (IND) safety report of potential serious risks, from clinical trials or any other source, as soon as possible, but in no case later than 15 calendar days after the sponsor determines that the information qualifies for reporting (21 CFR 312.32 (c)(1)). Other adverse event information should be sent to the FDA in the IND annual report. The FDA will be notified using an FDA Form 3500a.

8.6.3 Pregnant-Partner Outcomes

If a male has been exposed to the investigational agent prior to or around the time of conception, or within 3 months of conception, the pregnancy must be reported to the Sponsor within 2 working days of the study team learning of the event. This will not be considered an SAE. The HITC will ask permission of the pregnant partner to be followed until term.

Pregnancy

If a female has been exposed to the investigational agent while on treatment or within 3 months after stopping treatment, the pregnancy must be reported to the Sponsor within 2 working days of the study team learning of the event. The participant will be taken off of study treatment. The study team will follow the pregnancy until completion or termination. The site will contact the participant at least monthly and document the participant’s status.

The outcome of a pregnancy should be reported to the Sponsor within 2 working days of the study team learning of the event. Death, abortion, congenital anomaly, or other disability or life-threatening event to either the mother or newborn are considered serious adverse events, and should be reported as such in accordance with the guidelines for reporting serious adverse events.

8.6.4 IRB Reporting Requirements

The University of Virginia CC is responsible for reporting to the UVA IRB-HSR per the following guidelines (Table 18).

Table 18. UVA IRB-HSR reporting

Type of Event	To whom will it be reported:	Time Frame for Reporting	How reported?
Any internal event resulting in death that is deemed DEFINITELY related to (caused by) study participation (Note: An internal event is one that occurs in a subject enrolled in a Uva protocol.)	IRB-HSR	Within 24 hours	IRB Online and phone call<br><br>IRB Online
Internal, Serious, Unexpected adverse event.	IRB-HSR	Within 7 calendar days from the time the study team received knowledge of the event.<br><br>*Timeline includes submission of signed hardcopy of AE form.*	IRB Online<br><br>IRB Online
Unanticipated Problems that are not adverse events or protocol violations<br>This would include a Data Breach.	IRB-HSR	Within 7 calendar days from the time the study team received knowledge of the event.	Unanticipated Problem report form.<br><br>*http://www.virginia.edu/vprgs/irb/HSR_docs/Forms/Reporting_Requirements-Unanticipated_Problems.doc)*
Protocol Violations (*The IRB-HSR only requires that MAJOR violation be reported, unless otherwise required by your sponsor, if applicable.)*<br><br>Or<br><br>Enrollment Exceptions	IRB-HSR	Within 7 calendar days from the time the study team received knowledge of the event.	Protocol Violation and Enrollment Exception Reporting Form<br><br>http://www.virginia.edu/vpr/irb/HSR_docs/Forms/PROTOCOL_DEVIATION_PROTOCOL%20EXCEPTION_REPORTING_FORM.doc
Data Breach	The Uva Corporate Compliance and Privacy Office and<br><br>ITC: if breach involves electronic data-<br><br>Uva Police if breach includes such things as stolen computers.	As soon as possible and no later than 24 hours from the time the incident is identified.<br><br>As soon as possible and no later than 24 hours from the time the incident is identified.<br><br>IMMEDIATELY.	Uva Corporate Compliance and Privacy Office- Phone 924-9741<br><br>ITC: https://security.virginia.edu/report-information-security-incident<br><br>Phone- (434) 924-7166

8.6.5 Additional Reporting Requirements

Reporting to Celldex

- The following Events of Interest must be reported to the UVA CC who will report to Celldex, or designee, within 24 hours of becoming aware of the event:
  - o Any DLT, regardless of whether or not the event is considered an SAE
  - o Any ≥ Grade 2 irAE, as referenced the CDX-1127 Investigator's Brochure
  - o Any infusion reaction ≥ Grade 3
  - o Any ≥ Grade 2 drug-related unexpected toxicity
- Death due to disease progression will be reported to Celldex, if occurring within 28 days of study treatment
- All reports sent to the FDA will be also sent to Celldex Therapeutics.

8.6.6 Reporting of Participant Withdrawals/Dropouts Prior to Study Completion

Participants who withdraw consent and those dropping out of the study secondary to an AE will be reported to the UVA IRB yearly on the IRB continuation form.

8.7 Adverse Event Review and Monitoring

8.7.1 Capturing Adverse Events

In addition to clinic notes, adverse events will be initially captured using study-specific tools and participant toxicity diaries.

Each participant will be evaluated by a licensed clinician. The following will be performed as designated in the protocol: routine disease-directed physical exam including performance status and blood collection for clinical labs.

Participants may keep a daily diary of toxicities until the next protocol clinic visit. The toxicities will be reviewed by a research clinician prior to the next scheduled infusion or vaccine, if one is scheduled. During clinic visits, participants will also be asked about subjective symptoms including headache, malaise, fatigue, dyspnea, nausea, rash, diarrhea, abdominal discomfort, peripheral nerve pain, visual changes, appetite, tremors, night sweats, and ability to concentrate. Additional toxicities will be captured from laboratory tests. For each AE (with the exception of Grade 1 hematologic/metabolic events), date of onset, duration, grade, and attribution should be noted in the participant's study chart, on study documents, or in the clinic note, and will be entered into the UVA Cancer Center database. It will not be considered a protocol deviation if the participant fails to return the patient diary to the study team, provided that the participant's toxicities are reviewed at the study visit.

Follow-up phone calls will be made per the judgment of the research clinicians with regard to individual participant need. Participants will be instructed on how to reach their provider should they have any questions and/or problems during the study.

In the event of an AE, appropriate action will be taken to ensure adequate care for the participant. If the participant is still on protocol, treatment delay or withdrawal

from the protocol will be considered according to the protocol guidelines (Sections 5.5 and 5.6).

8.7.2 Review of Adverse Events by the Study Team

Individual AEs will be reviewed by the treating physician, principal investigator, and the clinical research coordinator(s) (CRC). Other staff on the research team may also review AEs.

SAEs will be reviewed about once per month by the PI and Sponsor during the UVA Melanoma Team Meeting. This meeting will occur at least 20 times in a calendar year. Those present at the meeting may include the sponsor/overall study PI, sub-investigators, protocol development staff, biostatisticians, research nurses, research coordinators, laboratory specialists, and laboratory research managers. These meetings also include the review of individual participants to assess whether they are protocol candidates, whether AEs warrant discontinuation, and whether existing protocols should be continued or closed.

8.8 Unanticipated Problems

Site Reporting Requirements

- UPs that are SAEs will be reported in accordance with the guidelines for SAE reporting.

- UPs that are not SAEs and DLTs should be reported to the UVA CC upon receiving knowledge of the event.

- UPs that are not adverse events or protocol deviations or data breaches
  - o Report to the UVA CC within 4 calendar days from the time the study team receives knowledge of the event.
  - o Report to your IRB of record in accordance with your IRB guidelines. (For sites that use the UVA IRB-HSR as the IRB of record, the UVA CC will report to the UVA IRB-HSR—see sponsor reporting requirements)

Sponsor Reporting Requirements
Note: The UVA CC may submit on behalf of the sponsor.

- The UVA CC will report UPs that are not adverse events or protocol deviations to the UVA IRB-HSR within 7 calendar days from the time the study team receives knowledge of the event. Report using the Unanticipated Problem Report form.

- All UPs will be reported to appropriate institutional officials (as required by an institution's written reporting procedures), the supporting agency head (or designee), and the Office for Human Research Protections (OHRP) in accordance with institutional policies.

If during the course of the study there is an unanticipated problem that affects current or past participants, affected participants will be contacted if needed.

8.9 Dose-limiting Toxicities

The study will be monitored continuously for treatment-related adverse events.

A DLT is defined as any adverse event that is possibly, probably or definitely related to investigational drug and meets the following criteria:

- The following ≥ Grade 2 ocular adverse events
  - Eye Disorders: Night blindness (nyctalopia)
  - Eye Disorders: Papilledema
  - Eye Disorders: Retinopathy

  Participants will be referred for an ophthalmologic exam if any of these ocular adverse events occur.

- A prolonged treatment-related experience (e.g., lasting > 5 days) of the following ≥ Grade 2 events will be classified as a DLT:
  - Eye Disorders: Vision Decreased. This would also include other eye disorders with a similar deficit in visual acuity.
  - Eye Disorders: Flashing lights
  - Eye Disorders: Floaters
  - Symptomatic pneumonitis with shortness of breath

  Participants will be referred for an ophthalmologic exam if any of these ocular adverse events occur.

- Allergic/autoimmune reactions ≥ Grade 2 will be classified as a DLT.

- Any Grade 3 or greater toxicity (including irAEs) with the exception of the following:
  1. Grade 3 inflammation due to local anti-tumor reactions ≤ 7 days in duration.
  2. Grade 3 injection site reaction with ulceration as long as the sum of maximum ulcer diameters does not exceed 2 cm in greatest dimension
  3. Grade 3 or 4 infusion reaction, where the treating investigator judges that the infusion reaction does not represent an allergic/anaphylactic reaction, and also judges that it is safe for the participant to restart infusion and to continue treatment on study.
  4. Grade 3 lymphopenia or Grade 4 lymphopenia that improves to ≤ Grade 3 or within 20% of baseline within 28 days from the last dose of CDX-1127.
  5. Grade 3 vaccination complications with necrosis, erythema ≤ 20 cm, and/or induration/swelling ≤ 20 cm. Vaccination complication with severe pain or limiting ADL is not included in this exception.
  6. Grade 3 neutropenia
  7. Grade 4 neutropenia < 7 days in duration
  8. Grade 3 nausea, vomiting, or diarrhea that resolves to ≤ Grade 1 with or without treatment within 48 hours

8.10 Data Breach

8.10.1 Definition of a Data Breach

A data breach is an unauthorized acquisition, access, or use of protected health information (PHI) that compromises the security or privacy of such information.

8.10.2 Reporting a Data Breach

Site Reporting Requirements

- Report to the UVA CC within 24 hours from the time the study team receives knowledge of the event.

- Report to your IRB of record in accordance with your IRB guidelines. (For sites that use the UVA IRB-HSR as the IRB of record, the UVA CC will report to the UVA IRB-HSR—see sponsor reporting requirements)

Sponsor Reporting Requirements
Note: The UVA CC may submit on behalf of the sponsor.

- Report to the UVA Corporate Compliance and Privacy Office as soon as possible and no later than 24 hours from the time the incident is identified. Report by telephone.

- Report to ITC if the breach involves electronic data. Report as soon as possible and no later than 24 hours from the time the incident is identified. Refer to the following for details: http://security.virginia.edu/report-information-security-incident.

- Report to UVA police if the breach includes such things as stolen computers. Report by telephone.

8.11 Protocol Deviation

8.11.1 Reporting a Protocol Deviation

It is the responsibility of the site investigator to use continuous vigilance to identify and report deviations. All deviations must be addressed in study source documents and reported in OnCore.

Site Reporting Requirements

- Report major deviations to the UVA CC within 4 calendar days from the time the study team receives knowledge of the event.

- Report to your IRB of record in accordance with your IRB guidelines. (For sites that use the UVA IRB-HSR as the IRB of record, the UVA CC will report to the UVA IRB-HSR as required—see sponsor reporting requirements)

Sponsor Reporting Requirements
Note: The UVA CC may submit on behalf of the sponsor.

- The UVA CC will report to the UVA IRB-HSR major deviations within 7 calendar days from the time the study team received knowledge of the event. Report using the Protocol Deviation and Protocol Exception Reporting Form.

- Minor deviations do not need to be reported to the UVA IRB-HSR.

8.12 Data Collection

8.12.1 Endpoint Data

- Endpoint data will be collected using HITC IML data forms, participant-specific binders, and the HITC laboratory database.
- The HITC laboratory database, which has password-restricted access, is stored on the UVA Health System Computing Services secured server.

8.13 Monitoring Plan

8.13.1 Safety Oversight

The University of Virginia Cancer Center Data and Safety Monitoring Committee (CC DSMC) will provide oversight of the conduct of this study. The CC DSMC will report to the UVA Protocol Review Committee (PRC).

The UVA CC DSMC will review the following:

- All adverse events
- Audit results
- Application of study designed stopping/decision rules
- Whether the study accrual pattern warrants continuation/action
- Protocol violations

The UVA CC DSMC will meet every month for aggregate review of data. Tracking reports of the meetings are available to the PI for review. Issues of immediate concern by the DSMC are brought to the attention of the sponsor (and if appropriate to the PRC and IRB) and a formal response from the sponsor is requested. Per the UVA Cancer Center NIH approved institutional plan, this study will be audited approximately every 6 months. The audit may include direct access to source data/documents.

Any study under the purview of the University of Virginia HSR-IRB is subject to review. Studies are chosen for Post-approval Monitoring either a) at random or b) requested by a study team member or any member of the IRB-HSR.

The purpose of Post-approval Monitoring audits is to ensure that documentation of clinical research studies is of the highest quality, verify protocol adherence, and ensure that all Federal and local rules concerning clinical research are being fulfilled. Post-approval monitoring is done by staff within the office of the Vice President for Research (VPR) in accordance with their Standard Operating Procedures. The conduct of an on-site review may include but is not limited to:

- Requests for progress reports from investigators
- Examinations of research records, including signed informed consent documents, protocol modifications, and unexpected, serious, and/or related adverse experience reports
- Contacts with research subjects
- Observation of the consent process and/or research procedures. Examples of when observation of the consent process could occur are:

- Full board IRB determines during review of a project that a conflict of interest exists such that the informed consent process should be observed by a neutral party;
- IRB is made aware of a complaint or concern with regard to the informed consent process; or
- IRB determines as a result of the monitoring process that the consent process is insufficient and education/training is required for conduct of consent.

8.13.2 Site Monitoring

Clinical site monitoring is conducted to ensure that the rights and well-being of trial participants are protected, that the reported trial data are accurate, complete, and verifiable, and that the conduct of the trial is in compliance with the currently approved protocol/amendment(s), with International Conference on Harmonisation Good Clinical Practice (ICH GCP), and with applicable regulatory requirement(s).

- The UVA CC will implement ongoing monitoring activities for this study to ensure that participating institutions are complying with regulatory and protocol requirements, data quality, and participant safety. Monitoring will occur before the clinical phase of the protocol begins, continue during protocol performance and through study completion.
- To ensure that a site will be ready to enroll patients, the UVA CC will review the necessary regulatory documents pertaining to that site. A site initiation visit will occur upon receipt of satisfactory regulatory documentation. An activation letter will be sent to participating sites to notify the study team that the site is open to enrollment.
- The site will notify the UVA CC when a subject has signed consent. The UVA CC will review source documents, including the eligibility packet, to confirm eligibility and enrollment.
- Monitoring may be conducted either remotely or on-site. For remote visits, each institution will be required to provide redacted source documents for review or appropriate access to the EMR. The UVA CC will provide the participating institution with a follow-up letter following completion of the monitoring visit which should be maintained in the site regulatory files. The schedule for monitoring may be adjusted according to participant accrual and data quality. The Investigator will be notified in advance of each visit.
- As applicable, queries will be sent to the site as prompted by database review and source document verification.

Independent audits may be conducted by each institution according to institutional guidelines. Results of these audits may be requested by the UVA CC.

8.13.3 Quality Assurance and Quality Control

Each clinical site will perform internal quality management of study conduct, data and biological specimen collection, documentation and completion according to institutional policies.

Quality control (QC) procedures will be implemented beginning with the data entry system and data QC checks that will be run on the database will be generated. Any missing data or data anomalies will be communicated to the site(s) for clarification/resolution.

Following written Standard Operating Procedures (SOPs), the monitors will verify that the clinical trial is conducted and data are generated and biological specimens are collected, documented (recorded), and reported in compliance with the protocol, International Conference on Harmonisation Good Clinical Practice (ICH GCP), and applicable regulatory requirements (e.g., Good Laboratory Practices (GLP), Good Manufacturing Practices (GMP)).

The investigational site will provide direct access to all trial related sites, source data/documents, and reports for the purpose of monitoring and auditing by the sponsor, and inspection by local and regulatory authorities.

8.14 Study Conduct and Ethical Considerations

This study will be conducted in accordance with the standards of Good Clinical Practice (GCP), all applicable federal, state, and local laws, and in accord with the ethical principles that originated in the Declaration of Helsinki. The PI will ensure that staff are trained and carry out the study in accord with the protocol specifications. The PI will ensure that all study site personnel are aware that the study protocol and all data generated are confidential and should not be disclosed to third parties (with the exception of local and national regulatory bodies which require access for oversight purposes).

8.14.1 UVA Institutional Review Board for Health Sciences Research

The UVA Institutional Review Board for Health Sciences Research (UVA IRB-HSR) will be the IRB of record for this study. The UVA IRB-HSR will approve all aspects of this study, including the clinical trial protocol, informed consent documents, and patient materials. Modifications to the protocol or consent form will be reviewed and approved by the UVA IRB-HSR prior to implementation, except when necessary to eliminate apparent immediate hazards to the study participants. The study will undergo continuing IRB review based on the level of risk as assessed by the IRB. This review will take place no less than annually. Reporting to the UVA IRB-HSR will occur as specified in Section 8.6.

8.14.2 Consent Forms and the Consenting Process

Consent forms will be written in accord with 21 CFR 50 and will be reviewed and approved by the UVA IRB-HSR prior to use. Participants will be given a consent form to review and a member of the study team will be available to answer any questions. Informed consent will be obtained from each participant prior to conducting any study-specific procedures or administering study drug.

8.14.3 Maintenance of Study Documents

Signed consent forms and other research records will be retained in a confidential manner. Record retention will be in accord with 21 CFR 312.62 and HIPAA regulations. Study documents should be retained for a minimum of 2 years after the last approval of a marketing application in an International Conference on

Harmonisation (ICH) region and until there are no pending or contemplated marketing applications in an ICH region or until at least 2 years have elapsed since the formal discontinuation of clinical development of the study intervention. These documents should be retained for a longer period, however, if required by local regulations. No records will be destroyed without the written consent of the sponsor. It is the responsibility of the sponsor to inform the investigator when these documents no longer need to be retained.

8.15 Study Discontinuation and Closure

This study may be temporarily suspended or prematurely terminated if there is sufficient reasonable cause. Written notification, documenting the reason for study suspension or termination, will be provided by the suspending or terminating party to the UVA CC. If the study is prematurely terminated or suspended, the Principal Investigator (PI) will promptly inform the Institutional Review Board (IRB) and Sponsor and will provide the reason(s) for the termination or suspension. Study participants will be contacted, as applicable, and be informed of changes to study visit schedule.

Circumstances that would warrant termination or suspension include, but are not limited to:

- Determination of unexpected, significant, or unacceptable risk to participants
- Demonstration of efficacy that would warrant stopping
- Insufficient compliance to protocol requirements
- Data that are not sufficiently complete and/or evaluable
- Determination that the primary endpoint has been met
- Determination of futility
- Change in funding status

Study may resume once concerns about safety, protocol compliance, and data quality are addressed, and satisfy the sponsor, IRB, Data and Safety Monitoring Committee and/or Food and Drug Administration (FDA).

Participants receiving study treatment at the time of study discontinuation should complete procedures described in Section 5.6 and in Section 9.1.

9.0 APPENDICES

9.1 Study Calendars

Arm A: 6MHP + Montanide ISA-51 +polyICLC + CDX-1127

Studies & Tests	Pre	Active Treatment										
	Day	1	8	15	22	36	57	78	85	127	176	183[n]
	Week	0	1	2	3	5	8	11	12	18	25	26
Informed consent	X[a]											
Pathology review	X[a]											
CBC with differential	X[b,]	X[f]		X	X	X	X	X	X	X	X	X
Comprehensive chemistry	X[b,e]	X[f]		X	X	X	X	X	X	X	X	X
HGBA1C	X[b]											
Urinalysis	X[b]				X		X					
β-HCG [g]	X[c]	X				X		X				
HIV / Hepatitis C	X[d]											
CT chest/abdomen/pelvis or PET-CT	X[b]											
CXR or other imaging, if indicated.									X[m]			X[m]
Head MRI / CT	X[b]											
Vital signs [j]	X[b]	X[k]	X	X	X	X[k]	X	X[k]	X	X	X	X
History & physical [i]	X[b]	X[f]	X	X	X	X	X	X	X	X	X	X
Medication review	X[b]	X	X	X	X	X	X	X	X	X	X	X
Toxicity assessment (or baseline)		X	X	X	X	X	X	X	X	X	X	X
Designation of potential vaccination sites	X[b]											
Assessment of skin and nodal basins for evidence of disease	X[b]											
Assessment of skin for vitiligo		X			X				X		X	
Assessment of hair and eye color		X			X				X		X	
Research bloods		X[h]		X	X	X	X	X	X	X	X	X
Anti-nuclear antibody / Rf		X					X					
Vaccination with 6MHP + Montanide ISA-51 + polyICLC		X	X	X		X	X[o]	X[o]				
Vaccination with 6MHP + Montanide ISA-51											X	

Studies & Tests	Pre	Active Treatment										
	Day	1	8	15	22	36	57	78	85	127	176	183[n]
	Week	0	1	2	3	5	8	11	12	18	25	26
CDX-1127		X				X		X				
Skin biopsy at vaccine site					X				X[l]			
Participant diary reviewed and/or distributed		X	X	X	X	X	X	X	X	X	X	X

[a] Any point prior to registration
[b] Pre-study within 6 weeks of registration
[c] Within 2 weeks of registration (for childbearing women)
[d] Within 6 months of registration
[e] Not required to include fasting glucose
[f] History & physical, comprehensive chemistry, and CBC with differential scheduled for Day 1 are not required if pre-study assessments were within 10 calendar days of day 1.
[g] Pregnancy tests are required only for women of childbearing potential (excluding patients who are post-menopausal with absence of menses for at least 1 year and/or surgically sterilized). A serum pregnancy or urine pregnancy test is required within 72 hours prior to dosing on Day 1. The Day 1 pregnancy test does not need to be repeated if the screening pregnancy test was done within the 72 hours prior to dosing.
[h] Blood for HLA typing is included in the research bloods.
[i] Includes height (at screening only), weight, performance status, and neurologic function-general
[j] Includes temperature, pulse, respiratory rate and blood pressure
[k] Vitals should be assessed pre-infusion and 1 hour post-infusion
[l] The vaccine site biopsy at day 85 was initially required for all participants, except that it was optional for participants who had discontinued vaccine therapy before vaccine 6 but had not discontinued varlilumab therapy, and it should not have been done if the participant had ulceration at the vaccine site or had a vaccine-site related DLT. To reduce vaccine site ulceration, this biopsy at day 85 (week 12) was removed and should no longer be done after approval of Protocol v12-03-2020.
[m] Tumor imaging does not need to be repeated if scans have been completed within the past 8 weeks.
[n] If the day 176 visit is delayed, the day 183 visit needs to be 7 days later than the day 176 visit. The range of days in the delayed visit table (Table 7) applies around the 7 day interval.
[o] Vaccines 5 and 6 (days 57 and 78) will not be administered after approval of Protocol v12-03-2020.

Arm B: 6MHP + Montanide ISA-51 +polyICLC

Studies & Tests	Pre	Active Treatment										
	Day	1	8	15	22	36	57	78	85	127	176	183[m]
	Week	0	1	2	3	5	8	11	12	18	25	26
Informed consent	X[a]											
Pathology review	X[a]											
CBC with differential	X[b,]	X[f]		X	X	X	X	X	X	X	X	X
Comprehensive chemistry	X[b,e]	X[f]		X	X	X	X	X	X	X	X	X
HGBA1C	X[b]											
Urinalysis	X[b]				X		X					
β-HCG [g]	X[c]											
HIV / Hepatitis C	X[d]											
CT chest/abdomen/pelvis or PET-CT	X[b]											
CXR or other imaging, if indicated									X[l]			X[l]
Head MRI / CT	X[b]											
Vital signs [j]	X[b]	X	X	X	X	X	X	X	X	X	X	X
History & physical [i]	X[b]	X[f]	X	X	X	X	X	X	X	X	X	X
Medication review	X[b]	X	X	X	X	X	X	X	X	X	X	X
Toxicity assessment (or baseline)		X	X	X	X	X	X	X	X	X	X	X
Designation of potential vaccination sites	X[b]											
Assessment of skin and nodal basins for evidence of disease	X[b]											
Assessment of skin for vitiligo		X			X				X		X	
Assessment of hair and eye color		X			X				X		X	
Research bloods		X[h]		X	X	X	X	X	X	X	X	X
Anti-nuclear antibody / Rf		X					X					
Vaccination with 6MHP + Montanide ISA-51 + polyICLC		X	X	X		X	X[n]	X[n]				
Vaccination with 6MHP + Montanide ISA-51											X	
Skin biopsy at vaccine site					X				X[k]			
Participant diary reviewed and/or distributed		X	X	X	X	X	X	X	X	X	X	X

[a] Any point prior to registration
[b] Pre-study within 6 weeks of registration

[c] Within 2 weeks of registration (for childbearing women)
[d] Within 6 months of registration
[e] Not required to include fasting glucose
[f] History & physical, comprehensive chemistry, and CBC with differential scheduled for Day 1 are not required if pre-study assessments were within 10 calendar days of day 1.
[g] Pregnancy tests are required only for women of childbearing potential (excluding patients who are post-menopausal with absence of menses for at least 1 year and/or surgically sterilized). A serum pregnancy or urine pregnancy test is required within 72 hours prior to dosing on Day 1. The Day 1 pregnancy test does not need to be repeated if the screening pregnancy test was done within the 72 hours prior to dosing.
[h] Blood for HLA typing is included in the research bloods.
[i] Includes height (at screening only), weight, performance status, and neurologic function-general
[j] Includes temperature, pulse, respiratory rate and blood pressure
[k] The vaccine site biopsy at day 85 was required for all participants, except that it was optional for participants who have discontinued vaccine therapy before vaccine 6, and it should not have been done if the participant had ulceration at the vaccine site or had a vaccine-site related DLT. To reduce vaccine site ulceration, this biopsy at day 85 (week 12) was removed and should no longer be done after approval of Protocol v12-03-2020.
[l] Tumor imaging results should be collected if scans have been completed within the past 8 weeks.
[m] If the day 176 visit is delayed, the day 183 visit needs to be 7 days later than the day 176 visit. The range of days in the delayed visit table (Table 7) applies around the 7 day interval.
[n] Vaccines 5 and 6 (days 57 and 78) will not be administered after approval of Protocol v12-03-2020.

Studies & Tests	End of Treatment (EOT)		Follow-Up	
	EOT 1[c]	EOT 2	Follow-up Discontinuation of study drugs[a] (FUV1)	Survival Follow-up
	Premature discontinuation of one study drug and/ or all study drugs	7 days post premature discontinuation of vaccine at EOT 1	30 days post discontinuation	Annually
CBC with differential	X	X		
Comprehensive chemistry	X	X		
Urinalysis	X			
Imaging as clinically indicated	X	X		
Physical Exam	X	X		
Medication review	X	X		
Toxicity assessment	X	X	X	
Assessment of skin and nodal basins for evidence of disease	X			
Assessment of skin for vitiligo	X			
Assessment of hair and eye color	X			
Research bloods	X	X[d]		
Anti-nuclear antibody / Rf	X			
Participant diary reviewed and/or distributed	X	X	X	
Survival				
Survival Status				X
Start of Antineoplastic therapy				X

[a] Patients are to be contacted 30 days after the last administration of study drug to obtain adverse event data. This may be done by phone, mail, email, or in person.
[b] Subjects may be contacted by telephone for survival follow-up status. The expectation is that there will be additional follow-up as part of standard of care, either by the study physician or by a local referring physician. Every effort will be made to obtain data on disease status and survival at all such visits, in addition to the mandated study visits.
[c] If a subject is discontinued and the assessments have been completed as part of a regularly scheduled visit within 7 days, the assessments do not need to be repeated.
[d] If a subject has had research bloods drawn ≥6 days after the last administered vaccine, the research bloods do not need to be drawn again.

9.2 AJCC Staging System (AJCC version 8)

Cutaneous Melanoma TNM Classification

T Classification	Thickness	Ulceration Status, thickness detail
TX	Thickness cannot be assessed	
T0	No evidence of primary tumor (eg unknown or regressed primary)	
Tis	Melanoma in situ	
T1	≤ 1.0 mm	a: < 0.8 mm* without ulceration
		b: ulcerated or 0.8* – 1.0 mm
T2	1.1 – 2.0 mm	a: without ulceration
		b: with ulceration
T3	2.1 – 4.0 mm	a: without ulceration
		b: with ulceration
T4	> 4.0 mm	a: without ulceration
		b: with ulceration

N Classification		# / size of Metastatic Nodes	Intransit/satellite metastases
NX		Regional nodes not assessed: Except for T1, use cN.	
N0		None	none
N1	a	1 clinically occult node	none
	b	1 clinically detected node	none
	c	None	Yes
N2	a	2-3 clinically occult nodes	none
	b	2-3 nodes, with at least 1 clinically detected	none
	c	1 node (clinically occult or clinically detected)	Yes
N3	a	4 or more clinically occult nodes	none
	b	4 nodes, with at least 1 clinically detected; or any matted nodes	none
	c	2 or more nodes (clinically occult or clinically detected) and/or matted nodes	Yes

M Classification	Site	Serum Lactate Dehydrogenase
M0	No evidence of distant metastasis	
M1a	Distant skin, SQ, soft tissue (incl muscle), or nodal mets	(0) Not elevated
		(1) Elevated
M1b	Lung metastases	(0) Not elevated
		(1) Elevated
M1c	Other non-CNS visceral metastases	(0) Not elevated
		(1) Elevated
M1d	CNS metastasis	(0) Not elevated
		(1) Elevated

* Breslow thickness to be rounded to first decimal place: 0.75 – 0.79 is rounded to 0.8

Stage Groupings for Cutaneous Melanoma

	Clinical Staging*			Pathologic Staging†		
	**T**	**N**	**M**	**T**	**N**	**M**
**0**	Tis	N0	M0	Tis	N0	M0
**IA**	T1a	N0	M0	T1a	N0	M0
**IB**	T1b<br>T2a	N0<br>N0	M0<br>M0	T1b<br>T2a	N0<br>N0	M0<br>M0
**IIA**	T2b<br>T3a	N0<br>N0	M0<br>M0	T2b<br>T3a	N0<br>N0	M0<br>M0
**IIB**	T3b<br>T4a	N0<br>N0	M0<br>M0	T3b<br>T4a	N0<br>N0	M0<br>M0
**IIC**	T4b	N0	M0	T4b	N0	M0
**III‡**	Any T	N1-3	M0			
**IIIA**				T1a/1b/2a	N1a/2a	M0
**IIIB**				T0<br>T1a/1b/2a<br>T2b/3a	N1b/1c<br>N1b/1c/2b<br>N1a/1b/2a/2b	M0<br>M0<br>M0
**IIIC**				T0<br>T1a/1b/2a/2b/3a<br>T3b/4a<br>T4b	N2b/2c/3b/3c<br>N2c/N3<br>N1-3<br>N1-2	M0<br>M0<br>M0<br>M0
**IIID**				T4b	N3	M0
**IV**	Any T	Any N	Any M1	Any T	Any N	M1

* Clinical staging includes microstaging of the primary melanoma and clinical/radiologic evaluation for metastases. By convention, it should be used after complete excision of the primary melanoma with clinical assessment for regional and distant metastases.

† Pathologic staging includes microstaging of the primary melanoma and pathologic information about the regional lymph nodes after partial or complete lymphadenectomy. Pathology stage 0 or stage 1A patients are the exception; they do not require pathologic evaluation of their lymph nodes.

‡ There are no stage III subgroups for clinical staging.

Staging for mucosal melanomas, for the purpose of this study, will be based on AJCC staging for head and neck melanomas, with some modifications for other mucosal sites as detailed below:

Staging for Mucosal Melanomas of the Head and Neck (AJCC version 8, chapter 14)

Mucosal Melanoma of the Head and Neck: TNM Classification

T Classification	Thickness
T3	Limited to the mucosa and immediately underlying soft tissue, regardless of thickness or dimension.
T4a	Moderately advanced disease, involving deep soft tissue, cartilage, bone, or overlying skin
T4b	Very advanced disease, involving brain, dura, skull base, lower cranial nerves (IX-XII), masticator space, carotid artery, prevertebral space, or mediastinal structures.

N Classification	# / size of Metastatic Nodes
NX	Regional nodes not assessed: Except for T1, use cN.
N0	None
N1	Regional node metastases present

M Classification	Metastatic status
M0	No evidence of distant metastasis
M1	Distant metastasis present

In AJCC version 8, no stage groupings were provided; so we will use those listed in AJCC version 7:

Anatomic stage/prognostic groups for mucosal melanoma of the Head and Neck, from AJCC v7.

Stage	T	N	M
III	T3	N0	M0
IVA	T4a	N0	M0
	T3-4a	N1	M0
IVB	T4b	Any N	M0
IVC	Any T	Any N	M1

However, for mucosal melanomas of the female genital tract and anorectum, some patients present with thin melanomas that appear to carry a good prognosis; so we favor not classifying them as stage III. Thus, we will adopt this modified staging system for all mucosal melanomas but will stage similarly to cutaneous melanomas. Otherwise, the staging for these melanomas will mimic that of head and neck mucosal melanomas:

**Staging for Mucosal Melanomas of the Anorectum or Female Genital Tract**	
**T Classification Anorectal and Female Genital**	**Thickness/Depth of invasion**
T1a	< 1 mm depth, without ulceration
T1b	< 1 mm depth, with ulceration present or not evaluable
T2a	1 -2 mm depth, without ulceration
T2b	1 - 2 mm depth, with ulceration present or not evaluable

T3	> 2 mm deep or with unmeasurable depth, and limited to the mucosa or superficial soft tissue but not invading muscle but muscular wall of anorectum or female genital tract.
T4a	Moderately advanced disease, involving deep soft tissue, such as the muscular wall of the anorectum or female genital tract
T4b	Very advanced disease, invading through the wall of the anorectum or vagina, and/or into the peritoneal cavity, or into bone of the pelvis.

**N Classification**	**# / size of Metastatic Nodes**
NX	Regional nodes not assessed: Except for T1, use cN.
N0	None
N1	Regional node metastases present

**M Classification**	**Metastatic status**
M0	No evidence of distant metastasis
M1	Distant metastasis present

Anatomic stage/prognostic groups for mucosal melanoma of the anorectal and female genital tract, modified from those for mucosal melanomas of the head and neck, and from those for cutaneous melanomas:

**Stage**	**T**	**N**	**M**
IA	T1a	N0	M0
IB	T2a/1b	N0	M0
II	T2b	N0	M0
III	T3	N0	M0
IVA	T4a	N0	M0
	T3-4a	N1	M0
IVB	T4b	Any N	M0
IVC	Any T	Any N	M1

9.3 ECOG Performance Status

ECOG PERFORMANCE STATUS*	
**Grade**	**ECOG**
0	Fully active, able to carry on all pre-disease performance without restriction
1	Restricted in physically strenuous activity but ambulatory and able to carry out work of a light or sedentary nature, e.g., light house work, office work
2	Ambulatory and capable of all selfcare but unable to carry out any work activities. Up and about more than 50% of waking hours
3	Capable of only limited selfcare, confined to bed or chair more than 50% of waking hours
4	Completely disabled. Cannot carry on any selfcare. Totally confined to bed or chair
5	Dead

* As published in Am. J. Clin. Oncol.:
Oken, M.M., Creech, R.H., Tormey, D.C., Horton, J., Davis, T.E., McFadden, E.T., Carbone, P.P.: Toxicity And Response Criteria Of The Eastern Cooperative Oncology Group. Am J Clin Oncol 5:649-655, 1982.

9.4 New York Heart Association Disease Classification

Functional Capacity	Objective Assessment
**Class I.** Patients with cardiac disease but without resulting limitation of physical activity. Ordinary physical activity does not cause undue fatigue, palpitation, dyspnea, or anginal pain.	No objective evidence of cardiovascular disease.
**Class II.** Patients with cardiac disease resulting in slight limitation of physical activity. They are comfortable at rest. Ordinary physical activity results in fatigue, palpitation, dyspnea, or anginal pain.	Objective evidence of minimal cardiovascular disease
**Class III.** Patients with cardiac disease resulting in marked limitation of physical activity. They are comfortable at rest. Less than ordinary activity causes fatigue, palpitation, dyspnea, or anginal pain.	Objective evidence of moderately severe cardiovascular disease.
**Class IV.** Patients with cardiac disease resulting in inability to carry on any physical activity without discomfort. Symptoms of heart failure or the anginal syndrome may be present even at rest. If any physical activity is undertaken, discomfort is increased.	Objective evidence of severe cardiovascular disease.

* The Criteria Committee of the New York Heart Association. Nomenclature and Criteria for Diagnosis of Diseases of the Heart and Great Vessels. 9th ed. Boston, Mass: Little, Brown & Co; 1994:253-256

9.5 RECIST 1.1 Criteria

Please refer to the following publication for evaluation of clinical response by RECIST 1.1.

E.A. Eisenhauer et al. New response evaluation criteria in solid tumours: Revised RECIST guideline (version 1.1). European Journal of Cancer, 2009, 45: 228-247. PMID: 19097774

9.6 Summary of Changes

Version Date	Description of changes
02-05-24	1. Arm assignments in Table 9 within Section 7 were corrected to be consistent with the arm assignments described throughout the protocol (Arm A: 6MHP + CDX-1127; Arm B: 6MHP only). Text in Section 7.0 was revised to clarify which group received which treatment for consistency.
09-28-22	2. Updated contact information for the Data Coordinating Center<br>3. Revised sections 4.2.3 and 4.4, as poly-ICLC will now be provided by the Sponsor (rather than Celldex Therapeutics, Inc.)
12-03-20	1. Revised the table of contents and revised formatting throughout the document.<br>2. In the summary section and in Sections 5, 6, and 7, revised the protocol to state that biopsies will no longer be done at week 12 (day 85) for participants whose week 12 visit would occur after approval of the 12/03/2020 protocol version.<br>3. In the summary section and in Section 5, revised the protocol to reduce the number of vaccines from 7 to 5 (vaccines now to be administered on days 1, 8, 16, 36, and a booster at day 176).<br>4. In the summary section and in Section 5, revised the protocol to state that the vaccine administration route is to be 100% subcutaneous (rather than half subcutaneous and half intradermal).<br>5. In Section 1.1, described changes listed above and summarized vaccine site ulceration events.<br>6. Added Section 1.7 to describe human experience with the trial regimen on the Mel 65 trial in the first 15 participants.<br>7. In Section 7.4, updated the DLT stopping bounds to state that stopping guidelines will be applied separately to participants accrued before and after this amendment.<br>8. In Section 9.1, updated the study calendars to reflect the above changes (removed vaccines at days 57 and 85 and skin biopsy at vaccine site at day 85).
07-13-20	1. Revised the table of contents and revised formatting throughout the document.<br>2. In Section 3.2, added the following to the exclusion criteria: Participants with prior autoimmune pneumonitis.
04-22-20	1. Revised the table of contents and revised formatting throughout the document.<br>2. In Section 5.5.1 and 5.5.2, corrected timeframes for study visits.<br>3. In Section 5.5.2, revised Table 7 to reflect the calendar revisions.<br>4. In Section 9.1, clarified the post-vaccine visits by adding day 183 to the active treatment calendar and by re-naming the FUv1 visit to EOT 2, which will not be done if a subject completes active treatment (day 183). This revision does not add or revise any study procedures, only re-names and rearranges them for clarity. Footnotes formerly “d” and “e” were removed from the discontinuation/follow-up calendar to avoid redundancy. Footnotes were added to the active treatment calendars to provide scan collection timeframes and to provide a clear timeframe for the new day 183 visit. A footnote was added to the

	discontinuation/follow-up calendar to indicate that research bloods are not required at EOT 2 under certain circumstances.
02-03-20	1. Revised the table of contents and revised formatting throughout the document.<br>2. In Section 4.3.2, revised CDX1127 storage and handling instructions to reflect updated CDX-1127 IB v01-25-2020.<br>3. In Section 5.3.5, added a ±10 minute infusion time window for CDX-1127.<br>4. In Section 8.6.2, moved UVA CC SAE reporting requirements to the "Sponsor Reporting Requirements" section to clarify that this responsibility belongs to the Sponsor or to the UVA CC on behalf of the sponsor.
10-29-19	1. Revised the table of contents and revised formatting throughout the document.<br>2. Updated the personnel section.<br>3. In the protocol synopsis section and caption of Figure 1, revised the indication to include that high-risk melanoma could also be determined by the DecisionDX-UM test.<br>4. In the protocol synopsis section and in the corresponding Section 2.2, removed a duplicate endpoint and corrected secondary endpoint #1.<br>5. In Section 3.1, clarified the inclusion criteria for participants with uveal melanoma.<br>6. In Section 5.6, clarified the criteria for discontinuation of therapy.<br>7. In Section 5.9, clarified that administration of non-live vaccines is acceptable 2 weeks before or after study drug is given.<br>8. In Section 5.13, added language from a study calendar footnote to the biopsy section to ensure that the Day 85 vaccine site biopsy requirements are clear throughout the protocol.<br>9. In Section 7.1.2 and Section 7.5, corrected that immunogenicity in tumor microenvironment will be done at weeks 3 and 12 per the study endpoints.<br>10. In Section 8.1.1, clarified the definition of an Adverse Event (AE).<br>11. In section 8.7.1, clarified that if a participant fails to return the patient diary it will not be considered a protocol deviation if AEs are reviewed at the study visit.<br>12. In Section 9.1, clarified footnote l for Arm A and footnote k for Arm B regarding the day 85 vaccine site biopsy.<br>13. In Section 9.2, clarified the staging system for mucosal melanomas.
07-24-19	1. Revised the Table of Contents<br>2. Revised formatting throughout the document<br>3. In sections 5.10 and 8.6.5, updated reporting requirements to specify that grade 3 or greater infusion reactions should be reported to Celldex (instead of grade 2 or greater) per CDX-1127 Investigator's brochure v7.0.<br>4. In sections 5.10, 8.6.1, 8.6.2, and 8.6.5, revised the language to state that event reporting to Celldex should occur by the UVA CC, and the timeframe for reporting begins when the UVA CC becomes aware of the event.<br>5. In section 9, removed the fasting glucose requirement in the footnotes of the study calendars.<br>6. Edited the summary of changes in section 9.6 for clarity

07-02-19	1. Removed oral cyclophosphamide as study therapy. Removed references to oral cyclophosphamide as applicable throughout the document, including in the schema, protocol calendars, objectives, and statistics sections.<br>2. Added a multi-site component and references to this component throughout the document. This includes the addition of sections detailing monitoring and reporting requirements, revision of the personnel involved in this study, and revisions to study drug supply language.<br>3. Information from the Mel 63 trial was updated throughout the document due to new results.<br>4. Revised the Table of Contents.<br>5. Revised formatting throughout the document.<br>6. An investigator signature box was added to the beginning of the document.<br>7. The List of Abbreviations (Table 2) was updated to include new response definitions, to include the UVA CC, and to remove Cy (cyclophosphamide).<br>8. The protocol schema was clarified to include that patients with stage IIA melanoma at diagnosis may be eligible for the trial and to remove cyclophosphamide.<br>9. Removed the day -6 visit and moved the procedures done on that visit to day 1, if not present on that day previously. Revisions to reflect this change were incorporated throughout the protocol, including in the protocol schema and study calendars.<br>10. Clarified that CTCAE version 5.0 should be used for AE assessment throughout the document.<br>11. Added secondary objective (2e) and associated endpoint.<br>12. Updated section 1.3 to reflect new data regarding ocular toxicities and to update previous study results.<br>13. Clarified that varlilumab will be administered every 5-6 weeks in section 1.6.2.<br>14. Clarified section 1.6.4 to note that current data regarding previous human experience for varlilumab can be found in the most current IB.<br>15. Revised order of inclusion criteria in section 3.1.<br>16. In section 3.3, clarified that the study does not involve blinding or masking procedures.<br>17. Revised section 4 for clarity and to provide additional drug supply and accountability information.<br>18. Revised section 5.2.3 for clarity.<br>19. Revised table 7 and section 6.6 to reflect protocol calendar revisions.<br>20. In section 7, revised statistical language and response language. Revised endpoint language.<br>21. Added monitoring, reporting, and study discontinuation and closure information to section 8.<br>22. In section 8.1, revised DLT criteria. Certain grade 1 reportable criteria were changed to reportable only at grade 2 or higher. Changed protocol “violation” to “deviation” for consistency with other protocols.<br>23. Added additional reporting requirement language to section 8 to add multi-site language and for consistency with other protocols.

	24. Revised section 9.1 for clarity and to incorporate changes listed above. Removed days -6 and day 50 to reflect changes listed above. Added a footnote to clarify biopsy requirements. Removed the biopsy at the vaccine site at the End of Treatment visit.
10-25-18	1. Revised the Table of Contents.<br>2. Revised formatting throughout the document.<br>3. In section 6.4, revised the language to clarify that results must be reviewed before each dose of varlilumab.
09-27-18	1. Revised the Table of Contents.<br>2. Revised formatting throughout the document.<br>3. Updated the personnel and contact information in the "Clinical Research Coordinators, Data Management, Regulatory Staff at the University of Virginia" section.<br>4. In section 3.1, added that patients with uveal melanoma will not require a pathology review for inclusion to the study.<br>5. In sections 3.1 and 3.2, changed the acceptable HgA1c level to ≤8.5%.<br>6. In section 5.2.1, added that on days when both varlilumab and vaccine are administered, the vaccine may be administered before or after varlilumab, but preferably before.<br>7. In section 5.4.3, included premedication instructions for subjects who experienced a grade 2 infusion related reaction during a past infusion.<br>8. In sections 5.9 and 5.10, clarified the acceptable use of steroids during study treatment.<br>9. In section 5.11, revised to remove specific dosing language for Grade 3 or Grade 4 reactions, and replace with language to indicate that treatment is dependent upon investigator judgment. An additional statement was added to refer to institutional guidelines for the treatment of anaphylaxis<br>10. Removed the visual acuity and color vision testing in sections 6.1 and 9.1. Participants may be referred to ophthalmology for a thorough eye examination if they develop clinical symptoms.<br>11. In section 6.4, indicated that a direct bilirubin test should occur regardless of total bilirubin results.<br>12. In section 8.6.4, added reference to the IB for a description of irAEs..<br>13. Changed "diaries" review to "toxicities" review in section 8.7.1.<br>14. In section 8.8, clarified the DLT criteria for a grade 3 injection site reaction and added grade 2 symptomatic pneumonitis with shortness of breath as a DLT when ≥ grade 2. Additionally, added circumstances for which a grade 3 or 4 infusion related reaction will not be a DLT.<br>15. In section 9.1, corrected footnote "g" to read that pregnancy testing is required within 72 hours prior to dosing on day -6.<br>16. In section 9.6, corrected to include changes made to the 7/20/17 version of this protocol.
06-14-18	1. Revised formatting and links throughout the document.<br>2. Section 1.3.3: Removed reference to a table summarizing previous trial data.<br>3. Removed Research Bloods collection for screening visit in section 9.1 (Study Calendars).
05-31-18	1. Revised Table of Contents.<br>2. Revised formatting and links throughout study document.

	3. Revised the protocol schema image to remove reference to CDX-1401.<br>4. Revised CDX1127 to CDX-1127 throughout the study document.<br>5. Precis and Section 2.1: revised wording for the objectives because CDX-1401 was removed from the protocol.<br>6. Removed references to CDX 1401 and NY-ESO 1 from Protocol synopsis and sections 1.1, 1.2, 1.5, 1.8, 2.1, 2.2, 2.3, 3.2, 5.5, 5.6, 5.13, 7.0, 7.1.1, 7.1.2, 8.4.3, and 9.1. Removed former sections 1.4 [Study Drug: CDX-1401 (3G9-NY-ESO-1)], 4.3 [CDX-1401 Vaccine (3G9-NY-ESO-1)], 5.3 (Administration of CDX-1401 with PolyICLC), and 5.6.2 (CDX-1401 and PolyICLC).<br>7. Section 1.3.1, 1.3.3, 5.2.1: removed reference to cohort 2.<br>8. Precis and section 7.2: revised accrual goal from 38 to 34<br>9. Section 3.3 and 7.3: revised to remove allocation and to include additional instructions for randomization.<br>10. Section 4.1.8: removed reference to the vaccine manual and instead refer to vaccine mixing instructions.<br>11. Removed references to Cohort 1 from Protocol synopsis and sections 7.0, 7.2, 7.3, 7.5, and 9.1. Removed section header 7.5.1 (Cohort 2) and section 7.5.2 (Cohort 1).<br>12. Removed NY-ESO-1 and HaHa testing references from sections 6.6, 7.1.2, and 9.1. Removed sections 6.3 (Archival Tissue NY-ESO-1 Expression) and 6.4 (HaHa Testing).<br>13. Changed “Cohort 2A” to “Arm A” and “Cohort 2B” to “Arm B” in sections 5.4, 5.4.1, 7.0, and 9.1.<br>14. Section 4.1.6, 4.1.8, 5.2.1: removed reference to a vaccine manual; stability testing will be completed yearly and directions for mixing the vaccine will be provided on the vaccine mixing sheets.<br>15. In Section 8.4.3 (Agent-Specific Events List): changed expected toxicities for PolyICLC and 6MHP to include grade 2 and grade 3 vaccination complication. Changed footnote “a” to that vaccine related toxicities will be captured under vaccine complications. Added footnotes b and c to provide a description of what skin toxicities are expected.<br>16. Section 8.5: revised to include all eye disorders under the ocular classification.<br>17. In Section 8.8 (Dose-limiting Toxicities) the DLT definition was revised to include the following exceptions: a) grade 3 skin ulceration at a vaccine site with no ulcer exceeding 2 cm in greatest dimension, and b) Grade 3 vaccination complications with necrosis, erythema ≤ 20 cm, and/or induration/swelling ≤ 20 cm. Vaccination complication with severe pain or limiting ADL is not included in this exception.

	Removed reference to exception for injection site reaction as vaccine related toxicities will be captured under the category of vaccination complication per CTCAE v5.<br><br>DLT rules for ocular toxicities were revised to increase prolonged treatment-related ocular experiences from ≥ Grade 1 to ≥ Grade 2. Eye disorders: blurred vision was replaced with Eye Disorders: Vision Decreased (a new category for CTCAE v5), which includes other eye disorders with a similar deficit in visual acuity.<br><br>18. In Section 9.1 (Study Calendars) removed footnote 'k' in the Arm A calendar next to "Vital Signs" on days 15 and 57 to reflect that pre- and post-infusion vital collection is no longer applicable.
04-16-18	4. Revised Table of Contents<br>5. Revised formatting and links throughout study document.<br>6. Section 1.3: data summary for Mel63 has been revised.
03/26/18	1. Revised Table of Contents<br>2. Revised formatting throughout the document<br>3. Precis:<br>• Revised version number of CTCAE from 4.03 to 5<br>• Revised protocol schema to change dose of polyICLC from 1 mg to 0.9 mg. Reduced the number of doses of CDX-1127 from 5 to 3 (Day -6, Day 36, Day 78)<br>4. Section 1.2: Revised to update the section describing the use of nivolumab in the adjuvant setting.<br>5. Section 1.6.3: Revised to clarify prior human experience with polyICLC.<br>6. Section 1.6.4: Added to describe the dose of polyICLC.<br>7. Section 1.7.2: Added to describe the raltionale for the dose and regimen of CDX-1127.<br>8. Section 2.1: Updated the version number for the CTCAE from 4.03 to 5.<br>9. Section 3.2 (13): Revised to replace Sjogren's syndrome with Addison's disease.<br>10. Section 4.4.1: Revised dose label for polyICLC from 2.0 mg/mL to 1.8 mg/mL solution (1.8 mg/mL "dry" polyIC concentration).<br>11. Section 5.2.2: Revised dose of polyICLC from 1 mg to 0.9 mg.<br>12. Section 5.3.2: Revised dose of polyICLC from 1 mg to 0.9 mg.<br>13. Section 5.5.1: Revised number of doses of CDX-1127 from 5 to 3 (Day<br>-6, Day 36, Day 78).<br>14. Tables 5 and 6: removed CDX-1127 (Varlilumab) from Day 15 and Day 57.<br>15. Section 5.12: Revised version of CTCAE from 4.03 to 5.<br>16. Section 6.3: Revised to clarify that serotype sampling for NY-ESO-1 expression will be completed on serum samples collected at screening.

	17. Section 6.4: Added a section describing HaHa testing.<br>18. Table 7: Removed HaHa testing as a separate lab and clarified which labs/tests would be completed using the bloods for correlative studies<br>19. Section 6.7: Revised version of CTCAE from 4.03 to 5.<br>20. Section 6.8: Revised to clarify the use of the research bloods. Added a table to specify blood amounts at each visit.<br>21. Section 7.4: Revised version of CTCAE from 4.03 to 5.<br>22. Section 8.4.1: Revised version of CTCAE from 4.03 to 5.<br>23. Section 8.4.3:<br>• Revised dose of polyICLC from 1 mg to 0.9 mg.<br>• Revised data on the number of patients who have received CDX1401 and the reference to adverse drug reactions that are considered expected for the purposes of expedited reporting.<br>• Revised the reference to adverse drug reactions that are considered expected for the purposes of expedited reporting for CDX-1127.<br>24. Section 8.5: Revised version of CTCAE from 4.03 to 5.<br>25. Section 8.6.4: Revised reporting guidelines to include reporting all SAEs through 30 days post-treatment to Celldex.<br>26. Section 9.1:<br>• Removed CDX-1127 administration on days 15 and 57.<br>• Removed β-HCG testing on days 15 and 57.<br>• Removed HaHa testing as a separate row in the tables. This testing will be completed using the serum collected as part of the research bloods.<br>• Combined green top tubes and red top tubes into one row labeled "Research bloods"<br>• Added a row for archival tissue for NY-ESO-1 expression<br>27. References: Added reference 128.
1/22/18	1. Updated list of study personnel by adding Varinder Kaur, Adela Mahmutovic and Meagan Darling and removing Alexandra Carney, Annika Shuali, and Kendall Pettaway<br>2. Revised Table of Contents<br>3. Removed references to drug sources from the protocol synopsis and Section 1.2 (Study Rationale)<br>4. Corrected references throughout the document<br>5. Revised Section 1.2 (Study Rationale) to include more information about the impact of CD27 and CD70 and the human experience with the CD27 antibody (CDX-1127).<br>6. Revised Section 1.2 (Study Rationale) to include the hypotheses that **agonism of CD27, combined with a toll-like receptor (TLR) agonist and helper peptide vaccine, will decrease regulatory T cells, optimize antitumor T cell induction, and improve T cell survival** as a goal in the present study.

	7. Added a statement in Section 1.6.3 (Human Experience for polyICLC) that polyICLC has been safety used in cancer patients with intramuscular doses up to 50 mcg/kg.<br>8. Removed Figure 6 (Changes in T-regs after CD27 antibody CDX-1127) from section 1.7.3 (Previous Human Experience for CDX-1127)<br>9. Modified Section 3.1 (Inclusion Criteria) to state that subjects with brain metastases are eligible if all of the following are true:<br>• Each brain metastasis must have been completely removed by surgery or each unresected brain metastasis must have been treated with stereotactic radiosurgery.<br>• ~~There has been no evident growth of any brain metastases since the most recent treatment.~~<br>• No brain metastasis is > 2 cm in diameter at the time of registration.<br>• **Any neurologic symptoms attributable to brain metastases have returned to baseline.**<br>• **There is no evidence of new or enlarging brain metastases.**<br>10. Modified Section 3.2 (Exclusion Criteria) bullet 7 as follows: Males and females must agree, in the consent form, to use effective birth control methods during the course of **active study treatment and for up to 70 days after the last dose of study treatment** ~~vaccination~~.<br>11. Revised Section 3.2 (Exclusion Criteria) bullet 13 to state that in addition to the previously listed autoimmune disorders requiring cytotoxic or immunosuppressive therapy the following will not be exclusionary:<br>• **Resolved childhood asthma/atopy**<br>• **Intermittent use of bronchodilators or local steroid injections**<br>• **Hypothyroidism stable on hormone**<br>• **Replacement for Sjogren's syndrome**<br>12. Revised section 4.0 (Study Drugs) to remove cross-references to IND 10825 and instead reference the IND submission for 10825 since this study will be added as an amendment to the IND.<br>13. Added Section 4.5.3 to state that cyclophosphamide drug accountability will be maintained by the University of Virginia Investigational pharmacy or the Human Immune therapy Center<br>14. Revised Section 5.1 (Management of Participants) to indicate that participants will be off treatment follow-up at about 8 months instead of 7 months.<br>15. Added the following language to section 5.3.3 (Pre-medications): "None required, though oral antihistamines (eg: loratadine), acetaminophen, or NSAIDs may be administered for symptom management."<br>16. Updated section 5.7.1 (Dose delays due to toxicity) to state that if a grade 3 toxicity that is not expected occurs, the mCy (cyclophosphamide) should be discontinued.

	17. Revised section 5.7.2 (Delayed visits for reasons other than toxicity) to state that labs and study treatment/procedures may occur on different days as long as each occurs within the specified established window shown in Tables 5 and 6.<br>18. Revised section 5.13 (Management of Toxicity) to include a reference to the investigator's brochure for the management of CDX-1401 toxicities and the following language "The study will be monitored continuously for treatment-related adverse events. Expected treatment-related toxicities of 6MHP combined with IFA and/or polyICLC, with or without mCy, will be managed in accord with section 5.7.1, which allows for dose delays, but not dose reductions."<br>19. Separated vital signs from the physical exam in section 6.1 (Physical Exam and Evaluations).<br>20. Added the collection of height to the list of evaluations in section 6.1 (Physical Exams and Evaluations).<br>21. Updated section 6.3 (NY-ESO-1 Expression) to state that exploratory serotyping for NY-ESO-1 expression may be performed by Celldex Therapeutics.<br>22. Revised formatting of section 6.5 (Clinical Labs) to list the labs in a detailed table format<br>23. In section 6.9.2 (Procedure) added the following language regarding tumor biopsies: "When appropriate (and we anticipate the majority of cases) the biopsies will be performed under local anesthesia (typically lidocaine HCl 1% and epinephrine 1:100,000 injection + or - 8.4% sodium bicarbonate), in the outpatient clinic or comparable procedure room, using sterile technique. In cases when clinical standard of care requires a larger procedure the biopsies may be performed in the operating room under standard technique. To minimize errors in analysis due to sampling error and specimen heterogeneity, each study biopsy specimen will be divided into several components and randomly allocated into various preservation conditions. Ideally, tissue will be divided into the following preservation conditions, using core needle biopsies (19 mm long and 2 mm diameter; about 80 mm_3), or incisional or excisional biopsies with at least the same minimum tissue volume."<br>24. Removed the language stating "Participants will be referred for an ophthalmologic exam if any of these ocular adverse events occur" from Section 8.5 (Adverse Event Classifications) since this information is provided in section 8.8 (Dose-limiting Toxicities).<br>25. Removed references to LICR from section 8.6.4 (Additional Reporting Requirements for the Sponsor) since the LICR will no longer be supplying polyICLC for the study.<br>26. Removed requirement for 30 minute post-vaccine observation period and collection of vital signs post-vaccine administration from Section 8.7.1 (Capturing Adverse Events). Note that vaccine observation periods are still listed in Sections 5.2.5 and 5.3.5.

	27. Section 8.8 (Recording laboratory Values) was removed and the information was combined into section 6.5 (Clinical Labs).<br>28. Added HaHa testing on Days -6 and 57 in the study calendars for Cohorts 1A and 1B in Section 9.1 (Study Calendars).<br>29. In Section 9.1 (Study Calendars) revised footnote 'g' in all study calendars to indicate that a serum pregnancy or urine pregnancy test is required within 72 hours prior to dosing on Day 1 instead of within 24 hours.<br>30. Added footnote 'i' to all of the study calendars to indicate that physical exam includes height (at screening only), weight, performance status, and neurologic function-general.<br>31. Added a new line in the study calendars in Section 9.1 (Study Calendars) for vital signs. Footnote 'J' was added to indicate that vital signs include temperature, pulse, respiratory rate and blood pressure.<br>32. Footnote 'k' was added to the calendars for Cohorts 1A and 2A to indicate that vital signs should be collected pre-infusion and 1 hour post-infusion on days when subjects receive CDX-1127.<br>33. Revised the Post treatment calendar in Section 9.1 (Study Calendars) to remove chest x-ray and CT of chest/abdomen/pelvis or PET-CT from the study calendar and state that Imaging will be performed as clinically indicated. Footnote 'c' was also created to indicate that "if a subject is discontinued and the assessments have been completed as part of a regularly scheduled visit, the assessments do not need to be repeated."<br>34. Added footnote 'd' to the post treatment calendar in Section 9.1 (Study Calendars) stating "Tumor imaging does not need to be repeated if scans have been completed within the past 8 weeks"
7/20/17	1. Editorial and formatting changes made throughout document<br>2. Updated table of contents<br>3. Replaced references to IFA with Montanide ISA-51 in the primary objective of the Protocol synopsis and in sections 1.9, 2.1, 5.2.1, 7.0, 7.2, and 9.1.<br>4. In Section 1.4.3, a reference to IB section 6.3.2 was made<br>5. Clarified footnote in Section 5.7.1 as follows "*The vaccine includes all components that make up the vaccine (e.g.6MHP ~~or CDX-1401~~ + Montanide ISA-51 ~~+~~**with or without** PolyICLC **or CDX-1401 with or without PolyICLC**)"<br>6. In the Protocol synopsis and section 2.1 (Primary Objectives) revised the Primary objective as follows "Safety: To test the safety of CDX-1127 administered concurrently with either of two melanoma vaccines (CDX-1401 or 6MHP). **The 6MHP vaccine will include Montanide ISA-51.** ~~The~~ **Each** melanoma vaccine~~s~~ will **also** be co-administered with or without polyICLC, ~~with or without incomplete Freund's adjuvant,~~ and with oral metronomic cyclophosphamide."<br>7. In Section 3.3, it was clarified that treatment allocation will occur after registration, and within 1 week of the start of treatment.<br>8. In Section 7.0 added PolyICLC to treatments for cohort 2 in Table 7

	9. Changed references to Appendix 3 and Appendix 4 to Sections 9.3 and 9.4, respectively.<br>10. Deleted LDH from list of recorded laboratory values in section 8.8.<br>11. In section 9.1, a redundant footnote was removed (footnote i) from the study calendars and the collection of 120cc green top tubes was removed from the post-active treatment study calendar.

References Cited

1. Topalian SL, Gonzales MI, Parkhurst M, et al: Melanoma-specific CD4+ T cells recognize nonmutated HLA-DR-restricted tyrosinase epitopes. Journal of Experimental Medicine 183:1965-1971, 1996
2. Kobayashi H, Kokubo T, Sato K, et al: CD4+ T cells from peripheral blood of a melanoma patient recognize peptides derived from nonmutated tyrosinase. Cancer Research 58:296-301, 1998
3. Zarour HM, Kirkwood JM, Kierstead LS, et al: Melan-A/MART-1(51-73) represents an immunogenic HLA-DR4-restricted epitope recognized by melanoma-reactive CD4(+) T cells. Proceedings of the National Academy of Sciences of the United States of America 97:400-405, 2000
4. Manici S, Sturniolo T, Imro MA, et al: Melanoma cells present a MAGE-3 epitope to CD4(+) cytotoxic T cells in association with histocompatibility leukocyte antigen DR11. Journal of Experimental Medicine 189:871-876, 1999
5. Chaux P, Vantomme V, Stroobant V, et al: Identification of MAGE-3 epitopes presented by HLA-DR molecules to CD4+ T lymphocytes. JEM 189:767-777, 1999
6. Halder T, Pawelec G, Kirkin AF, et al: Isolation of novel HLA-DR restricted potential tumor-associated antigens from the melanoma cell line FM3. Cancer Research 57:3238-3244, 1997
7. Li K, Adibzadeh M, Halder T, et al: Tumour-specific MHC-class-II-restricted responses after in vitro sensitization to synthetic peptides corresponding to gp100 and Annexin II eluted from melanoma cells. Cancer Immunology, Immunotherapy 47:32-38, 1998
8. Bhardwaj N, Friedlander PA, Pavlic AC, et al: A Phase II, Open-label, Multicenter, Randomized Study of CDX-1401, a Dendritic Cell Targeting NY-ESO-1 Vaccine, in Patients with Malignant Melanoma Pre-Treated with CDX-301, a Recombinant Human Flt3 Ligand. Presented at the American Society of Clinical Oncology Annual Meeting June 2016, Chicago, IL, 2016
9. Aung PP, Liu YC, Ballester LY, et al: Expression of New York esophageal squamous cell carcinoma-1 in primary and metastatic melanoma. Hum Pathol 45:259-67, 2014
10. Giavina-Bianchi M, Giavina-Bianchi P, Sotto MN, et al: Increased NY-ESO-1 expression and reduced infiltrating CD3+ T cells in cutaneous melanoma. J Immunol Res 2015:761378, 2015
11. Park TS, Groh EM, Patel K, et al: Expression of MAGE-A and NY-ESO-1 in Primary and Metastatic Cancers. J Immunother 39:1-7, 2016
12. Jazirehi AR, Lim A, Dinh T: PD-1 inhibition and treatment of advanced melanoma-role of pembrolizumab. Am J Cancer Res 6:2117-2128, 2016
13. Zhou TC, Sankin AI, Porcelli SA, et al: A review of the PD-1/PD-L1 checkpoint in bladder cancer: From mediator of immune escape to target for treatment. Urol Oncol 35:14-20, 2017
14. Gentzler R, Hall R, Kunk PR, et al: Beyond melanoma: inhibiting the PD-1/PD-L1 pathway in solid tumors. Immunotherapy 8:583-600, 2016
15. Bullock TN, Yagita H: Induction of CD70 on dendritic cells through CD40 or TLR stimulation contributes to the development of CD8+ T cell responses in the absence of CD4+ T cells. J. Immunol 174:710-717, 2005
16. Dong H, Franklin NA, Roberts DJ, et al: CD27 stimulation promotes the frequency of IL-7 receptor-expressing memory precursors and prevents IL-12-mediated loss of CD8(+) T cell memory in the absence of CD4(+) T cell help. J. Immunol 188:3829-3838, 2012
17. Peperzak V, Veraar EA, Keller AM, et al: The Pim kinase pathway contributes to survival signaling in primed CD8+ T cells upon CD27 costimulation. J Immunol 185:6670-6678, 2010
18. Roberts DJ, Franklin NA, Kingeter LM, et al: Control of established melanoma by CD27 stimulation is associated with enhanced effector function and persistence, and reduced PD-1 expression of tumor infiltrating CD8(+) T cells. J Immunother 33:769-779, 2010
19. Isogawa M, Chung J, Murata Y, et al: CD40 activation rescues antiviral CD8+ T cells from PD-1-mediated exhaustion. PLoS. Pathog 9:e1003490, 2013

20. Infante JR, Burris HA, Ansell SM, et al: Immunologic activity of an activating anti-CD27 antibody (CDX-1127) in patients with solid tumors. J Clin. Oncol 32:abstract 3027, 2014
21. Wei SM, Fei JX, Tao F, et al: Anti-CD27 Antibody Potentiates Antitumor Effect of Dendritic Cell-Based Vaccine in Prostate Cancer-Bearing Mice. Int Surg 100:155-63, 2015
22. Siegel RL, Miller KD, Jemal A: Cancer statistics, 2016. CA Cancer J Clin 66:7-30, 2016
23. Tarhini AA, Gogas H, Kirkwood JM: IFN-alpha in the treatment of melanoma. J Immunol 189:3789-3793, 2012
24. Kirkwood JM, Strawderman MH, Ernstoff MS, et al: Interferon alfa-2b adjuvant therapy of high-risk resected cutaneous melanoma: the Eastern Cooperative Oncology Group Trial EST 1684 [see comments]. Journal of Clinical Oncology 14:7-17, 1996
25. Kirkwood JM, Strawderman MH, Ernstoff MS, et al: Interferon alfa-2b adjuvant therapy of high-risk resected cutaneous melanoma: the Eastern Cooperative Oncology Group Trial EST 1684. J Clin Oncol 14:7-17, 1996
26. Ascierto PA, Gogas HJ, Grob JJ, et al: Adjuvant interferon alfa in malignant melanoma: an interdisciplinary and multinational expert review. Crit Rev. Oncol. Hematol 85:149-161, 2013
27. Agarwala SS, Lee SJ, Flaherty LE, et al: Randomized phase III trial of high-dose interferon alfa-2b (HDI) for 4 weeks induction only in patients with intermediate- and high-risk melanoma (Intergroup trial E 1697) . Journal of Clinical Oncology 29 suppl:Abstract 8505, 2011
28. Grob JJ, Jouary T, Dreno B, et al: Adjuvant therapy with pegylated interferon alfa-2b (36 months) versus low-dose interferon alfa-2b (18 months) in melanoma patients without macro-metastatic nodes: EADO trial. Journal of Clinical Oncology 28:Abstract # LBA 8506, 2010
29. Eggermont AM, Suciu S, Santinami M, et al: Adjuvant therapy with pegylated interferon alfa-2b versus observation alone in resected stage III melanoma: final results of EORTC 18991, a randomised phase III trial. Lancet 372:117-126, 2008
30. Wolchok JD, al. e: Phase III randomized study of ipilimumab (IPI) plus dacarbazine (DTIC) versus DTIC alone as first-line treatment in patients with unresectable stage III or IV melanoma. Journal of Clinical Oncology 29:776s; abstract LBA5, 2011
31. Hodi FS, O'Day SJ, McDermott DF, et al: Improved Survival with Ipilimumab in Patients with Metastatic Melanoma. N. Engl. J Med 363:711-723, 2010
32. Eggermont AM, Chiarion-Sileni V, Grob JJ, et al: Prolonged Survival in Stage III Melanoma with Ipilimumab Adjuvant Therapy. N Engl J Med, 2016
33. Eggermont AM, Chiarion-Sileni V, Grob JJ, et al: Ipilimumab versus placebo after complete resection of stage III melanoma: Initial efficacy and safety results from the EORTC 18071 phase III trial. Journal of Clinical Oncology 32:abstract LBA9008, 2014
34. Eggermont AM, Chiarion-Sileni V, Grob JJ, et al: Adjuvant ipilimumab versus placebo after complete resection of high-risk stage III melanoma (EORTC 18071): a randomised, double-blind, phase 3 trial. Lancet Oncol 16:522-30, 2015
35. Rahma OE, Gammoh E, Simon RM, et al: Is the "3+3" dose-escalation phase I clinical trial design suitable for therapeutic cancer vaccine development? A recommendation for alternative design. Clin Cancer Res 20:4758-67, 2014
36. Slingluff CL, Jr., Petroni GR, Chianese-Bullock KA, et al: Immunologic and clinical outcomes of a randomized phase II trial of two multipeptide vaccines for melanoma in the adjuvant setting. Clinical Cancer Research 13:6386-6395, 2007
37. Chi M, Dudek AZ: Vaccine therapy for metastatic melanoma: systematic review and meta-analysis of clinical trials. Melanoma Res 21:165-74, 2011
38. Rosenberg SA, Yang JC, Restifo NP: Cancer immunotherapy: moving beyond current vaccines. Nature Medicine 10:909-915, 2004
39. Slingluff CL, Jr., Chianese-Bullock KA, Bullock TN, et al: Immunity to melanoma antigens: from self-tolerance to immunotherapy. Adv Immunol 90:243-95, 2006

40. Slingluff CL, Jr., Petroni GR, Chianese-Bullock KA, et al: Randomized multicenter trial of the effects of melanoma-associated helper peptides and cyclophosphamide on the immunogenicity of a multipeptide melanoma vaccine. J Clin Oncol 29:2924-32, 2011
41. Slingluff CL, Jr., Petroni GR, Olson WC, et al: Effect of granulocyte/macrophage colony-stimulating factor on circulating CD8+ and CD4+ T-cell responses to a multipeptide melanoma vaccine: outcome of a multicenter randomized trial. Clin Cancer Res 15:7036-44, 2009
42. Kirkwood JM, Lee S, Land S, et al: E1696: Final analysis of the clinical and immunological results of a multicenter ECOG phase II trial of multi-epitope peptide vaccination for stage IV melanoma with MART-1 (27-35), gp100 (209-217, 210M), and tyrosinase (368-376, 370D) (MGT) +/- IFNa2b and GM-CSF. J Clin Oncol (Meeting Abstracts) 22:7502, 2004
43. Rosenberg SA, Sherry RM, Morton KE, et al: Tumor progression can occur despite the induction of very high levels of self/tumor antigen-specific CD8+ T cells in patients with melanoma. Journal of Immunology 175:6169-6176, 2005
44. Slingluff CL, Jr., Petroni GR, Yamshchikov GV, et al: Immunologic and clinical outcomes of vaccination with a multiepitope melanoma peptide vaccine plus low-dose interleukin-2 administered either concurrently or on a delayed schedule. Journal of Clinical Oncology 22:4474-4485, 2004
45. Schwartzentruber DJ, Lawson DH, Richards JM, et al: gp100 peptide vaccine and interleukin-2 in patients with advanced melanoma. N. Engl. J Med 364:2119-2127, 2011
46. Slingluff CL, Jr., Petroni GR, Olson W, et al: Helper T cell responses and clinical activity of a melanoma vaccine with multiple peptides from MAGE and melanocytic differentiation antigens. Journal of Clinical Oncology 26:4973-4980, 2008
47. Slingluff CL, Jr., Lee S, Zhao F, et al: A randomized phase II trial of multiepitope vaccination with melanoma peptides for cytotoxic T cells and helper T cells for patients with metastatic melanoma (E1602). Clin Cancer Res 19:4228-38, 2013
48. Hu Y, Kim H, Blackwell CM, et al: Long-term outcomes of helper peptide vaccination for metastatic melanoma. Ann Surg 262:456-64; discussion 462-4, 2015
49. Reed CM, Cresce ND, Mauldin IS, et al: Vaccination with Melanoma Helper Peptides Induces Antibody Responses Associated with Improved Overall Survival. Clin Cancer Res 21:3879-87, 2015
50. Dillon PM, Olson WC, Czarkowski A, et al: A melanoma helper peptide vaccine increases Th1 cytokine production by leukocytes in peripheral blood and immunized lymph nodes. J Immunother Cancer 2:23, 2014
51. Hu Y, Petroni GR, Olson WC, et al: Immunologic hierarchy, class II MHC promiscuity, and epitope spreading of a melanoma helper peptide vaccine. Cancer Immunol Immunother 63:779-86, 2014
52. Hu Y, Smolkin ME, White EJ, et al: Inflammatory Adverse Events are Associated with Disease-Free Survival after Vaccine Therapy among Patients with Melanoma. Annals of Surgical Oncology in press, 2014
53. Rosenberg SA, Yang JC, Kammula US, et al: Different adjuvanticity of incomplete freund's adjuvant derived from beef or vegetable components in melanoma patients immunized with a peptide vaccine. J Immunother 33:626-629, 2010
54. Slingluff CL, Jr., Petroni GR, Smolkin ME, et al: Immunogenicity for $CD8^+$ and $CD4^+$ T cells of two formulations of an incomplete Freund's adjuvant for multipeptide melanoma vaccines. Journal of Immunotherapy 33:630-638, 2010
55. Hailemichael Y, Dai Z, Jaffarzad N, et al: Persistent antigen at vaccination sites induces tumor-specific CD8(+) T cell sequestration, dysfunction and deletion. Nat. Med 19:465-472, 2013
56. Salerno EP, Shea SM, Olson WC, et al: Activation, dysfunction and retention of T cells in vaccine sites after injection of incomplete Freund's adjuvant, with or without peptide. Cancer Immunol Immunother 62:1149-59, 2013

57. Matsuzaki J, Tsuji T, Luescher IF, et al: Direct tumor recognition by a human CD4(+) T-cell subset potently mediates tumor growth inhibition and orchestrates anti-tumor immune responses. Sci Rep 5:14896, 2015
58. Chen JL, Dawoodji A, Tarlton A, et al: NY-ESO-1 specific antibody and cellular responses in melanoma patients primed with NY-ESO-1 protein in ISCOMATRIX and boosted with recombinant NY-ESO-1 fowlpox virus. Int J Cancer 136:E590-601, 2015
59. Robbins PF, Kassim SH, Tran TL, et al: A pilot trial using lymphocytes genetically engineered with an NY-ESO-1-reactive T-cell receptor: long-term follow-up and correlates with response. Clin Cancer Res 21:1019-27, 2015
60. Chen YT, Boyer AD, Viars CS, et al: Genomic cloning and localization of CTAG, a gene encoding an autoimmunogenic cancer-testis antigen NY-ESO-1, to human chromosome Xq28. Cytogenetics & Cell Genetics 79:237-240, 1997
61. Cruz LJ, Rosalia RA, Kleinovink JW, et al: Targeting nanoparticles to CD40, DEC-205 or CD11c molecules on dendritic cells for efficient CD8(+) T cell response: a comparative study. J Control Release 192:209-18, 2014
62. Tsuji T, Matsuzaki J, Kelly MP, et al: Antibody-targeted NY-ESO-1 to mannose receptor or DEC-205 in vitro elicits dual human CD8+ and CD4+ T cell responses with broad antigen specificity. J Immunol 186:1218-27, 2011
63. Birkholz K, Schwenkert M, Kellner C, et al: Targeting of DEC-205 on human dendritic cells results in efficient MHC class II-restricted antigen presentation. Blood 116:2277-85, 2010
64. Dhodapkar MV, Sznol M, Zhao B, et al: Induction of antigen-specific immunity with a vaccine targeting NY-ESO-1 to the dendritic cell receptor DEC-205. Sci Transl Med 6:232ra51, 2014
65. Hamid O, Robert C, Daud A, et al: Safety and tumor responses with lambrolizumab (anti-PD-1) in melanoma. N Engl J Med 369:134-44, 2013
66. Brahmer JR, Tykodi SS, Chow LQ, et al: Safety and activity of anti-PD-L1 antibody in patients with advanced cancer. N. Engl. J. Med 366:2455-2465, 2012
67. Topalian SL, Hodi FS, Brahmer JR, et al: Safety, activity, and immune correlates of anti-PD-1 antibody in cancer. N Engl J Med 366:2443-54, 2012
68. Larkin J, Chiarion-Sileni V, Gonzalez R, et al: Combined Nivolumab and Ipilimumab or Monotherapy in Untreated Melanoma. N Engl J Med 373:23-34, 2015
69. Peperzak V, Veraar EA, Xiao Y, et al: CD8+ T Cells Produce the Chemokine CXCL10 in Response to CD27/CD70 Costimulation To Promote Generation of the CD8+ Effector T Cell Pool. J Immunol, 2013
70. Taraban VY, Rowley TF, Al-Shamkhani A: Cutting edge: a critical role for CD70 in CD8 T cell priming by CD40-licensed APCs. J Immunol 173:6542-6, 2004
71. Hendriks J, Gravestein LA, Tesselaar K, et al: CD27 is required for generation and long-term maintenance of T cell immunity. Nat. Immunol 1:433-440, 2000
72. Soares H, Waechter H, Glaichenhaus N, et al: A subset of dendritic cells induces CD4+ T cells to produce IFN-gamma by an IL-12-independent but CD70-dependent mechanism in vivo. J Exp Med 204:1095-106, 2007
73. Ahrends T, Babala N, Xiao Y, et al: CD27 agonism plus PD-1 blockade recapitulates CD4+ T cell help in therapeutic anti-cancer vaccination. Cancer Res, 2016
74. Borst J, Hendriks J, Xiao Y: CD27 and CD70 in T cell and B cell activation. Curr Opin Immunol 17:275-81, 2005
75. Ochsenbein AF, Riddell SR, Brown M, et al: CD27 expression promotes long-term survival of functional effector-memory CD8+ cytotoxic T lymphocytes in HIV-infected patients. J Exp Med 200:1407-1417, 2004
76. Akiba H, Nakano H, Nishinaka S, et al: CD27, a member of the tumor necrosis factor receptor superfamily, activates NF-kappaB and stress-activated protein kinase/c-Jun N-terminal kinase via TRAF2, TRAF5, and NF-kappaB-inducing kinase. J Biol Chem 273:13353-8, 1998

77. Arch RH, Gedrich RW, Thompson CB: Translocation of TRAF proteins regulates apoptotic threshold of cells. Biochem Biophys Res Commun 272:936-45, 2000
78. Gravestein LA, Amsen D, Boes M, et al: The TNF receptor family member CD27 signals to Jun N-terminal kinase via Traf-2. Eur J Immunol 28:2208-16, 1998
79. Boursalian TE, McEarchern JA, Law CL, et al: Targeting CD70 for human therapeutic use. Adv. Exp Med Biol 647:108-19. doi: 10.1007/978-0-387-89520-8_7.:108-119, 2009
80. Pen JJ, De KB, Maenhout SK, et al: Modulation of regulatory T cell function by monocyte-derived dendritic cells matured through electroporation with mRNA encoding CD40 ligand, constitutively active TLR4, and CD70. J Immunol 191:1976-1983, 2013
81. Coquet JM, Ribot JC, Babala N, et al: Epithelial and dendritic cells in the thymic medulla promote CD4+Foxp3+ regulatory T cell development via the CD27-CD70 pathway. J Exp Med 210:715-728, 2013
82. Claus C, Riether C, Schurch C, et al: CD27 signaling increases the frequency of regulatory T cells and promotes tumor growth. Cancer Res 72:3664-3676, 2012
83. Burris HA, Infante JR, Ansell SM, et al: Safety and Activity of Varlilumab, a Novel and First-in-Class Agonist Anti-CD27 Antibody, in Patients With Advanced Solid Tumors. J Clin Oncol 35:2028-2036, 2017
84. Thompson LW, Hogan KT, Caldwell JA, et al: Preventing the spontaneous modification of an HLA-A2-restricted peptide at an N-terminal glutamine or an internal cysteine residue enhances peptide antigenicity. Journal of Immunotherapy 27:177-183, 2004
85. Touloukian CE, Leitner WW, Topalian SL, et al: Identification of a MHC class II-restricted human gp100 epitope using DR4-IE transgenic mice. Journal of Immunology 164:3535-3542, 2000
86. Faries MB, Hsueh EC, Ye X, et al: Effect of granulocyte/macrophage colony-stimulating factor on vaccination with an allogeneic whole-cell melanoma vaccine. Clin. Cancer Res 15:7029-7035, 2009
87. Korn EL, Liu PY, Lee SJ, et al: Meta-analysis of phase II cooperative group trials in metastatic stage IV melanoma to determine progression-free and overall survival benchmarks for future phase II trials. J Clin Oncol 26:527-534, 2009
88. Rosenberg SA, White DE: Vitiligo in patients with melanoma: normal tissue antigens can be targets for cancer immunotherapy. Journal of Immunotherapy with Emphasis on Tumor Immunology 19:81-84, 1996
89. Kawakami Y: Immunobiology of human melanoma antigens MART-1 and gp100 and their use for Immuno-gene therapy. Int. Rev. Immunol 142:173-192, 1997
90. Dudley ME, Wunderlich JR, Robbins PF, et al: Cancer regression and autoimmunity in patients after clonal repopulation with antitumor lymphocytes. Science 298:850-854, 2002
91. Bonifaz LC, Bonnyay DP, Charalambous A, et al: In vivo targeting of antigens to maturing dendritic cells via the DEC-205 receptor improves T cell vaccination. J Exp Med 199:815-24, 2004
92. Ramakrishna V, Vasilakos JP, Tario JD, Jr., et al: Toll-like receptor activation enhances cell-mediated immunity induced by an antibody vaccine targeting human dendritic cells. J Transl Med 5:5, 2007
93. Scalzo AA, Elliott SL, Cox J, et al: Induction of protective cytotoxic T cells to murine cytomegalovirus by using a nonapeptide and a human-compatible adjuvant (Montanide ISA 720). Journal of Virology 69:1306-1309, 1995
94. Fernandez IM, Snijders A, Benaissa-Trouw BJ, et al: Influence of epitope polarity and adjuvants on the immunogenicity and efficacy of a synthetic peptide vaccine against Semliki Forest virus. Journal of Virology 67:5843-8, 1993
95. Ahlers JD, Dunlop N, Alling DW, et al: Cytokine-in-adjuvant steering of the immune response phenotype to HIV-1 vaccine constructs: granulocyte-macrophage colony-stimulating factor and TNF-alpha synergize with IL-12 to enhance induction of cytotoxic T lymphocytes. Journal of Immunology 158:3947-3958, 1997

96. Schaefer JT, Patterson JW, Deacon DH, et al: Dynamic changes in cellular infiltrates with repeated cutaneous vaccination: a histologic and immunophenotypic analysis. J Transl Med 8:79, 2010
97. Lore K, Betts MR, Brenchley JM, et al: Toll-like receptor ligands modulate dendritic cells to augment cytomegalovirus- and HIV-1-specific T cell responses. J Immunol 171:4320-4328, 2003
98. Perrot I, Deauvieau F, Massacrier C, et al: TLR3 and Rig-like receptor on myeloid dendritic cells and Rig-like receptor on human NK cells are both mandatory for production of IFN-gamma in response to double-stranded RNA. J Immunol 185:2080-2088, 2010
99. Salazar AM, Levy HB, Ondra S, et al: Long-term treatment of malignant gliomas with intramuscularly administered polyinosinic-polycytidylic acid stabilized with polylysine and carboxymethylcellulose: an open pilot study. Neurosurgery 38:1096-1103, 1996
100. Levy H, Salazar A: Interferon inducers, in Baron S, Copenhaver D, Dianzani F, et al (eds): Interferon: Principles and Medical Applications. Galveston, TX, Galveston University, University of Texas Medical Branch, 1992, pp 65-76
101. Sabbatini P, Tsuji T, Ferran L, et al: Phase I trial of overlapping long peptides from a tumor self-antigen and poly-ICLC shows rapid induction of integrated immune response in ovarian cancer patients. Clin Cancer Res 18:6497-508, 2012
102. Berd D, Maguire HC, Jr., Mastrangelo MJ: Induction of cell-mediated immunity to autologous melanoma cells and regression of metastases after treatment with a melanoma cell vaccine preceded by cyclophosphamide. Cancer Research 46:2572-2577, 1986
103. Proietti E, Greco G, Garrone B, et al: Importance of Cyclophosphamide-induced Bystander Effect on T Cells for a Successful Tumor Eradication in Response to Adoptive Immunotherapy in Mice.[Miscellaneous Article]. Journal of Clinical Investigation 101:429-441, 1998
104. Machiels JP, Reilly RT, Emens LA, et al: Cyclophosphamide, doxorubicin, and paclitaxel enhance the antitumor immune response of granulocyte/macrophage-colony stimulating factor-secreting whole-cell vaccines in HER-2/neu tolerized mice. Cancer Research 61:3689-3697, 2001
105. Berd D, Maguire HC, Jr., Mastrangelo MJ: Potentiation of human cell-mediated and humoral immunity by low-dose cyclophosphamide. Cancer Res 44:5439-5443, 1984
106. Sahasrabudhe DM, deKernion JB, Pontes JE, et al: Specific immunotherapy with suppressor function inhibition for metastatic renal cell carcinoma. J. Biol. Response Mod 5:581-594, 1986
107. Matar P, Rozados VR, Gervasoni SI, et al: Th2/Th1 switch induced by a single low dose of cyclophosphamide in a rat metastatic lymphoma model. Cancer Immunol. Immunother 50:588-596, 2002
108. Matar P, Rozados VR, Gonzalez AD, et al: Mechanism of antimetastatic immunopotentiation by low-dose cyclophosphamide. Eur. J. Cancer 36:1060-1066, 2000
109. Schiavoni G, Mattei F, Di PT, et al: Cyclophosphamide induces type I interferon and augments the number of CD44(hi) T lymphocytes in mice: implications for strategies of chemoimmunotherapy of cancer. Blood 95:2024-2030, 2000
110. Awwad M, North RJ: Cyclophosphamide (Cy)-facilitated adoptive immunotherapy of a Cy-resistant tumour. Evidence that Cy permits the expression of adoptive T-cell mediated immunity by removing suppressor T cells rather than by reducing tumour burden. Immunology 65:87-92, 1988
111. Berd D, Mastrangelo MJ: Effect of low dose cyclophosphamide on the immune system of cancer patients: depletion of CD4+, 2H4+ suppressor-inducer T-cells. Cancer Res 48:1671-5, 1988

112. Hoon DS, Foshag LJ, Nizze AS, et al: Suppressor cell activity in a randomized trial of patients receiving active specific immunotherapy with melanoma cell vaccine and low dosages of cyclophosphamide. Cancer Res 50:5358-64, 1990
113. North RJ: Cyclophosphamide-facilitated adoptive immunotherapy of an established tumor depends on elimination of tumor-induced suppressor T cells. J Exp Med 155:1063-74, 1982
114. Loeffler M, Kruger JA, Reisfeld RA: Immunostimulatory effects of low-dose cyclophosphamide are controlled by inducible nitric oxide synthase. Cancer Res 65:5027-5030, 2005
115. Lutsiak ME, Semnani RT, De PR, et al: Inhibition of CD4(+)25+ T regulatory cell function implicated in enhanced immune response by low-dose cyclophosphamide. Blood 105:2862-2868, 2005
116. Tanaka H, Matsushima H, Mizumoto N, et al: Classification of chemotherapeutic agents based on their differential in vitro effects on dendritic cells. Cancer Res 69:6978-6986, 2009
117. Salem ML, Kadima AN, El-Naggar SA, et al: Defining the ability of cyclophosphamide preconditioning to enhance the antigen-specific CD8+ T-cell response to peptide vaccination: creation of a beneficial host microenvironment involving type I IFNs and myeloid cells. J. Immunother 30:40-53, 2007
118. Ercolini AM, Ladle BH, Manning EA, et al: Recruitment of latent pools of high-avidity CD8(+) T cells to the antitumor immune response. J. Exp. Med 201:1591-1602, 2005
119. Liu JY, Wu Y, Zhang XS, et al: Single administration of low dose cyclophosphamide augments the antitumor effect of dendritic cell vaccine. Cancer Immunol. Immunother 56:1597-1604, 2007
120. Ghiringhelli F, Larmonier N, Schmitt E, et al: CD4+CD25+ regulatory T cells suppress tumor immunity but are sensitive to cyclophosphamide which allows immunotherapy of established tumors to be curative. Eur. J. Immunol 34:336-344, 2004
121. Berd D, Maguire HC, Jr., Mastrangelo MJ: Impairment of concanavalin A-inducible suppressor activity following administration of cyclophosphamide to patients with advanced cancer. Cancer Res 44:1275-80, 1984
122. Emens LA, Asquith JM, Leatherman JM, et al: Timed sequential treatment with cyclophosphamide, doxorubicin, and an allogeneic granulocyte-macrophage colony-stimulating factor-secreting breast tumor vaccine: a chemotherapy dose-ranging factorial study of safety and immune activation. J Clin Oncol 27:5911-5918, 2009
123. Audia S, Nicolas A, Cathelin D, et al: Increase of CD4+ CD25+ regulatory T cells in the peripheral blood of patients with metastatic carcinoma: a Phase I clinical trial using cyclophosphamide and immunotherapy to eliminate CD4+ CD25+ T lymphocytes. Clin. Exp. Immunol 150:523-530, 2007
124. Peretz Y, Sauve D, Gagnon D, et al: Immune profiling of patients vaccinated with the survivin targeted therapeutic vaccine DPX-Survivac demonstrates durable polyfunctional CD4+ and CD8+ T cells in ovarian cancer patients,
125. Mross K, Steinbild S: Metronomic anti-cancer therapy - an ongoing treatment option for advanced cancer patients. Journal of Cancer Therapeutics and Research, 2012
126. Lapi F, Kezouh A, Suissa S, et al: The use of inhaled corticosteroids and the risk of adrenal insufficiency. Eur. Respir. J 42:79-86, 2013
127. Ernst P, Suissa S: Systemic effects of inhaled corticosteroids. Curr. Opin. Pulm. Med 18:85-89, 2012
128. Weber J, Mandala M, et al: Adjuvant Nivolumab versus Ipilimumab in resected Stage III or IV Melanoma. N. Engl. J Med 377:1824-1835, 2017
